# Faster Synthesis of Beta-Diketonate Ternary Europium Complexes: Elapsed Times & Reaction Yields

**DOI:** 10.1371/journal.pone.0143998

**Published:** 2015-12-28

**Authors:** Nathalia B. D. Lima, Anderson I. S. Silva, P. C. Gerson, Simone M. C. Gonçalves, Alfredo M. Simas

**Affiliations:** Departamento de Química Fundamental, Universidade Federal de Pernambuco, Recife, Pernambuco, Brazil; University of Edinburgh, UNITED KINGDOM

## Abstract

β-diketonates are customary bidentate ligands in highly luminescent ternary europium complexes, such as Eu(β-diketonate)_3_(L)_2_, where L stands for a nonionic ligand. Usually, the syntheses of these complexes start by adding, to an europium salt such as EuCl_3_(H_2_O)_6_, three equivalents of β-diketonate ligands to form the complexes Eu(β-diketonate)_3_(H_2_O)_2_. The nonionic ligands are subsequently added to form the target complexes Eu(β-diketonate)_3_(L)_2_. However, the Eu(β-diketonate)_3_(H_2_O)_2_ intermediates are frequently both difficult and slow to purify by recrystallization, a step which usually takes a long time, varying from days to several weeks, depending on the chosen β-diketonate. In this article, we advance a novel synthetic technique which does not use Eu(β-diketonate)_3_(H_2_O)_2_ as an intermediate. Instead, we start by adding 4 equivalents of a monodentate nonionic ligand L straight to EuCl_3_(H_2_O)_6_ to form a new intermediate: EuCl_3_(L)_4_(H_2_O)_n_, with n being either 3 or 4. The advantage is that these intermediates can now be easily, quickly, and efficiently purified. The β-diketonates are then carefully added to this intermediate to form the target complexes Eu(β-diketonate)_3_(L)_2_. For the cases studied, the 20-day average elapsed time reduced to 10 days for the faster synthesis, together with an improvement in the overall yield from 42% to 69%.

## Introduction

Luminescence of europium complexes has a wide range of applications, such as pH sensing based on a modified nanoscale MOF [1]; in the determination of *enterobacter cloacae* labeled DNA probe based on time-resolved fluorescence [2]; as redox-active magnetic resonance imaging (MRI) sensors [3]; in the determination of DNA using the gatifloxacin europium (III) complex [4]; as sensor to visualize latent fingerprints [5]; as emissive nano-confined system for highly-luminescent bioimaging in vivo [6], etc.

In particular, β-diketonate ligands have proved to be efficient antennae leading to highly efficient complexes, with relevant and unique applications such as in emitting layers to fabricate white organic light-emitting diodes (WOLED)[7]; as triple-layer organic light emitting diodes (OLED) in UV portable dosimeter [8]; as red phosphors applied in light-emitting diodes (LED)[9–12]; as turn-on sensors for detecting basic molecules hosted in nanozeolite [13], as surfactant functionalized in polymer for luminescence integration [14], as highly sensitive temperature sensors [15], as two-photon sensitized luminescent probes for applications in biological imaging [16], etc.

Beta-diketonate lanthanide complexes are known since the end of the 19^th^ century when they were first synthesized and reported by Urbain and Budischovsky in 1897 [17]. Rare-earth beta-diketonates have been extensively reviewed by Binnemans, both as complexes [18] and, more especially so, as lanthanide-based organic-inorganic hybrid materials [19,20].

There are three main types of lanthanide beta-diketonate complexes: tris complexes, Lewis base adducts of the tris complexes which are the ternary lanthanide beta-diketonates and tetrakis complexes.

Synthesis of ternary europium β-diketonate complexes are usually carried out by first reacting β-diketonates with either hydrated chloride or hydrated nitrate salts. Unlike chloride ions, which are a good leaving group, nitrate ions coordinate more strongly, in a bidentate manner, which often leads to final complexes containing a coordinating nitrate group [18]. Therefore, most of the europium β-diketonate complexes are prepared using EuCl_3_(H_2_O)_6_, to yield Eu(β-diketonate)_3_(H_2_O)_2_ [18]. Of course, the presence of the coordinated water molecules tends to quench the luminescence due to non-radiative processes, as expected from the presence of near resonance oscillators. Replacing the water molecules with other non-ionic ligands tends to intensify the quantum yield, rendering the europium complexes highly luminescent. Accordingly, the water molecules are subsequently displaced by other non-ionic ligands, such as sulfoxides, phosphine oxides, nitrogen-containing polycyclic aromatic compounds, etc, to obtain the target complexes.

However, the step involved in the purification through recrystallization of the intermediate Eu(β-diketonate)_3_(H_2_O)_2_, which sometimes is obtained in the form of a viscous oil [18], is normally too time consuming. Indeed the recrystallization of only a few milligrams may take from days to several weeks depending on the β-diketonate involved, more especially so for the case of complexes of highly fluorinated ligands [18]. Recently, β-diketonate lanthanide complexes were prepared by mixing a lanthanide salt LnX_3_(H_2_O)_6_ with non-ionic nitrogen coordinating ligands, L, in methanol, to first obtain an intermediate of the generic formula Ln(L)_*l*_X_3_(CH_3_OH)_m_, where Ln may be Eu^3+^, Sm^3+^ or Gd^3+^; *l* may be either 1 or 2; X may be Cl^-^ or NO_3_^-^; and m may be either 0 or 2.[21]. Subsequently, a methanol solution was added containing 1,1,1-trifluoro-2,4-pentanedione, acacF_3_H, and sodium hydroxide to a methanol solution of the intermediate complex to obtain the final product Ln(L)_*l*_X_n_(CH_3_OH)_m_(acacF_3_)_3-n_ where n may be 0 or 1 [21]. These intermediates and final products are insoluble in methanol, a property that facilitates their purification. Therefore, in both steps of the syntheses, the respective intermediates and final products precipitate, are filtered out and subsequently purified by recrystallization in 2-propanol. However, acacF_3_H complexes of europium tend to lead to very small quantum yields, ranging from 0.28% to 1.5%[21].

On the other hand, ligands that lead to highly luminescent complexes are usually soluble in methanol or ethanol, a property that makes their purification more difficult. Much more arduous and critically so, is the case of the intermediate Eu(β-diketonate)_3_(H_2_O)_2_, whose purification may take up months.

In this article, we advance a strategy to considerably shorten the time involved in the overall synthesis of known highly luminescent β-diketonate europium complexes, whose photophysical properties have already been reported [22–26], by first adding the non-ionic ligand to the original salt, EuCl_3_(H_2_O)_6_[27], to obtain the intermediates EuCl_3_(L)_4_(H_2_O)_n_, which are soluble in solvents such as methanol or ethanol. Subsequently, the β-diketonate ligands are carefully added to the intermediate to obtain the final product Eu(β-diketonate)_3_(L)_2_.

Hence, a process, which usually takes a few weeks, can now be carried out in days. Finally, when compared to the usual synthesis, we show that the overall synthetic yield of this faster synthesis of europium β-diketonate complexes is significantly larger.

## Methods

The reagents and solvents used were: 1-(2-thenoyl),3,3,3-trifluoroacetone (TTA), Alfa Aesar, 99%; 1,3-diphenylpropane-1,3-dione (DBM), Alfa Aesar, 98%; DibenzylSulfoxide (DBSO), Sigma Aldrich, 99%; p-Tolylsulfoxide (PTSO), Sigma Aldrich, 99%; Triphenylphosphine oxide (TPPO), Sigma Aldrich, 99%; Ethanol, J. T. Baker; Hexane, Sigma Aldrich; Acetone, Sigma Aldrich; Europium Oxide, Alfa Aesar. The reagents and solvents were used directly from the bottle. The salt EuCl_3_(H_2_O)_6_ was prepared from Eu_2_O_3_ by a well-known method.

### Faster Synthesis

#### Intermediate complex EuCl_3_(L)_4_(H_2_O)_n_

A solution of EuCl_3_(H_2_O)_6_ (0.37 g, 1 mmol) in 250 mL of pure ethanol was prepared under stirring conditions. To this solution, we slowly added the non-ionic ligand L, DBSO (0.92 g, 4 mmol), PTSO (0.92 g, 4 mmol) or TPPO (1.11 g, 4 mmol), which was previously dissolved in 50 mL of ethanol. Then the system was left overnight being stirred under reflux at 78°C. Subsequently, the solvent was rotaevaporated. A solid of EuCl_3_(L)_4_(H_2_O)_n_ (white for L = TPPO and PTSO, and yellow for L = DBSO) was obtained. The complex was recrystallized with a water/EtOH solution (10:1), the supernatant was removed with a Pasteur pipette, and the resulting solid was dried under vacuum for 24h. Yields: 89% for EuCl_3_(TPPO)_4_(H_2_O)_3_, 92% for Eu(DBSO)_4_Cl_3_(H_2_O)_4_ and 86% for Eu(PTSO)_4_Cl_3_(H_2_O)_4_.

#### Target complexes Eu(DBM)_3_(L)_2_ prepared by the faster synthesis

A solution of intermediate complex, EuCl_3_(TPPO)_4_(H_2_O)_3_ (0.28g, 0.2 mmol), EuCl_3_(DBSO)_4_(H_2_O)_4_ (0.25g, 0.2 mmol) or EuCl_3_(PTSO)_4_(H_2_O)_4_ (0.25g, 0.2 mmol), in 30 mL of ethanol was prepared and, at the same time, the β-diketonateK, TTAK (0.16 g, 0.6 mmol) or DBMK (0.16 g, 0.6 mmol) salt, was prepared by dissolving β-diketonateH, TTAH (0.13 g, 0.6 mmol) or DBMH (0.13 g, 0.6 mmol), in ethanol separately and by slowly adding potassium hydroxide (0.03 g, 0.6 mmol) in ethanol/water solution (10:1) under stirring conditions. Once formed, the β-diketonateK ethanolic solution was added, drop by drop, over the EuCl_3_(L)_4_(H_2_O)_n_ (n = 3 for L = TPPO and n = 4 for L = PTSO or DBSO) solution. The pH was checked and, if it was still acidic, a few more drops of the ethanolic solution of KOH were added until the pH stabilized around 6.0~6.5. After at least 30 minutes of stirring, a white precipitate of potassium chloride salt was formed, which was subsequently removed by filtration. If small amounts of the yellow complex were present in the filtered salt, they were washed out with ~15mL of pure ethanol. The filtrate is a yellow solution of the complex and reactants. Next, the filtrate was stirred under reflux at 78°C for 24 h to complete the reaction until a yellow solution of Eu(β-diketonate)_3_(L)_2_ is obtained. Then, the solvent was allowed to slowly evaporate at room temperature. A yellow solid of Eu(β-diketonate)_3_(L)_2_ was obtained, which could still contain some potassium chloride. That is why it is important to wash the solid with ~30mL of water to remove the remaining salt. In sequence, it is necessary to use boiling hexane to remove the released L ligands. Finally, the complex was recrystallized with hexane/acetone solution (10:1). The supernatant was removed with a Pasteur pipette, and the solid was dried under vacuum for 24h. Yields: 88% for Eu(TTA)_3_(TPPO)_2_, 86% for Eu(DBM)_3_(TPPO)_2_, 70% for Eu(TTA)_3_(DBSO)_2_, 68% for Eu(DBM)_3_(DBSO)_2_, 75% for Eu(TTA)_3_(PTSO)_2_, and 80% for Eu(DBM)_3_(PTSO)_2_.

### Usual Synthesis

#### Intermediate complex Eu(β-diketonate)_3_(H_2_O)_2_

A solution of EuCl_3_(H_2_O)_6_ (0.37 g, 1 mmol) in 250 mL of pure ethanol was prepared under stirring conditions. At the same time, the β-diketonateK, TTAK (0.78 g, 3mmol) or DBMK (0.79 g, 3mmol) salt, were prepared by dissolving 3 mmol β-diketonateH, TTAH (0.67 g, 3mmol) or DBMH (0.67 g, 3mmol), in ethanol and by slowly adding of potassium hydroxide (0.17 g, 3mmol) in ethanol/water solution (10:1). Once formed, the DBMK ethanolic solution was added, drop by drop, to the EuCl_3_(H_2_O)_6_ solution. The pH was checked and, if it was still acidic, a few more drops of the ethanolic solution of KOH were added until the pH stabilized around 6.0~6.5. After at least 30 minutes of stirring, a white precipitate of potassium chloride salt was formed, which was subsequently removed by filtration. If small amounts of the yellow complex were present in the filtered salt, they were washed out with ~15mL of pure ethanol. The filtrate is a yellow solution of the complex and reactants. Then, the filtrate stirred under reflux at 78°C for 24 h to complete the reaction to obtain a solution of Eu(β-diketonate)_3_(H_2_O)_2_. Subsequently, the solvent was allowed to slowly evaporate at room temperature. A yellow solid of Eu(β-diketonate)_3_(H_2_O)_2_ was then obtained, which can still contain some potassium chloride. That is why washing the solid with ~30mL of water, was necessary. Finally, the complex was recrystallized with a hexane/acetone solution (10:1), the supernatant was removed with a Pasteur pipette, and the solid was dried under vacuum for 24h. Yields: 73% for Eu(TTA)_3_(H_2_O)_2_, and 60% for Eu(DBM)_3_(H_2_O)_2_.

#### Target complexes Eu(DBM)_3_(L)_2_ by the usual synthesis

A solution of the usual intermediate complex, Eu(TTA)_3_(H_2_O)_2_ (0.17 g, 0.2 mmol) or Eu(DBM)_3_(H_2_O)_2_ (0.17 g, 0.2 mmol), in 30 mL of pure ethanol was prepared under stirring conditions. Slowly, the non-ionic ligand, DBSO (0.09 g, 0.4 mmol), PTSO (0.09 g, 0.4 mmol) or TPPO (0.11 g, 0.4 mmol), previously dissolved in 30 mL of ethanol was added. Then, the system was left overnight being stirred under reflux at 78°C. Subsequently, the solvent was allowed to slowly evaporate at room temperature. A yellow solid of Eu(β-diketonate)_3_(L)_2_ was then obtained, which we dried under vacuum for 24h. Finally, the complex was recrystallized with a hexane/acetone solution (10:1), the supernatant was removed with a Pasteur pipette, and the solid was dried under vacuum for 24h. Yields: 80% for Eu(TTA)_3_(TPPO)_2_, 51% for Eu(DBM)_3_(TPPO)_2_, 75% for Eu(TTA)_3_(DBSO)_2_, 52% for Eu(DBM)_3_(DBSO)_2_, 65% for Eu(TTA)_3_(PTSO)_2_, and 53% for Eu(DBM)_3_(PTSO)_2_.

### Characterizations

All synthesized complexes, both intermediate and target compounds, have been characterized by elemental analysis, (Perkin-Elmer CHN 2400), infrared spectroscopy (samples of the complexes were prepared as KBr disks and the spectra were measured in a Bruker model IFS 66 spectrophotometer, 4000 cm^-1^–400 cm^-1^), and by ^1^H NMR, ^19^F NMR, and ^31^P NMR (all NMR spectra of all complexes were obtained in CDCl_3_ solutions via a Varian Unity Plus 400 MHz, tuning the probe to each of the nuclei studied). The novel intermediate complexes were further characterized by MALDI-TOF mass spectroscopy taken on an Autoflex 3 Smart Beam Vertical spectrometer by Brucker Daltonics, with α-cyano-4-hydroxycinnamic acid as the matrix.

The Supporting Information presents all details of the experimental characterizations, data, spectra and all relevant spectral attributions for all complexes synthesized in this article.

## Results and Discussion

**Fig 1:**
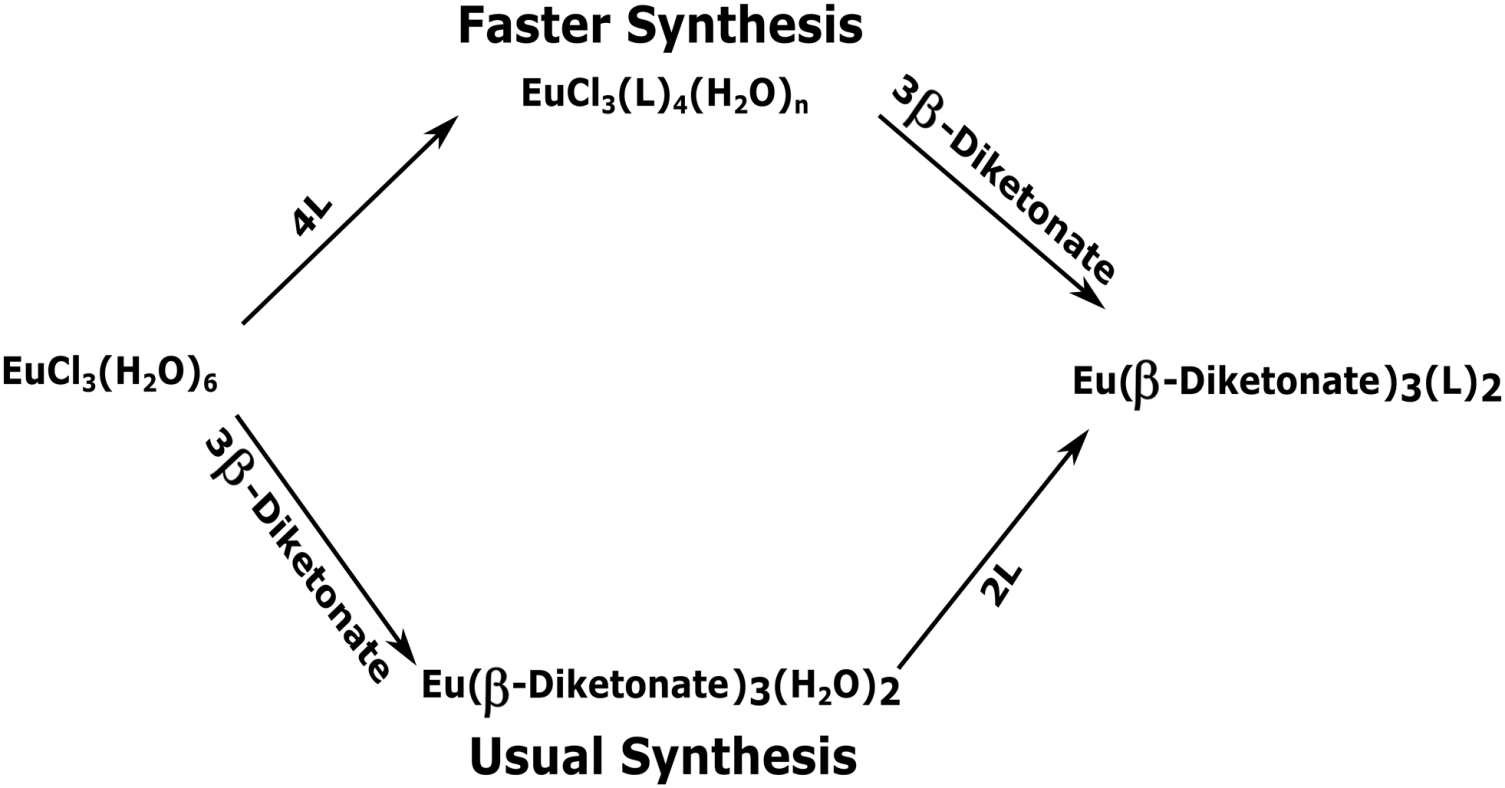
Scheme comparing the faster synthesis of Eu(β-diketonate)_3_(L)_2_ complexes, being advanced in this article, with the usual one; where L stands for a non-ionic ligand.

Fig 1 shows a scheme, comparing our new, faster synthesis of the highly luminescent europium complexes Eu(β-diketonate)_3_(L)_2_ with the usual one.

**Fig 2:**
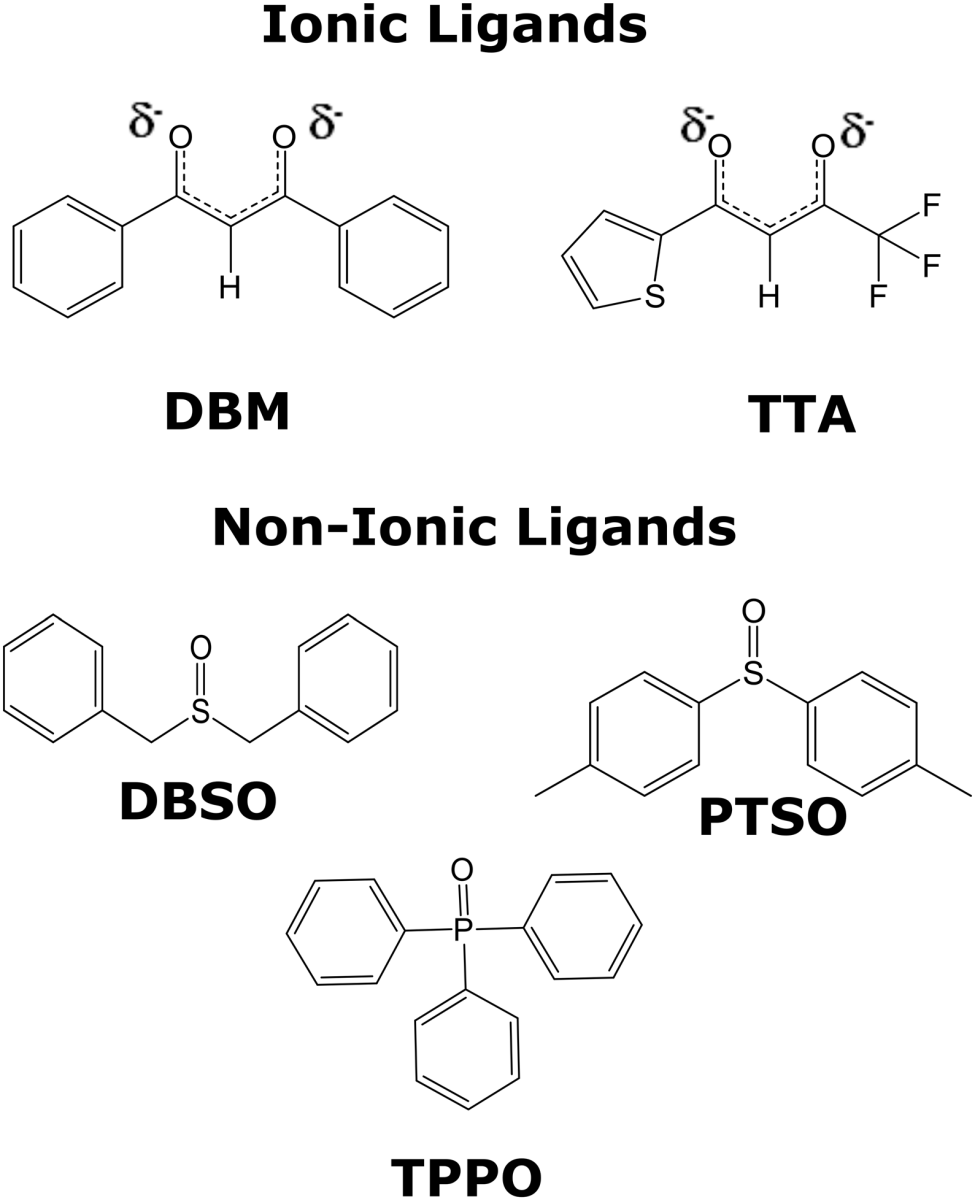
Chemical structures of the ionic ligands (DBM and TTA) and of the non-ionic ligands (TPPO, DBSO and PTSO).

In order to prove that our faster synthesis is indeed feasible, better, and leads to higher overall yields, we compared it with the usual synthesis, both in terms of overall time spent and overall yields. As meaningful examples, we chose to conduct the tests for full combinations of β-diketonates with non-ionic ligands, with the β-diketonates being either 1,3-diphenylpropane-1,3-dione (DBM) or 4,4,4-trifluoro-1-(2-thienyl)-1,3-butanedione (TTA) and with the non-ionic ligands being either triphenylphosphine oxide (TPPO), dibenzyl sulfoxide (DBSO) or 4,4'-sulfinylbis(methylbenzene) (PTSO). Fig 2 shows the chemical structures of these ligands.

Our faster synthesis involves the following sequence of reactions:

$$EuCl_{3}{\left(H_{2}O\right)}_{6}+4L\;\overset{\begin{array}{c} EtOH\\ \sim 78{}^{\circ}C\\ 24h \end{array}}{\to }\;EuCl_{3}{\left(L\right)}_{4}{\left(H_{2}O\right)}_{n}+\left(6-n\right)H_{2}O$$ (1)

$$EuCl_{3}{\left(L\right)}_{4}{\left(H_{2}O\right)}_{n}+3\beta -diketonate.K\;\overset{\begin{array}{c} EtOH\\ \sim 78{}^{\circ}C\\ 24h\\ pH\;\sim 6.5 \end{array}}{\to }\;Eu{\left(\beta -diketonate\right)}_{3}{\left(L\right)}_{2}+2L+3KCl\;+\;nH_{2}O$$ (2)

The first step of the faster synthesis, reaction Eq (1) involves a rotary evaporation of the final ethanolic solution, is very straightforward, and can be carried out in one day. The resulting solution is subsequently dried in a vacuum desiccator for another day. This procedure has been carried out in all three cases studied. It is noteworthy that water is the only product of reaction Eq (1), and therefore its separation with the help of a vacuum desiccator is forthright. Purification of these intermediates is very simple, taking different times depending on the non-ionic ligand, from two days for TPPO to less than one week for DBSO and PTSO, as described in the Experimental section.

The second step of the faster synthesis is also very straightforward. A side solution of β-diketone, stoichiometrically deprotonated by adding potassium hydroxide, is prepared, and then added to a solution of the intermediate complex EuCl_3_(L)_4_(H_2_O)_n_ as in reaction Eq (2). Finally, the pH of the reaction medium was adjusted to 6.5 by carefully adding a warm diluted potassium hydroxide ethanolic solution. Despite the fact that this step leads to three other by products (KCl, L, and H_2_O), these can be easily removed. Indeed, when the reaction completes, the solution is transferred to a beaker, and the solvent (ethanol) is allowed to completely evaporate. Water is then added, and the supernatant with the dissolved KCl, is subsequently removed by means of a Pasteur pipette. The remaining solid precipitate, in the beaker, is then washed thrice with boiling hexane, to dissolve the unbound non-ionic ligands L. The supernatant is once again carefully removed by means of a Pasteur pipette. The purified wet solid in the beaker is finally dried out in a vacuum desiccator to remove the final traces of hexane and water that may have been left out.

**Fig 3:**
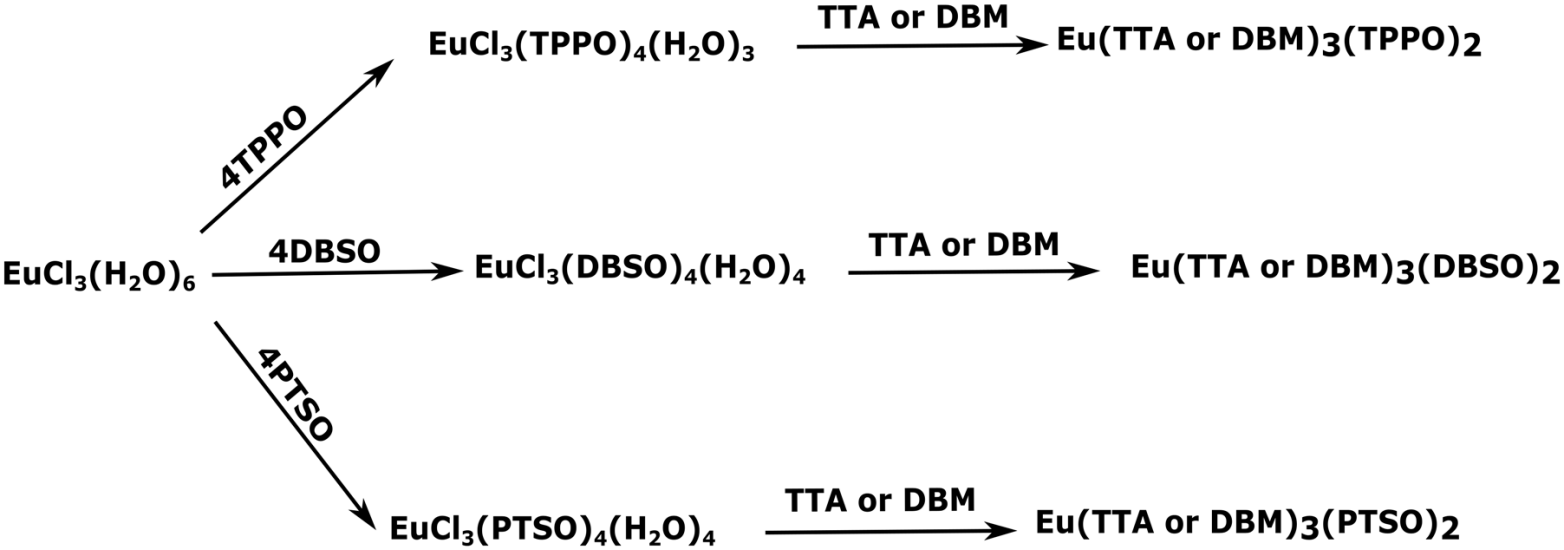
Scheme, showing the novel intermediates and subsequent target complexes, according to the faster synthesis being advanced in this article.

Three novel intermediate complexes have been synthesized and characterized in the first step: EuCl_3_(TPPO)_4_(H_2_O)_3_, EuCl_3_(DBSO)_4_(H_2_O)_4_, and EuCl_3_(PTSO)_4_(H_2_O)_4_. In the second step, the chloride ions were replaced by β-diketonate ligands, yielding the following six luminescent europium complexes: Eu(DBM)_3_(DBSO)_2_[22], Eu(DBM)_3_(PTSO)_2_[22], Eu(DBM)_3_(TPPO)_2_[28], Eu(TTA)_3_(DBSO)_2_[23], Eu(TTA)_3_(TPPO)_2_[25], and Eu(TTA)_3_(PTSO)_2_[26]. Fig 3 shows a scheme with all faster syntheses carried out in this article.

**Fig 4:**
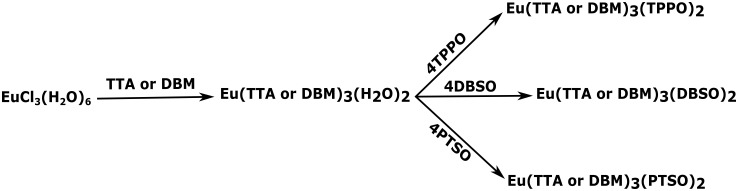
Scheme, showing the usual intermediates and subsequent target complexes, according to the usual synthesis of europium β-diketonate complexes.

In order to prove that our faster synthesis is indeed superior to the usual one, exactly the same target complexes Eu(β-diketonate)_3_(L)_2_ were also synthesized by the usual route, according to Fig 4.

As such, we carried out the usual synthesis of complexes of the type Eu(β-diketonate)_3_(L)_2_ according to the following sequence of reactions:

$$EuCl_{3}{\left(H_{2}O\right)}_{6}+3\beta -diketonate.K\;\;\overset{\begin{array}{c} EtOH\\ \sim 78{}^{\circ}C\\ 24h\\ pH\;\sim 6.5 \end{array}}{\to }\;Eu{\left(\beta -diketonate\right)}_{3}{\left(H_{2}O\right)}_{2}+3KCl+4H_{2}O$$ (3)

$$Eu{\left(\beta -diketonate\right)}_{3}{\left(H_{2}O\right)}_{2}+2L\;\overset{\begin{array}{c} EtOH\\ \sim 78{}^{\circ}C\\ 24h \end{array}}{\to }\;Eu{\left(\beta -diketonate\right)}_{3}{\left(L\right)}_{2}+2H_{2}O$$ (4)

The intermediates in the usual synthesis, Eu(β-diketonate)_3_(H_2_O)_2_ (step 3), are soluble in ethanol and insoluble in water. On the other hand, the formed salt (in our case KCl) is insoluble in ethanol and soluble in water. Since ethanol is the solvent used in the first step of the usual synthesis, it is important to keep filtrating the formed salt as many times as possible during the reaction. At this point, the precipitated salt is contaminated with the formed complex, and thus it luminesces. To decontaminate the salt and recover the remnants of the formed complex, the salt is washed three times with ethanol. The ethanolic filtrate is then poured into the reaction flask, which is under reflux. After 24h the reaction is completed and the solvent (ethanol) is then allowed to evaporate. When the β-diketonate DBM is used, sometimes an even more meaningful amount of the intermediate product Eu(DBM)_3_(H_2_O)_2_ precipitates with the salt while the remainder stays in solution. Then, this solid must be washed with water to remove the remaining salt, keeping only the intermediate product. What we found out is that if too much salt is left out mixed with the intermediate, when water is added, a white colloidal suspension tends to form, hindering the separation, sometimes to the point of making it impossible to be accomplished.

The second step of the usual synthesis, reaction 4, is carried out as usual, with the two coordinating water molecules being replaced by two non-ionic ligands L. The solvent is allowed to evaporate, and the remaining solid is recrystallized in a mixture of hexane 10:1 acetone. The resulting purified solid is dried out in vacuum.

Thus, having performed the synthesis of all these 6 complexes, via both the faster synthesis and via the usual route, we are now in a position to compare them, both in terms of elapsed times involved as well as in terms of overall yields.

Table 1 shows the elapsed days involved in both the usual and faster syntheses. The second step in both usual and faster syntheses lead, of course, to the same final product with the same degree of difficulty in purifying. The reason the faster synthesis is much rapid is due to its first step, which is the displacement reaction of water by the non-ionic ligand L, leading to the easy to purify intermediate EuCl_3_(L)_4_(H_2_O)_n_, a procedure which takes, on average, 3 days. Indeed, 3 days is an interval more than 4 times shorter than the one required by the first step of the usual synthesis. And the first step of the usual synthesis, which is the displacement of the chloride ions by the β-diketonate ions, leads to difficult purifications of the intermediates Eu(β-diketonate)_3_(H_2_O)_2_, a procedure which takes an average of 14 days. Therefore, in this step, the faster synthesis represents a reduction of about 80% of the elapsed time needed to obtain the corresponding pure intermediate.

**Table 1:** Elapsed days of the usual and faster synthesis, for each one of their steps as well as for their overall time.

Target Complex	Elapsed Days—Usual Synthesis			Elapsed Days—Faster Synthesis		
	Displacement reaction of		Overall	Displacement reaction of		Overall
	Cl^-^ (step 1)	H_2_O (step 2)		H_2_O (step 1)	Cl^-^ (step 2)	
Eu(DBM)_3_(TPPO)_2_	7	4	11	2	5	7
Eu(TTA)_3_(TPPO)_2_	21	4	25	2	5	7
Eu(DBM)_3_(DBSO)_2_	7	6	13	4	7	11
Eu(TTA)_3_(DBSO)_2_	21	7	28	4	7	11
Eu(DBM)_3_(PTSO)_2_	7	9	16	4	7	11
Eu(TTA)_3_(PTSO)_2_	21	8	29	4	10	14
Averages	**14**	**6**	**20**	**3**	**7**	**10**

Being fast is more powerful when combined with greater efficacy. Thus, we also examined the reaction yields of both steps of the usual and faster syntheses. Results are shown in Table 2 where, clearly, the faster synthesis displays higher yields when compared to the usual one. Indeed, in average, the usual synthesis leads to an overall yield of 42%, whereas the faster one leads to 69%. Besides, with the sole exception of step 2 of the synthesis of complex Eu(TTA)_3_(DBSO)_2_, each one of the steps of the faster syntheses lead to higher yields when compared to any of the steps of the usual synthesis.

**Table 2:** % yields of the usual and faster synthesis, for each one of their steps, together with their overall yields.

Target Complex	Usual Synthesis: % Yields			Faster Synthesis % Yields		
	Displacement reaction of		Overall	Displacement reaction of		Overall
	Cl^-^ (step 1)	H_2_O (step 2)		H_2_O (step 1)	Cl^-^ (step 2)	
Eu(DBM)_3_(TPPO)_2_	60%	51%	31%	89%	86%	77%
Eu(TTA)_3_(TPPO)_2_	73%	80%	58%	89%	88%	78%
Eu(DBM)_3_(DBSO)_2_	60%	52%	31%	92%	68%	63%
Eu(TTA)_3_(DBSO)_2_	73%	75%	55%	92%	70%	64%
Eu(DBM)_3_(PTSO)_2_	60%	53%	32%	86%	80%	69%
Eu(TTA)_3_(PTSO)_2_	73%	65%	47%	86%	75%	65%
Averages	**67%**	**63%**	**42%**	**89%**	**78%**	**69%**

We now turn to quantify the statistical significance of this finding by verifying whether the mean yield of the faster synthesis, 69%, is truly larger than the mean yield of the usual synthesis, 42%. The t-statistic for both sets of data is 4.65, which, for 5 degrees of freedom, does indicate that the mean for the faster synthesis is indeed larger than the mean for the usual synthesis within a 99.7% confidence level. This result strengthens the fact that the faster synthesis is not only speedier but that it is also overall much more effective.

### Characterization

#### Elemental Analysis and MALDI-TOF/MS

Tables 3 and 4 show the results of the elemental analysis carried out for all synthesized compounds, both intermediate and target complexes. By examining Table 4, the mean absolute error for %C of the 6 complexes synthesized by the usual route is 0.14 amu whereas the equivalent value for the same complexes synthesized by the fast route is 0.09 amu. The equivalent numbers for %H for both sets of complexes are 0.08 amu and 0.11 amu, indicating they are comparable numbers, within the usual accepted range of purity, implying they refer to the same compounds.

**Table 3:** Molecular formulas, molar masses, and calculated, c, and found, f, element mass percent values for all intermediate complexes synthesized.

Intermediate Complex	Mass (g/mol)	%C_c_	%C_f_	%H_c_	%H_f_
EuCl_3_(TPPO)_4_(H_2_O)_**3**_	1424.20	60.66	60.60	4.67	4.55
**EuC**_**72**_ **H**_**66**_ **O**_**7**_ **P**_**4**_ **Cl**_**3**_					
EuCl_3_(DBSO)_4_(H_2_O)_**4**_	1250.18	53.74	53.72	5.15	5.28
**EuC**_**56**_ **H**_**64**_ **O**_**8**_ **S**_**4**_ **Cl**_**3**_					
EuCl_3_(PTSO)_4_(H_2_O)_**4**_	1250.18	53.74	53.78	5.15	5.32
**EuC**_**56**_ **H**_**64**_ **O**_**8**_ **S**_**4**_ **Cl**_**3**_					
Eu(DBM)_3_(H_2_O)_2_	858.17	63.01	63.13	4.35	4.29
**EuC**_**45**_ **H**_**37**_ **O**_**8**_					
Eu(TTA)_3_(H_2_O)_2_	851.91	33.85	33.68	1.89	1.74
**EuC**_**24**_ **H**_**16**_ **O**_**8**_ **S**_**3**_ **F**_**9**_					

**Table 4:** Molecular formulas, molar masses, and calculated, c, and found, f, element mass percent values for all target complexes synthesized.

Target Complex	Mass (g/mol)	Usual Synthesis:				Faster Synthesis:			
		%C_c_	%C_f_	%H_c_	%H_f_	%C_c_	%C_f_	%H_c_	%H_f_
Eu(DBM)_3_(TPPO)_2_	1378.32	70.59	70.72	4.61	4.64	70.59	70.51	4.61	4.77
**EuC**_**81**_ **H**_**63**_ **O**_**8**_ **P**_**2**_									
Eu(TTA)_3_(TPPO)_2_	1372.06	52.52	52.42	3.09	3.06	52.52	52.70	3.09	3.05
**EuC**_**60**_ **H**_**42**_ **O**_**8**_ **P**_**2**_ **S**_**3**_ **F**_**9**_									
Eu(DBM)_3_(DBSO)_2_	1282.30	68.37	68.56	4.79	4.61	68.37	68.46	4.79	4.91
**EuC**_**73**_ **H**_**61**_ **O**_**8**_ **S**_**2**_									
Eu(TTA)_3_(DBSO)_2_	1276.04	48.94	48.83	3.16	3.12	48.94	48.85	3.16	3.03
**EuC**_**52**_ **H**_**40**_ **O**_**8**_ **S**_**5**_ **F**_**9**_									
Eu(DBM)_3_(PTSO)_2_	1282.30	68.37	68.41	4.79	4.90	68.37	68.34	4.79	4.94
**EuC**_**73**_ **H**_**61**_ **O**_**8**_ **S**_**2**_									
Eu(TTA)_3_(PTSO)_2_	1276.04	48.94	48.65	3.16	3.27	48.94	48.86	3.16	3.19
**EuC**_**52**_ **H**_**40**_ **O**_**8**_ **S**_**5**_ **F**_**9**_									

Table 5 shows MALDI-TOF data, together with elemental analysis results for the novel intermediates obtained by the faster synthesis of europium β-diketonate complexes advanced in this article.

**Table 5:** Mass spectrometry (MALDI-TOF) data for the novel intermediate europium complexes. Calculated values are in parenthesis.

Novel Intermediate complex	[M+H]^+^ (m/z)
EuCl_3_(TPPO)_4_(H_2_O)_**3**_	1425.22
**(EuC**_**72**_ **H**_**66**_ **O**_**7**_ **P**_**4**_ **Cl**_**3**_ **)**	(1425.20)
EuCl_3_(DBSO)_4_(H_2_O)_**4**_	1251.15
**(EuC**_**56**_ **H**_**64**_ **O**_**8**_ **S**_**4**_ **Cl**_**3**_ **)**	(1251.18)
EuCl_3_(PTSO)_4_(H_2_O)_**4**_	1251.16
**(EuC**_**56**_ **H**_**64**_ **O**_**8**_ **S**_**4**_ **Cl**_**3**_ **)**	(1251.18)

#### IR Spectroscopy

Infrared spectra are presented in the Supporting Information. By examining the spectra of all complexes, the stretchings of the C = O, S = O, P = O groups coordinated to the europium ion, can be easily identified. Likewise, the C-H stretchings corresponding to the CH_3_ group of PTSO, and to the CH_2_ group of DBSO, can also be easily observed. For example, consider the spectrum of complex Eu(DBM)_3_(DBSO)_2_, prepared by the faster synthesis. The following stretching signals appear at: ν (= C-H) 3083 cm^-1^–3032cm^-1^, ν CH_2_ 2960 cm^-1^–2913 cm^-1^, ν C = O 1599 cm^-1^ and ν S = O 1028 cm^-1^. For the same complex obtained by the usual synthesis, the corresponding values are: ν (= C-H) 3055–3018 cm^-1^, ν CH_2_ 2956 cm^-1^, ν C = O 1594 cm^-1^, ν S = O 1022 cm^-1^. For the complex Eu(DBM)_3_(PTSO)_2_, prepared by the faster synthesis, the signals observed are: ν (= C-H) 3059 cm^-1^–3027 cm^-1^, ν CH_3_ 2947 cm^-1^–2857 cm^-1^, ν C = O 1599 cm^-1^, ν S = O 1018 cm^-1^. For the same complex obtained by the usual synthesis, the signals observed are: ν (= C-H) 3060–3013 cm^-1^, ν CH_3_ 2980–2861 cm^-1^, ν C = O 1595 cm^-1^, ν S = O 1012 cm^-1^. For the complex Eu(DBM)_3_(TPPO)_2_, prepared by the faster synthesis, we observed the signals are: ν (= C-H) 3067 cm^-1^–3020 cm^-1^, ν C = O 1597 cm^-1^ and ν P = O 1074 cm^-1^, whereas for the same complex obtained by the usual synthesis, these values are: ν (= C-H) 3082–3027 cm^-1^, ν C = O 1599 cm^-1^ and ν P = O 1070 cm^-1^.

#### ^1^H NMR Spectroscopy

^1^H NMR spectra of all free ligands and complexes synthesized in this article can be found in the Supporting Information. Signals corresponding to hydrogen nuclei of the complexes appear broadened. Nevertheless, signals from: aromatic hydrogens; CH of β-diketonates; CH_2_ of DBSO; and CH_3_ of PTSO, can be easily identified. In order to rule out the possibility that the ^1^H NMR charts could contain reflection signals of protons outside of the usual 0 ppm to 20 ppm range, we measured the ^1^H NMR spectra of the novel intermediate complexes from -200 ppm to +200 ppm. No signals were detected for any of the novel complexes outside of the usual range. The Supporting Information contains these wide range spectra in S1 File. Thus, consider complex Eu(DBM)_3_(PTSO)_2_, prepared by the faster synthesis. The corresponding chemical shift signals are: for the methine hydrogen, δ (s,CH) 16.79 ppm; for the CH_3_ group of PTSO δ (s,CH_3_) 2.47 ppm; and for the aromatic hydrogens, we identified signals at δ (m,C_6_H_5_) δ 7.96 ppm—δ 7.24 ppm. On the other hand, presence of DBSO in the structure of europium complexes can be identified by the signal corresponding to the hydrogen nuclei of the CH_2_ group. For example, in the spectra of the intermediate complex EuCl_3_(DBSO)_4_(H_2_O)_4_, and of the target complex Eu(DBM)_3_(DBSO)_2_, both prepared by the faster synthesis, the corresponding chemical shifts of the CH_2_ group are: δ 3.90 ppm and δ 4.72 ppm, respectively.

Table 6 shows the ^1^H chemical shifts, δ, of the methine group of the β-diketonate ligands coordinated to the europium ion for all synthesized complexes. Clearly, the average chemical shifts of the methine hydrogen of the DBM ligands are unshielded to an average of δ 16.85 ppm. However, the average chemical shifts of the methine hydrogen for TTA is δ 10.24 ppm. Apparently, this is due to the electron withdrawing effect of the single CF_3_ group of TTA. We propose that this CF_3_ group, being electronegative, pulls the electrons from the carbonyl oxygens—more distinctly so from its closest carbonyl oxygen—weakening the coordination of the TTA ligand to the europium ion, leading to a shielding on the methine hydrogen. On the other hand, DBM, which contains two identical aromatic phenyl groups, is more capable of balancing the coordinating bond strengths of its two carbonyl oxygens to the europium ion in a more efficient manner, leading to a more pronounced unshielding of the methine hydrogen.

**Table 6:** Chemical shift values of the hydrogen nucleus (δ) in the CH group of the β-diketonate ligands obtained by faster synthesis.

Complex	δ (ppm) CH group
Eu(DBM)_3_(H_2_O)_2_	16.66
Eu(DBM)_3_(TPPO)_2_	17.15
Eu(DBM)_3_(DBSO)_2_	16.80
Eu(DBM)_3_(PTSO)_2_	16.79
Eu(TTA)_3_(H_2_O)_2_	10.79
Eu(TTA)_3_(TPPO)_2_	9.09
Eu(TTA)_3_(DBSO)_2_	11.64
Eu(TTA)_3_(PTSO)_2_	9.43

#### ^19^F NMR Spectroscopy

The presence of the TTA ligand in the structure of the complexes can be confirmed by ^19^F NMR spectroscopy. For example, while the δ ^19^F in the TTA free ligand is -75.80 ppm, for the three TTA containing complexes synthesized in this article, the observed signals are: δ -79.97 ppm for Eu(TTA)_3_(TPPO)_2_, δ -80.38 ppm for Eu(TTA)_3_(DBSO)_2_, and δ -80.51 ppm for Eu(TTA)_3_(PTSO)_2_. Since the compounds are pure, as indicated by elemental analysis, the very few other low intensity and broadened signals in the ^19^F NMR spectra may perhaps be a result of the presence of a few coordination isomers.

#### ^31^P NMR Spectroscopy

Likewise, the presence of the TPPO ligand in the structure of the europium complex can be easily confirmed by ^31^P NMR spectroscopy. Indeed, while the ^31^P NMR spectrum of the TPPO free ligand shows a single signal at δ 28 ppm, in the intermediate complex Eu(TPPO)_4_Cl_3_(H_2_O)_3_, the phosphorus signal appears at δ 30 ppm, albeit broadened by the paramagnetism of the europium ion, indicating complexation. Similarly, for complex Eu(DBM)_3_(TPPO)_2_, the phosphorus chemical shift also appears in the same region at δ 25 ppm. On the other hand, in complexes which contain TTA as ionic ligands, the phosphorus chemical shift is acutely displaced to δ-66 ppm, a shift of the order of ~100 ppm, indicating that the phosphorus nuclei are strongly affected by TTA, probably due to the presence of the strongly electron withdrawing group CF_3_. Recently, similar effects were observed for complexes Eu(BTFA)_3_(TPPO)_2_, Eu(BTFA)_3_(DBSO,TPPO), Eu(BTFA)_3_(PTSO,TPPO), Eu(TTA)_3_(DBSO,TPPO) and Eu(TTA)_3_(PTSO,TPPO) which also displayed ^31^P chemical shifts of δ -53 ppm, δ -74 ppm, δ -77 ppm, δ -74 ppm and δ -75 ppm, respectively [29].

## Conclusions

The difficulty involved in the purification of the synthetic intermediates Eu(β-diketonate)_3_(H_2_O)_2_, which may take up to a few weeks, prompted us to devise a new strategy for the syntheses of highly luminescent europium complexes of the general formula Eu(β-diketonate)_3_(L)_2_, where L stands for a nonionic ligand.

Experimental proof illustrating the presumed all-encompassing efficacy of the new strategy was provided by a synthetic combinatorial approach. All Eu(β-diketonate)_3_(L)_2_ complexes made up by any of the β-diketonate ligands, DBM or TTA, and by any of the non-ionic ligands DBSO, PTSO, or TPPO, were synthesized both by the usual and by the faster synthesis advanced in this article.

Indeed, by simply switching the order in which ionic and non-ionic ligands are usually added to the coordination polyhedron of the europium ion, that is, by first coordinating the non-ionic ligands, and by only subsequently coordinating the ionic ligands, we were able to halve the average elapsed time for the preparation of these highly luminescent complexes from 20 days to 10 days, while boosting the average overall yield of the syntheses from 42% to 69%.

## Supporting Information

S1 File Characterization data: MALDI-TOF/MS, Infrared, and ^1^H, ^19^F, and ^31^P NMR spectra. (PDF)
