# Supplementary material for: Faster Synthesis of Beta-Diketonate Ternary Europium Complexes: Elapsed Times & Reaction Yields
Source: PLoS One. 2015 Dec 28;10(12):e0143998. doi: 10.1371/journal.pone.0143998 (PMC4692388; doi:10.1371/journal.pone.0143998)
Supplement: S1 File — (PDF) [file pone.0143998.s001.pdf]

Supporting Information

Faster Synthesis of Beta-Diketonate Ternary Europium Complexes: Elapsed Times & Reaction Yields

Nathalia B. D. Lima, Anderson I. S. Silva, Gerson P. C. Jr,

Simone M. C. Gonçalves* and Alfredo M. Simas*

simone@ufpe.br, simas@ufpe.br

Summary

Figures....2

Table....3

Characterization....4

MALDI-TOF Mass Spectrometry....4

Infrared Spectra....6

NMR Spectra....17

^{1}H NMR Spectra....17

^{19}F NMR Spectra....31

^{31}P NMR Spectra....35

Summary of characterizations....38

Figures

S1 Fig. MALDI-TOF/MS spectrum of the complex $EuCl_3(TPPO)_4(H_2O)_3$. 4
S2 Fig. MALDI-TOF/MS spectrum of the complex $EuCl_3(DBSO)_4(H_2O)_4$. 5
S3 Fig. MALDI-TOF/MS spectrum of the complex $EuCl_3(PTSO)_4(H_2O)_4$. 5
S4 Fig. Infrared spectrum DBMH free ligand. 6
S5 Fig. Infrared spectrum TTAH free ligand. 6
S6 Fig. Infrared spectrum TPPO free ligand. 7
S7 Fig. Infrared spectrum DBSO free ligand. 7
S8 Fig. Infrared spectrum PTSO free ligand. 8
S9 Fig. Infrared spectrum of $EuCl_3(TPPO)_4(H_2O)_3$. 8
S10 Fig. Infrared spectrum of $EuCl_3(DBSO)_4(H_2O)_4$. 9
S11 Fig. Infrared spectrum of $EuCl_3(PTSO)_4(H_2O)_4$. 9
S12 Fig. Infrared spectrum of $Eu(TTA)_3(H_2O)_2$. 10
S13 Fig. Infrared spectrum of $Eu(DBM)_3(H_2O)_2$. 10
S14 Fig. Infrared spectrum of $Eu(DBM)_3(TPPO)_2$ by usual synthesis route. 11
S15 Fig. Infrared spectrum of $Eu(DBM)_3(TPPO)_2$ by faster synthesis route. 11
S16 Fig. Infrared spectrum of $Eu(TTA)_3(TPPO)_2$ by usual synthesis route. 12
S17 Fig. Infrared spectrum of $Eu(TTA)_3(TPPO)_2$ by faster synthesis route. 12
S18 Fig. Infrared spectrum of $Eu(DBM)_3(DBSO)_2$ by usual synthesis route. 13
S19 Fig. Infrared spectrum of $Eu(DBM)_3(DBSO)_2$ by faster synthesis route. 13
S20 Fig. Infrared spectrum of $Eu(TTA)_3(DBSO)_2$ by usual synthesis route. 14
S21 Fig. Infrared spectrum of $Eu(TTA)_3(DBSO)_2$ by faster synthesis route. 14
S22 Fig. Infrared spectrum of $Eu(DBM)_3(PTSO)_2$ by usual synthesis route. 15
S23 Fig. Infrared spectrum of $Eu(DBM)_3(PTSO)_2$ by faster synthesis route. 15
S24 Fig. Infrared spectrum of $Eu(TTA)_3(PTSO)_2$ by usual synthesis route. 16
S25 Fig. Infrared spectrum of $Eu(TTA)_3(PTSO)_2$ by faster synthesis route. 16
S26 Fig. ^{1}H NMR spectrum of DBMH free ligand. 17
S27 Fig. ^{1}H NMR spectrum of TTAH free ligand. 17
S28 Fig. ^{1}H NMR spectrum of TPPO free ligand. 18
S29 Fig. ^{1}H NMR spectrum of DBSO free ligand. 18
S30 Fig. ^{1}H NMR spectrum of PTSO free ligand. 19
S31 Fig. ^{1}H NMR spectrum of $EuCl_3(TPPO)_4(H_2O)_3$, measured from 0 to 200 ppm to rule out the possibility that the charts could contain reflection signals of protons outside of the usual 0-20ppm range. 19
S32 Fig. ^{1}H NMR spectrum of $EuCl_3(TPPO)_4(H_2O)_3$, , measured from 20 to -200 ppm to rule out the possibility that the charts could contain reflection signals of protons outside of the usual 0-20ppm range. 20
S33 Fig. ^{1}H NMR spectrum of $EuCl_3(DBSO)_4(H_2O)_4$, measured from 0 to 200 ppm to rule out the possibility that the charts could contain reflection signals of protons outside of the usual 0-20ppm range. 20
S34 Fig. ^{1}H NMR spectrum of $EuCl_3(DBSO)_4(H_2O)_4$, , measured from 20 to -200 ppm to rule out the possibility that the charts could contain reflection signals of protons outside of the usual 0-20ppm range. 21

S35 Fig. ^{1}H NMR spectrum of $EuCl_3(PTSO)_4(H_2O)_4$, measured from 0 to 200 ppm to rule out the possibility that the charts could contain reflection signals of protons outside of the usual 0-20ppm range........21
S36 Fig. ^{1}H NMR spectrum of $EuCl_3(PTSO)_4(H_2O)_4$, measured from 20 to -200 ppm to rule out the possibility that the charts could contain reflection signals of protons outside of the usual 0-20ppm range........22
S37 Fig. ^{1}H NMR spectrum of $EuCl_3(TPPO)_4(H_2O)_3$........22
S38 Fig. ^{1}H NMR spectrum of $EuCl_3(DBSO)_4(H_2O)_4$........23
S39 Fig. ^{1}H NMR spectrum of $EuCl_3(PTSO)_4(H_2O)_4$........23
S40 Fig. ^{1}H NMR spectrum of $Eu(TTA)_3(H_2O)_2$........24
S41 Fig. ^{1}H NMR spectrum of $Eu(DBM)_3(H_2O)_2$........24
S42 Fig. ^{1}H NMR spectrum of $Eu(DBM)_3(TPPO)_2$ of the usual synthesis........25
S43 Fig. ^{1}H NMR spectrum of $Eu(DBM)_3(TPPO)_2$ of the faster synthesis........25
S44 Fig. ^{1}H NMR spectrum of $Eu(TTA)_3(TPPO)_2$ of the usual synthesis........26
S45 Fig. ^{1}H NMR spectrum of $Eu(TTA)_3(TPPO)_2$ of the faster synthesis........26
S46 Fig. ^{1}H NMR spectrum of $Eu(DBM)_3(DBSO)_2$ of the usual synthesis........27
S47 Fig. ^{1}H NMR spectrum of $Eu(DBM)_3(DBSO)_2$ of the faster synthesis........27
S48 Fig. ^{1}H NMR spectrum of $Eu(TTA)_3(DBSO)_2$ of the usual synthesis........28
S49 Fig. ^{1}H NMR spectrum of $Eu(TTA)_3(DBSO)_2$ of the faster synthesis........28
S50 Fig. ^{1}H NMR spectrum of $Eu(DBM)_3(PTSO)_2$ of the usual synthesis........29
S51 Fig. ^{1}H NMR spectrum of $Eu(DBM)_3(PTSO)_2$ of the faster synthesis........29
S52 Fig. ^{1}H NMR spectrum of $Eu(TTA)_3(PTSO)_2$ of the usual synthesis........30
S53 Fig. ^{1}H NMR spectrum of $Eu(TTA)_3(PTSO)_2$ of the faster synthesis........30
S54 Fig. ^{19}F NMR spectrum of TTA free ligand........31
S55 Fig. ^{19}F NMR spectrum of $Eu(TTA)_3(H_2O)_2$........31
S56 Fig. ^{19}F NMR spectrum of $Eu(TTA)_3(TPPO)_2$ of the usual synthesis........32
S57 Fig. ^{19}F NMR spectrum of $Eu(TTA)_3(TPPO)_2$ of the faster synthesis........32
S58 Fig. ^{19}F NMR spectrum of $Eu(TTA)_3(DBSO)_2$ of the usual synthesis........33
S59 Fig. ^{19}F NMR spectrum of $Eu(TTA)_3(DBSO)_2$ of the faster synthesis........33
S60 Fig. ^{19}F NMR spectrum of $Eu(TTA)_3(PTSO)_2$ of the usual synthesis........34
S61 Fig. ^{19}F NMR spectrum of $Eu(TTA)_3(PTSO)_2$ of the faster synthesis........34
S62 Fig. ^{31}P NMR spectrum of TPPO free ligand........35
S63 Fig. ^{31}P NMR spectrum of $EuCl_3(TPPO)_4(H_2O)_3$........35
S64 Fig. ^{31}P NMR spectrum of $Eu(DBM)_3(TPPO)_2$ of the usual synthesis........36
S65 Fig. ^{31}P NMR spectrum of $Eu(DBM)_3(TPPO)_2$ of the faster synthesis........36
S66 Fig. ^{31}P NMR spectrum of $Eu(TTA)_3(TPPO)_2$ of the usual synthesis........37
S67 Fig. ^{31}P NMR spectrum of $Eu(TTA)_3(TPPO)_2$ of the faster synthesis........37

Table

S1 Table. Summary of characterization data for all complexes synthesized in this article........38

Characterization

MALDI-TOF Mass Spectrometry

The matrix-assisted laser desorption-ionization/time-of-flight mass, MALDI-TOF, mass spectra were acquired on an Autoflex 3 Smart Beam Vertical spectrometer by BruckerDaltonics, USA. The system utilizes a 100 Hz pulsed nitrogen laser Nd:Yag emitting at 355 nm. The spectra were acquired in positive reflection mode between m/z 500 and 4880. All spectra represent the average of 1000 single laser shots. The laser intensity was kept sufficiently low to prevent degradation of the complexes and to obtain a good signal-to-noise ratio of the analyte. The equipment was calibrated with the peptide calibration standard (Bruker), and the spectra were acquired with FlexControl software (Bruker Daltonics, version 3.0).

The spectra of the novel intermediate complexes are shown in S1–S3 Figs below:

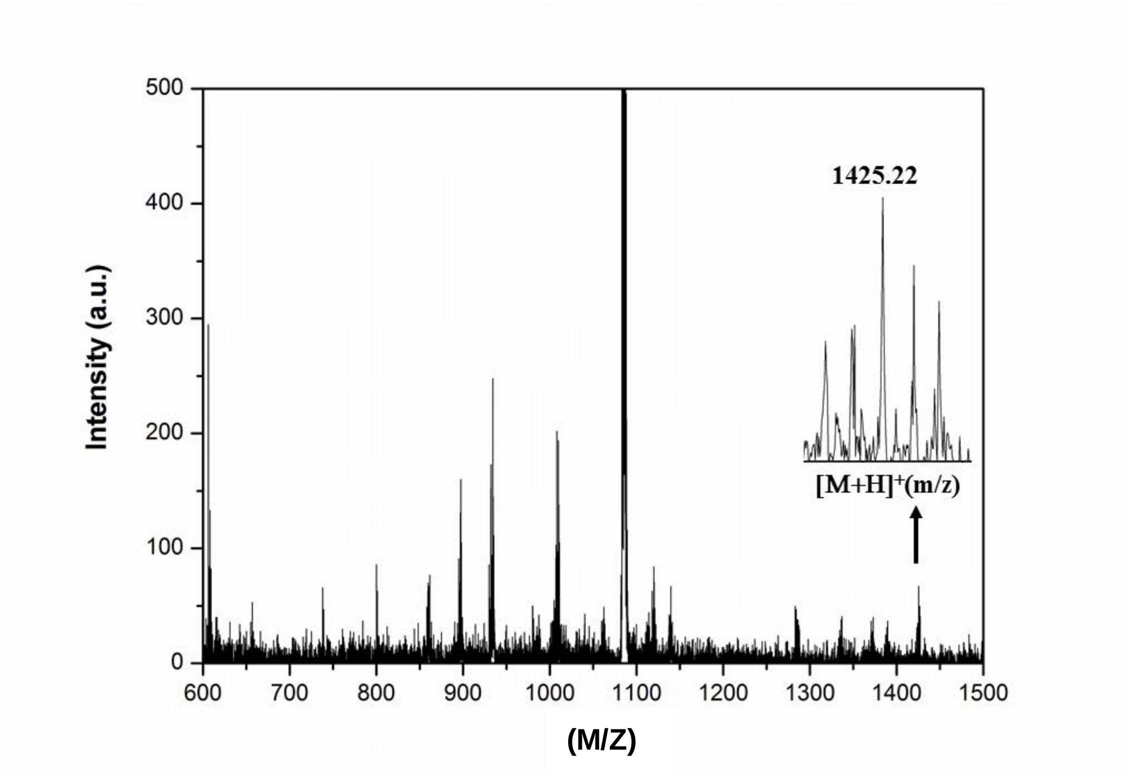

S1 Fig. MALDI-TOF/MS spectrum of the complex $EuCl_3(TPPO)_4(H_2O)_3$.

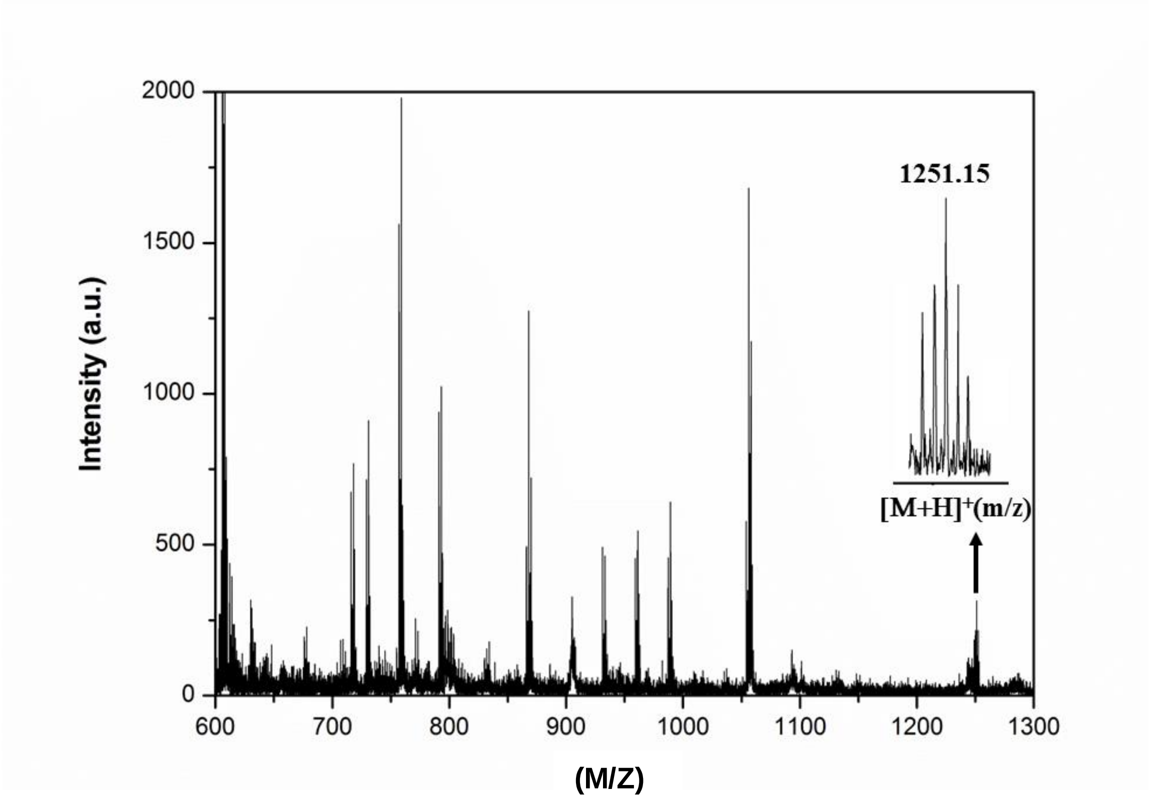

S2 Fig. MALDI-TOF/MS spectrum of the complex $EuCl_3(DBSO)_4(H_2O)_4$.

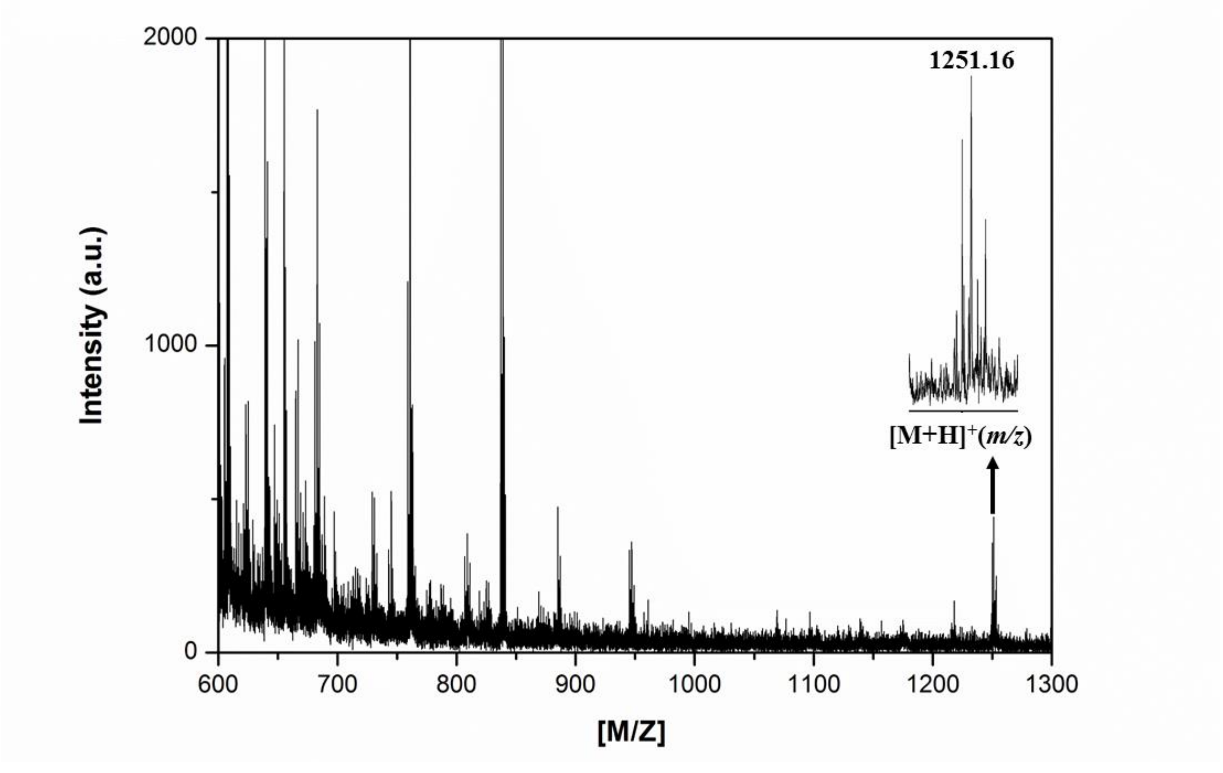

S3 Fig. MALDI-TOF/MS spectrum of the complex $EuCl_3(PTSO)_4(H_2O)_4$.

Infrared Spectra

Samples of the complexes were prepared as KBr disks, and the spectra were measured in a Bruker model IFS 66 spectrophotometer (4000 cm^{-1} - 400 cm^{-1}). S4-S25 Figs show the obtained infrared spectra for all free ligands and synthesized complexes.

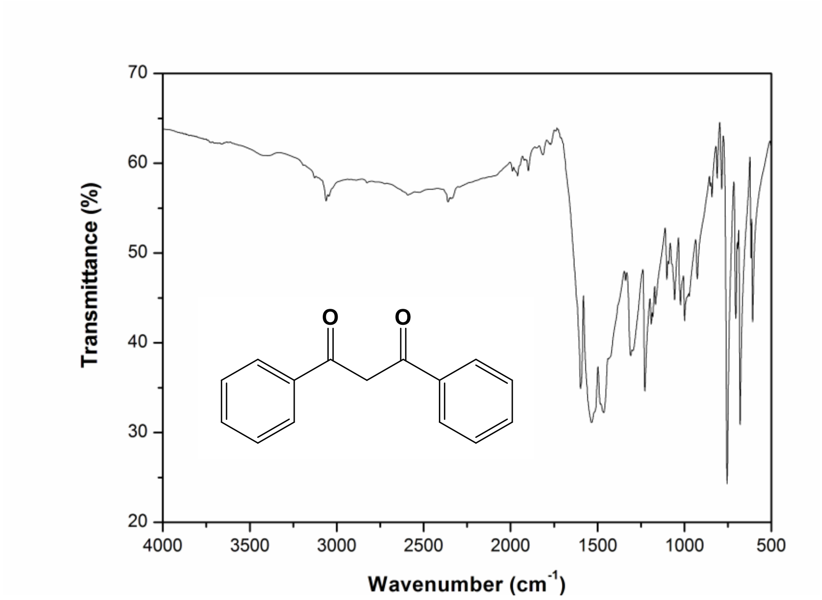

DBMH free ligand (KBr): ν=C-H 3060 cm^{-1}– 3038 cm^{-1}, νC=O 1599 cm^{-1}.

S4 Fig. Infrared spectrum DBMH free ligand.

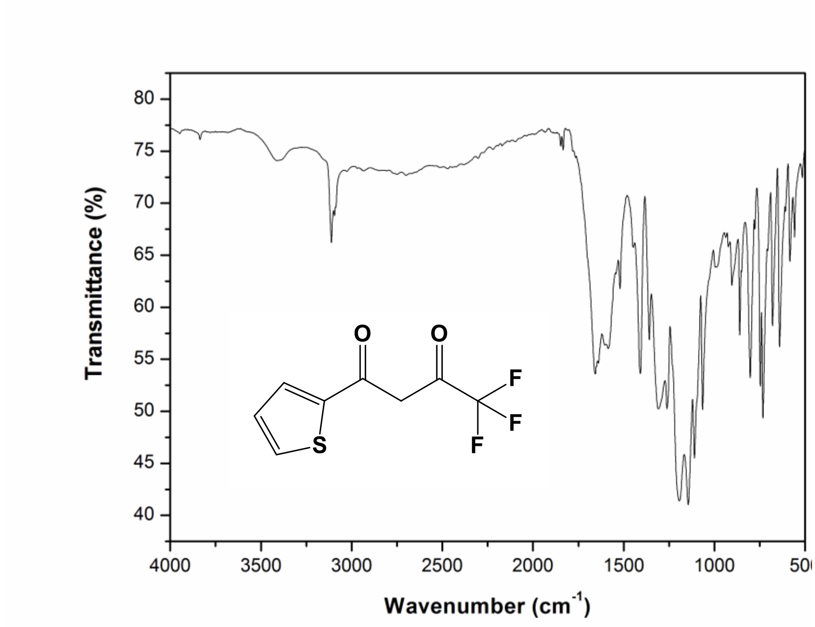

TTAH free ligand (KBr): ν=C-H 3107 cm^{-1}– 3087cm^{-1}, νC=O 1655 cm^{-1}.

S5 Fig. Infrared spectrum TTAH free ligand.

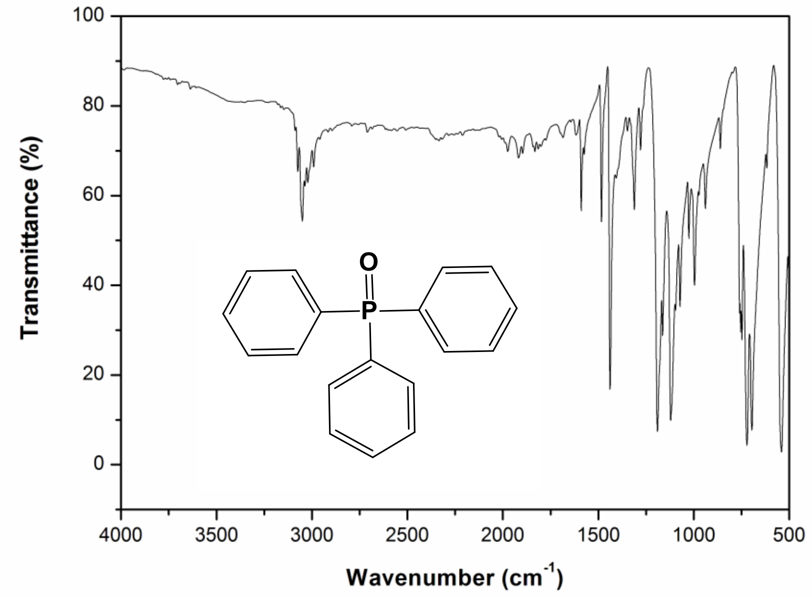

TPPO free ligand (KBr): ν=C-H 3091 cm^{-1} – 3000 cm^{-1}, νP=O 1118 cm^{-1}.

S6 Fig. Infrared spectrum TPPO free ligand.

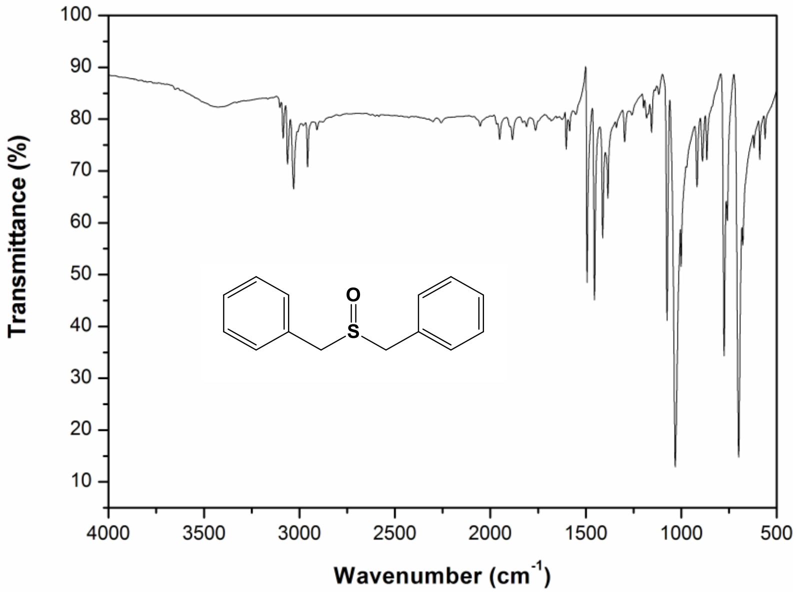

DBSO free ligand (KBr): ν=C-H 3102 cm^{-1}– 3036 cm^{-1}, ν CH_2 2960 cm^{-1}– 2913cm^{-1}, νS=O 1032 cm^{-1}.

S7 Fig. Infrared spectrum DBSO free ligand.

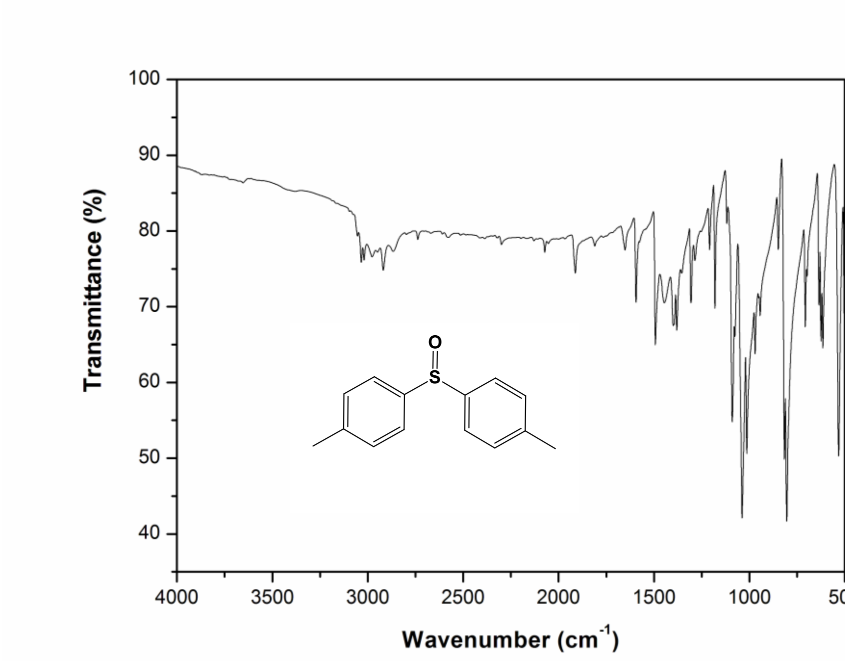

PTSO free ligand (KBr): ν=C-H 3051 cm^{-1} – 3017 cm^{-1}, ν CH_3 2975 cm^{-1} – 2862cm^{-1}, νS=O 1037 cm^{-1}.

S8 Fig. Infrared spectrum PTSO free ligand.

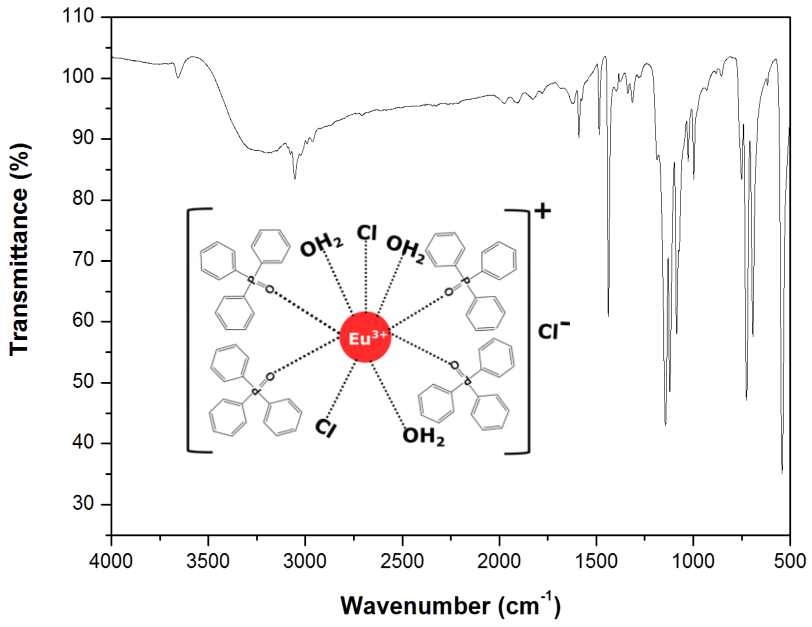

$EuCl_3(TPPO)_4(H_2O)_3$ (KBr): νO-H 3661 cm^{-1},ν=C-H 3092 cm^{-1}– 3017 cm^{-1}, νP=O 1088 cm^{-1}.

S9 Fig. Infrared spectrum of $EuCl_3(TPPO)_4(H_2O)_3$.

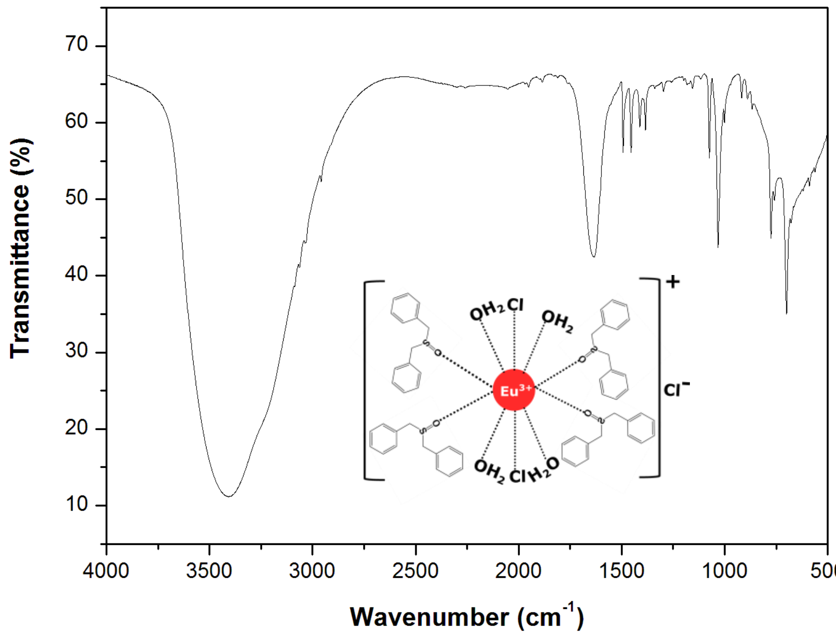

$EuCl_3(DBSO)_4(H_2O)_4$ (KBr): νO-H 3407 cm^{-1}, νS=O 1031 cm^{-1}.

S10 Fig. Infrared spectrum of $EuCl_3(DBSO)_4(H_2O)_4$.

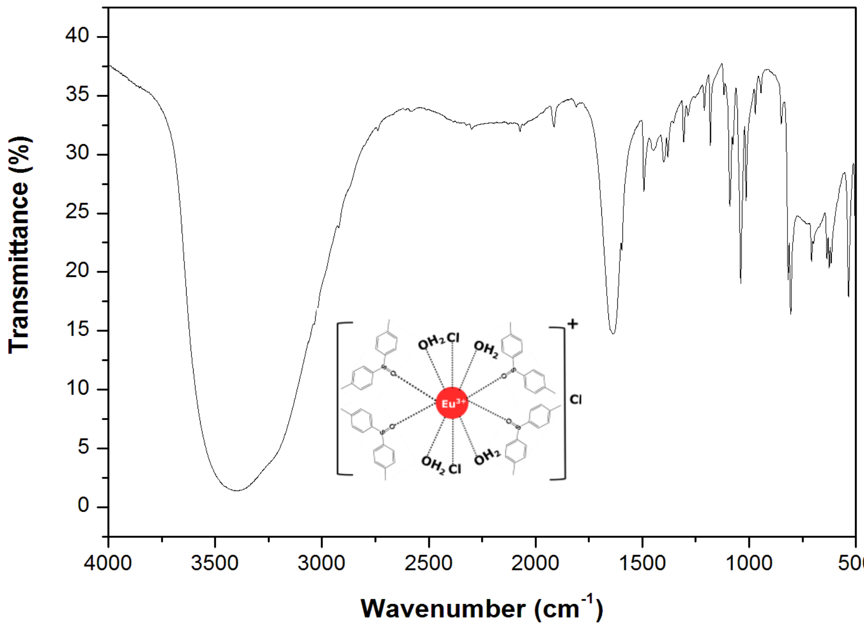

$EuCl_3(PTSO)_4(H_2O)_4$ (KBr): νO-H 3704 cm^{-1}, νS=O 1036 cm^{-1}.

S11 Fig. Infrared spectrum of $EuCl_3(PTSO)_4(H_2O)_4$.

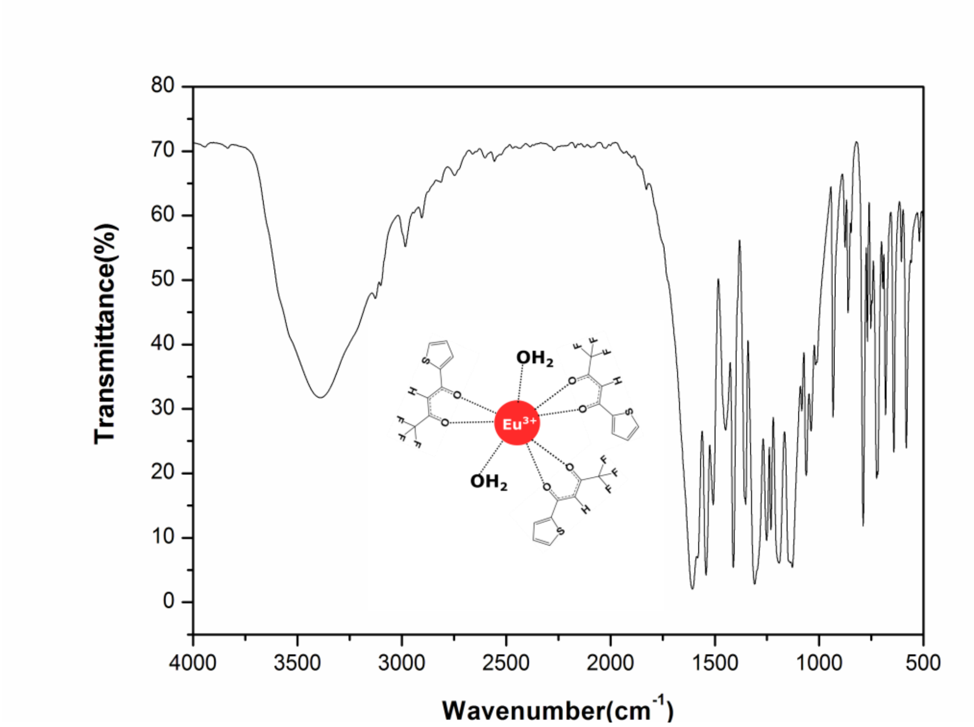

Eu(TTA)$_3$(H$_2$O)$_2$ (KBr): ν=O-H 3394 cm^{-1}, νC=O 1617 cm^{-1}.

S12 Fig. Infrared spectrum of Eu(TTA)$_3$(H$_2$O)$_2$.

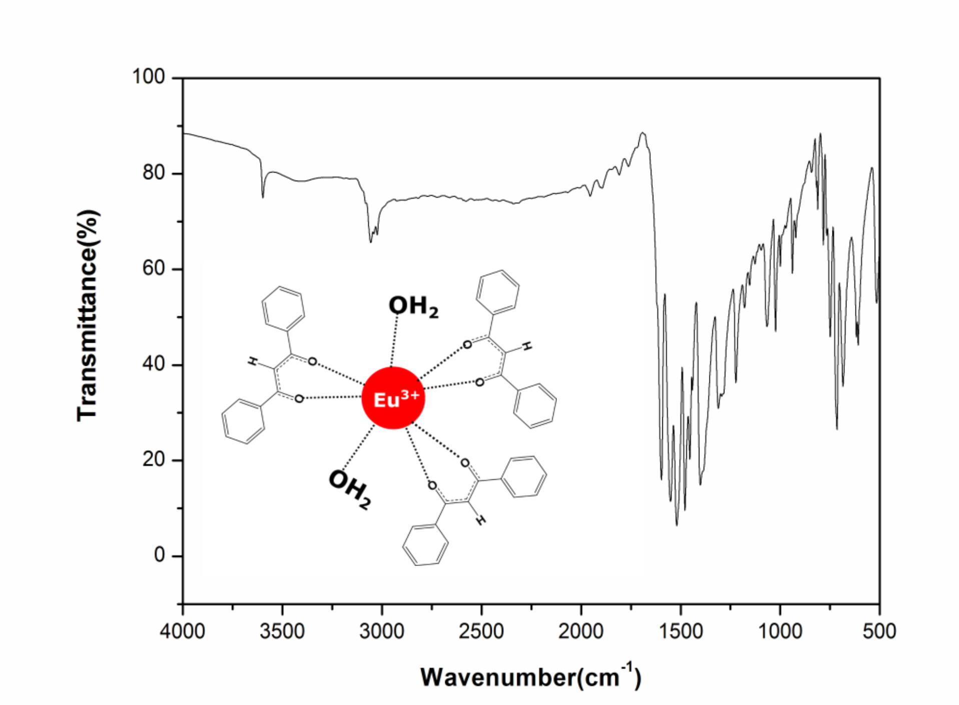

Eu(DBM)$_3$(H$_2$O)$_2$(KBr): ν O-H 3604,ν (=C-H) 3060 - 3015cm^{-1}, ν C=O 1596 cm^{-1}.

S13 Fig. Infrared spectrum of Eu(DBM)$_3$(H$_2$O)$_2$.

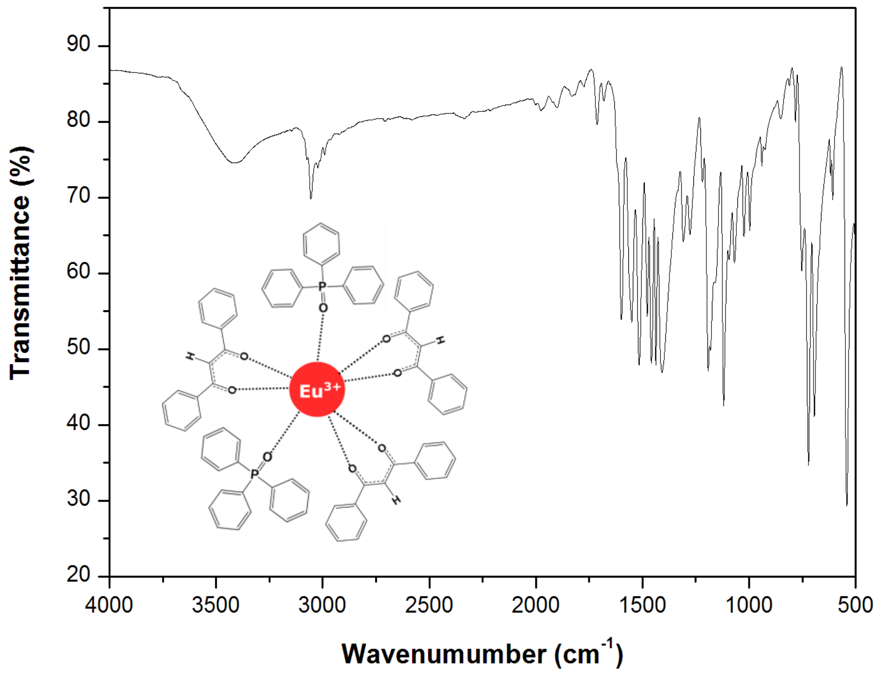

Eu(DBM)$_3$(TPPO)$_2$ (KBr): ν(=C-H) 3082-3027 cm^{-1}, νC=O 1599 cm^{-1}, νP=O 1070 cm^{-1}.

S14 Fig. Infrared spectrum of Eu(DBM)$_3$(TPPO)$_2$ by usual synthesis route.

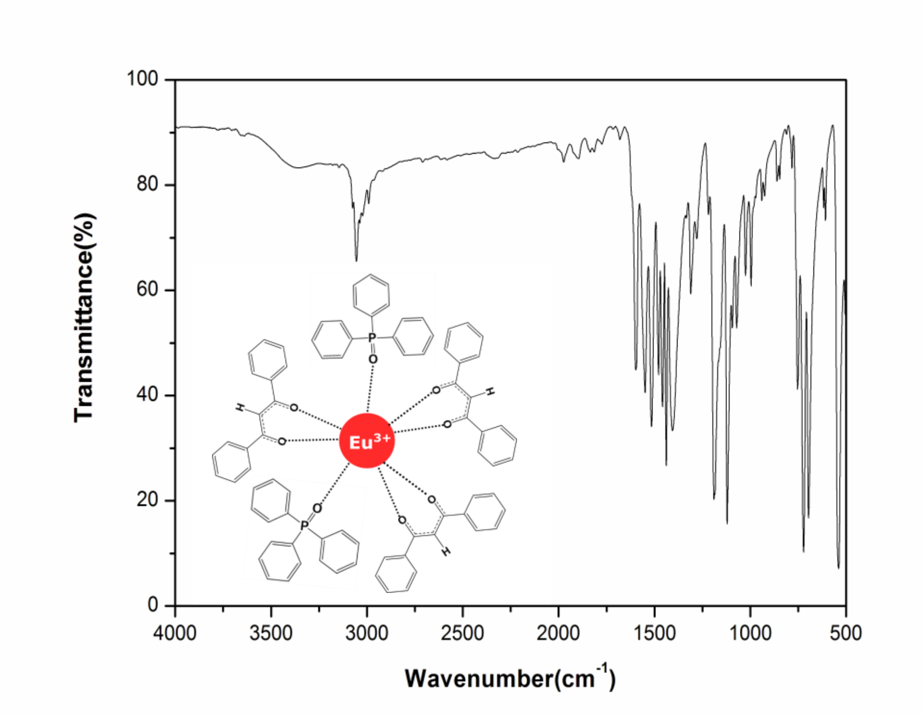

Eu(DBM)$_3$(TPPO)$_2$ (KBr): ν=C-H 3067 cm^{-1} – 3020 cm^{-1} , ν C=O 1597 cm^{-1}, νP=O 1074 cm^{-1}.

S15 Fig. Infrared spectrum of Eu(DBM)$_3$(TPPO)$_2$ by faster synthesis route.

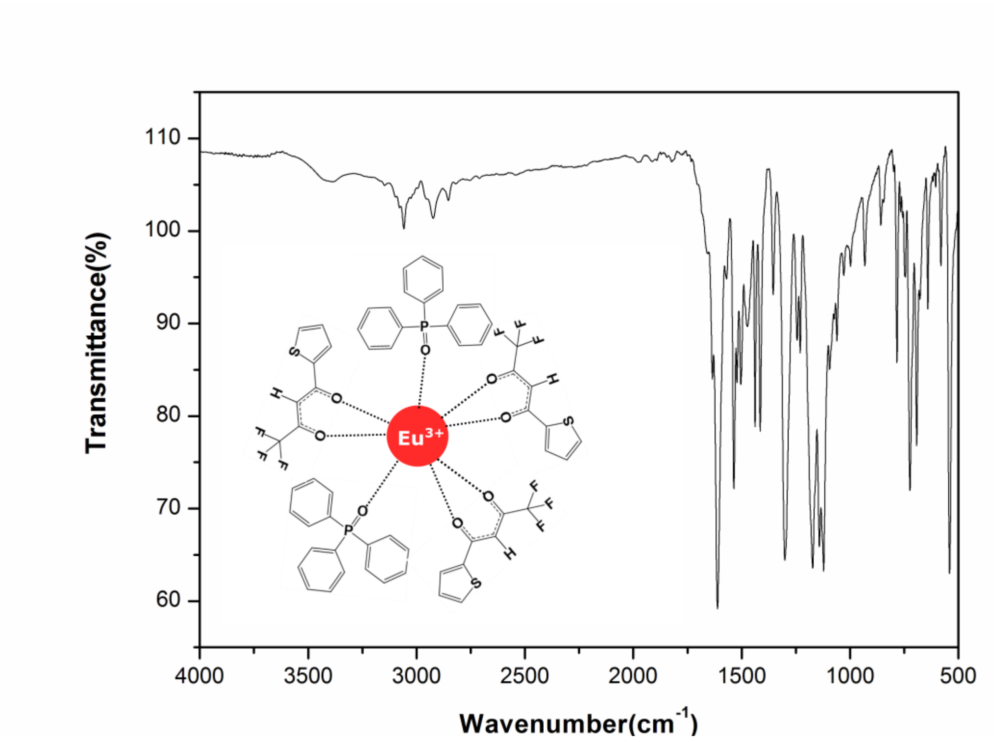

Eu(TTA)$_3$(TPPO)$_2$ (KBr): ν=C-H 3102 cm^{-1} – 3032 cm^{-1} , ν C=O 1608 cm^{-1}, νP=O 1065 cm^{-1}.

S16 Fig. Infrared spectrum of Eu(TTA)$_3$(TPPO)$_2$ by usual synthesis route.

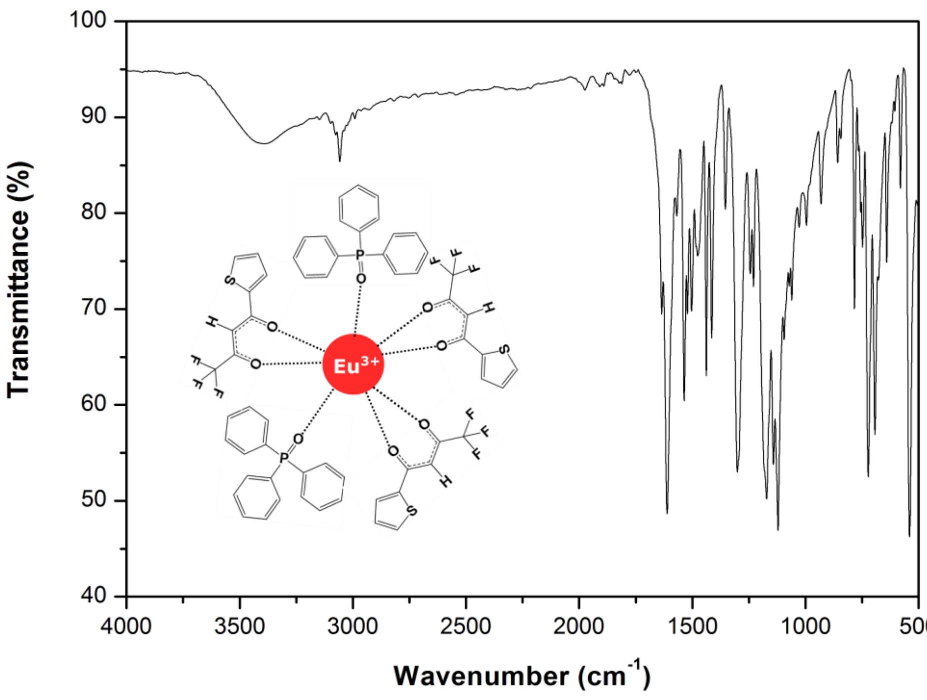

Eu(TTA)3(TPPO)$_2$ (KBr): ν=C-H 3104 cm^{-1} – 3030 cm^{-1} , ν C=O 1607 cm^{-1}, νP=O 1060 cm^{-1}.

S17 Fig. Infrared spectrum of Eu(TTA)$_3$(TPPO)$_2$ by faster synthesis route.

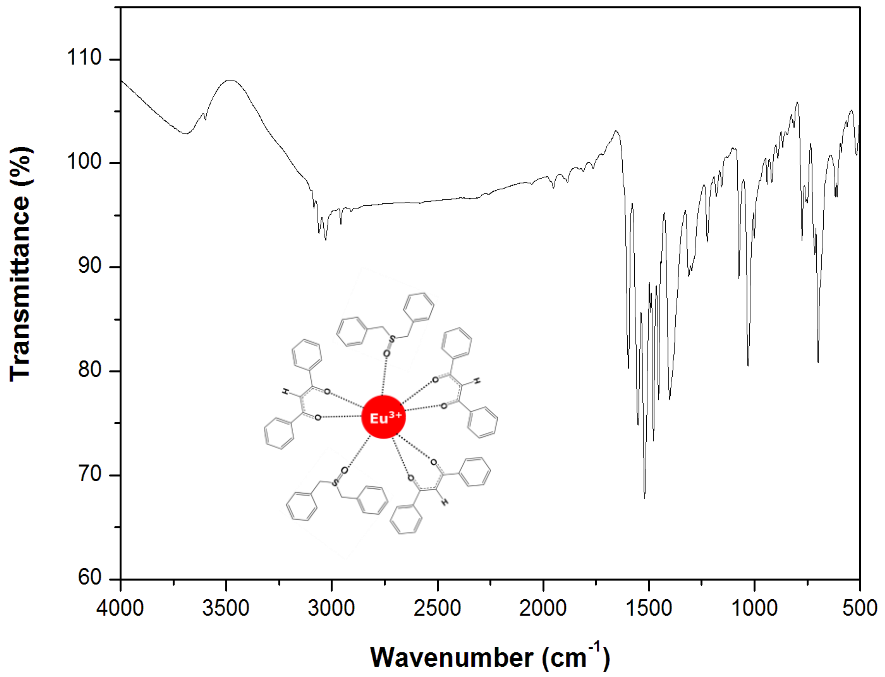

Eu(DBM)$_3$(DBSO)$_2$ T(KBr): ν(=C-H) 3055 -3018cm^{-1}, ν CH_2 2956 cm^{-1}, ν C=O 1594 cm^{-1}, νS=O 1022 cm^{-1}.

S18 Fig. Infrared spectrum of Eu(DBM)$_3$(DBSO)$_2$ by usual synthesis route.

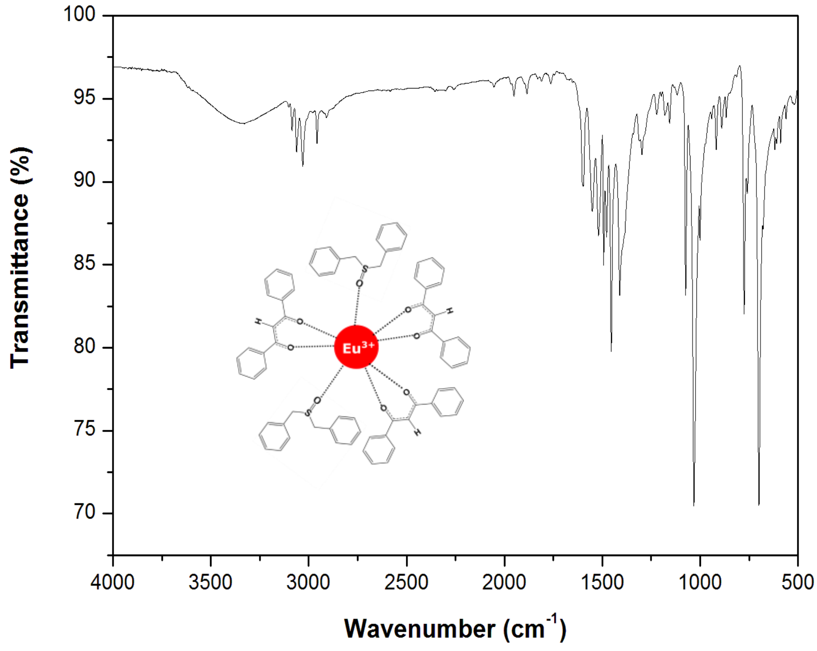

Eu(DBM)$_3$(DBSO)$_2$ R(KBr): ν=C-H 3083 cm^{-1} – 3032 cm^{-1} , ν CH_2 2960 cm^{-1} - 2913 cm^{-1}, ν C=O 1599 cm^{-1}, νS=O 1028 cm^{-1}.

S19 Fig. Infrared spectrum of Eu(DBM)$_3$(DBSO)$_2$ by faster synthesis route.

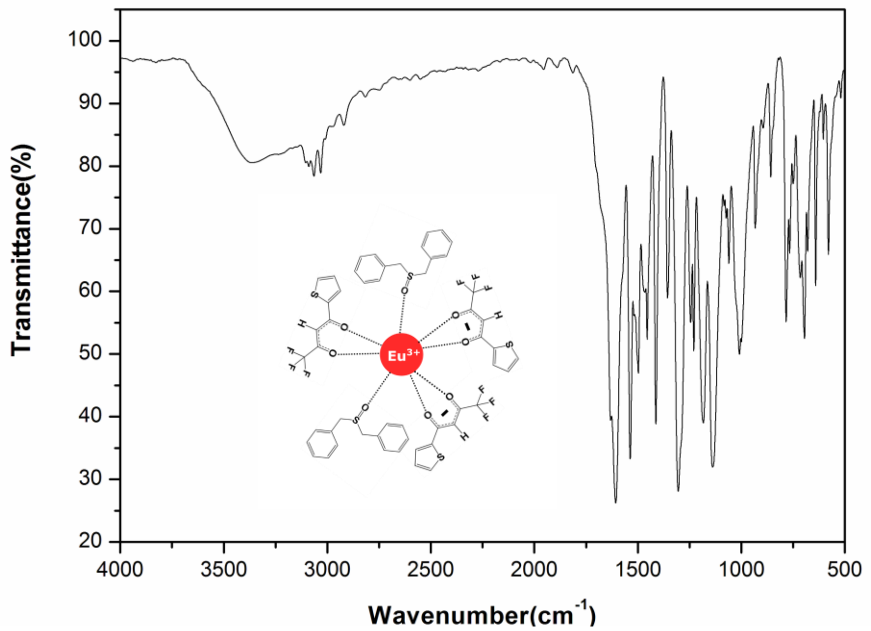

Eu(TTA)$_3$(DBSO)$_2$ (KBr): ν=C-H 3107 cm^{-1} – 3031 cm^{-1} , ν CH$_2$ 2975 cm^{-1} - 2919cm^{-1}, ν C=O 1606 cm^{-1}, νS=O 1012 cm^{-1}.

S20 Fig. Infrared spectrum of Eu(TTA)$_3$(DBSO)$_2$ by usual synthesis route.

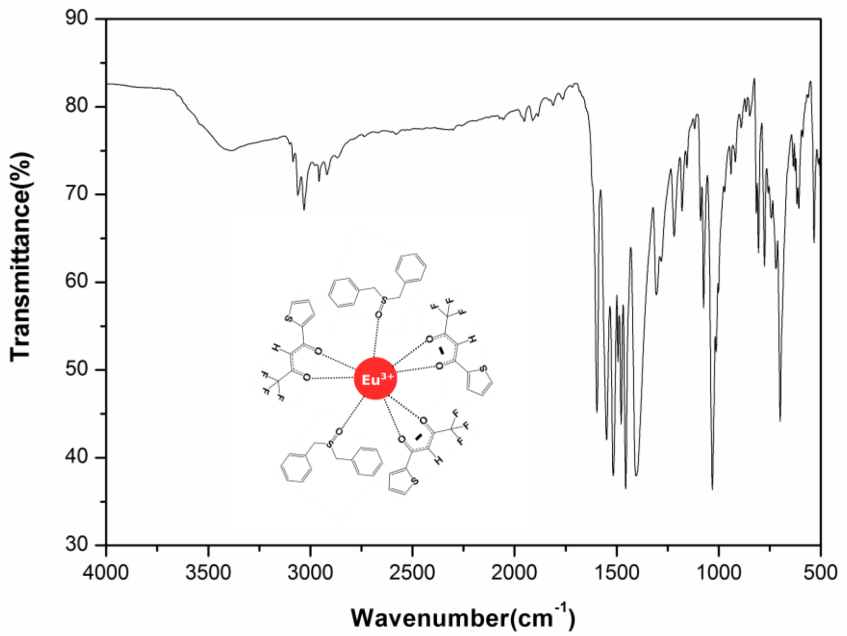

Eu(TTA)$_3$(DBSO)$_2$ (KBr): ν=C-H 3088 cm^{-1} – 3032 cm^{-1} , ν CH$_2$ 2975 cm^{-1} - 2918 cm^{-1}, ν C=O 1607 cm^{-1}, νS=O 1013 cm^{-1}.

S21 Fig. Infrared spectrum of Eu(TTA)$_3$(DBSO)$_2$ by faster synthesis route.

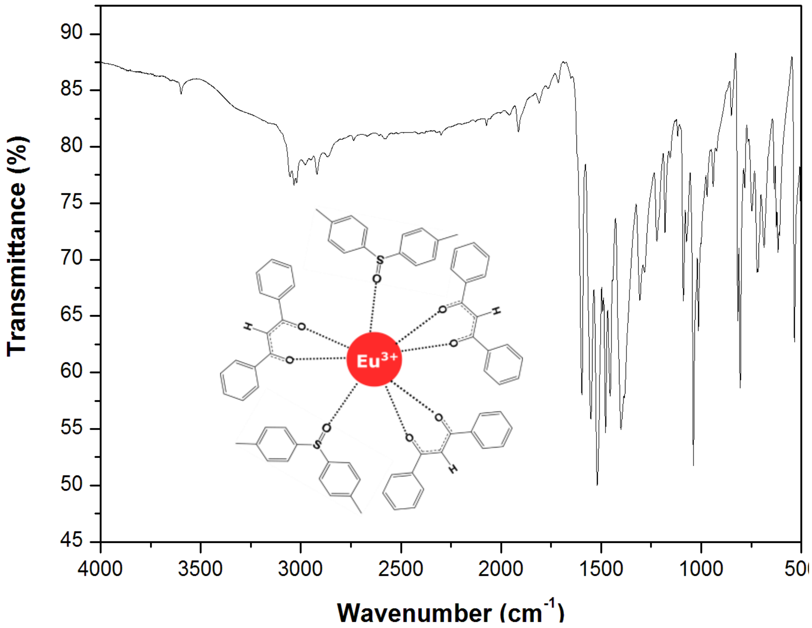

Eu(DBM)$_3$(PTSO)$_2$ (KBr): ν (=C-H) 3060 – 3013 cm^{-1} ,ν CH_3 2980 - 2861 cm^{-1},ν C=O 1595 cm^{-1}, νS=O 1012 cm^{-1}.

S22 Fig. Infrared spectrum of Eu(DBM)$_3$(PTSO)$_2$ by usual synthesis route.

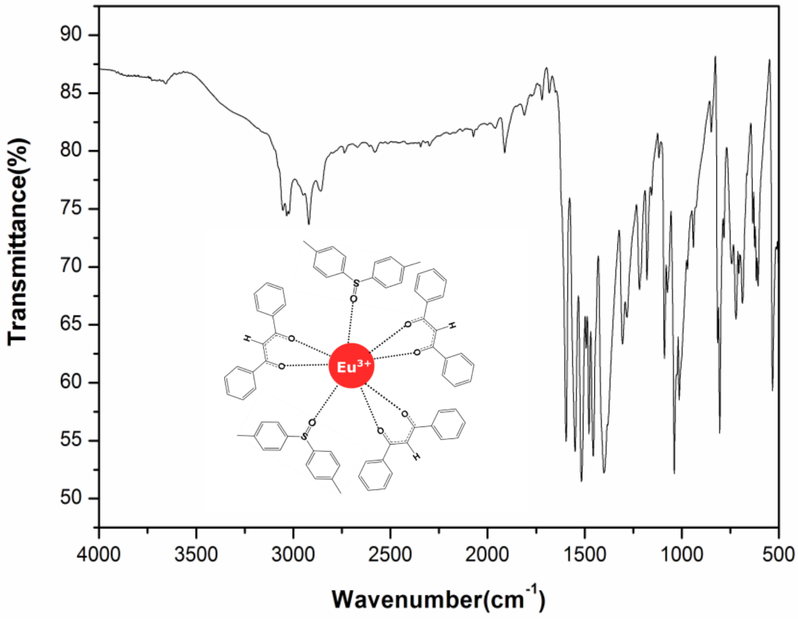

Eu(DBM)$_3$(PTSO)$_2$ (KBr): ν=C-H 3059 cm^{-1} – 3027 cm^{-1} , ν CH_3 2947cm^{-1} - 2857cm^{-1}, ν C=O 1599 cm^{-1}, νS=O 1018 cm^{-1}.

S23 Fig. Infrared spectrum of Eu(DBM)$_3$(PTSO)$_2$ by faster synthesis route.

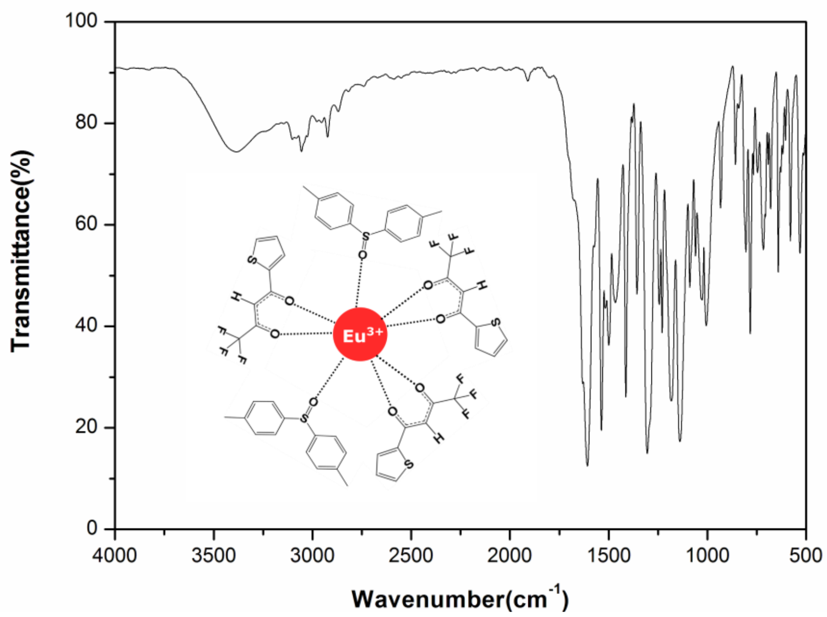

$Eu(TTA)_3(PTSO)_2$ (KBr): ν=C-H 3097 cm^{-1} – 3021 cm^{-1} , ν CH_3 2975 cm^{-1} - 2866 cm^{-1}, ν C=O 1603 cm^{-1}, νS=O 1022 cm^{-1}.

S24 Fig. Infrared spectrum of $Eu(TTA)_3(PTSO)_2$ by usual synthesis route.

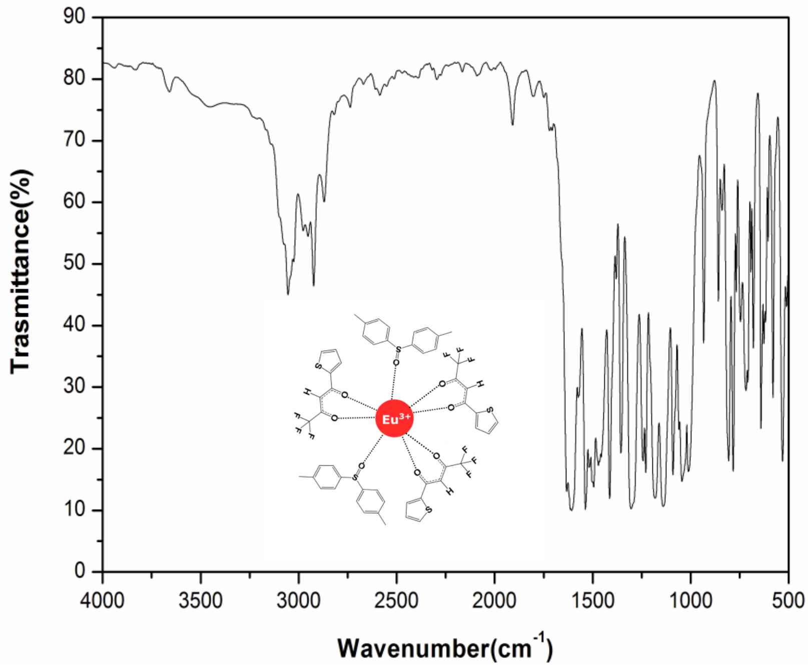

$Eu(TTA)_3(PTSO)_2$ (KBr): ν=C-H 3102 cm^{-1} – 3032 cm^{-1} , ν CH_3 2975 cm^{-1} - 2871 cm^{-1}, ν C=O 1604 cm^{-1}, νS=O 1012 cm^{-1}.

S25 Fig. Infrared spectrum of $Eu(TTA)_3(PTSO)_2$ by faster synthesis route.

NMR Spectra

All NMR spectra of all complexes were obtained in $CDCl_3$ solutions via a Varian Unity Plus 400 MHz equipament.

^{1}H NMR Spectra

S26-S53 Figs show ^{1}H NMR spectra for all free ligands and synthesized complexes.

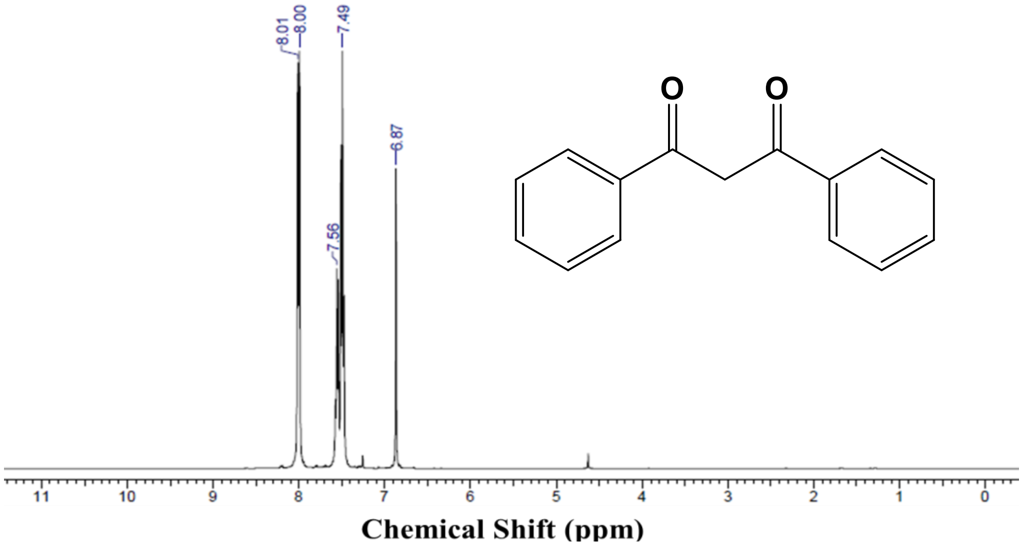

^{1}H NMR (400 MHz, $CDCl_3$): δ8.01 - 6.67 (m, Ar.), 4.69 (m, CH_2).

S26 Fig. ^{1}H NMR spectrum of DBMH free ligand.

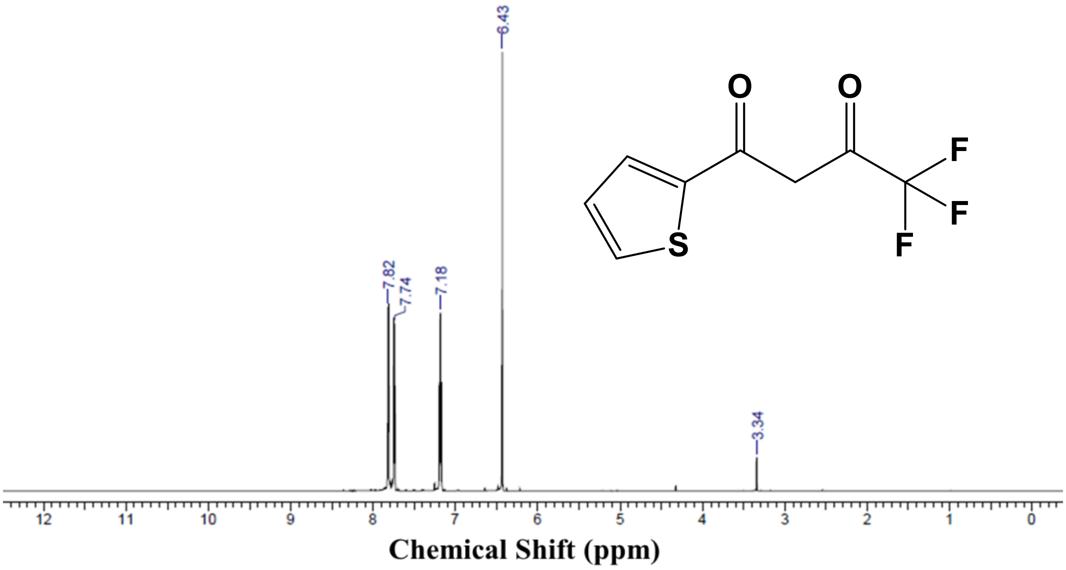

^{1}H NMR (400 MHz, $CDCl_3$): δ7.62 - 6.43 ppm (m, Ar.), 3.34 ppm (m, CH_2).

S27 Fig. ^{1}H NMR spectrum of TTAH free ligand.

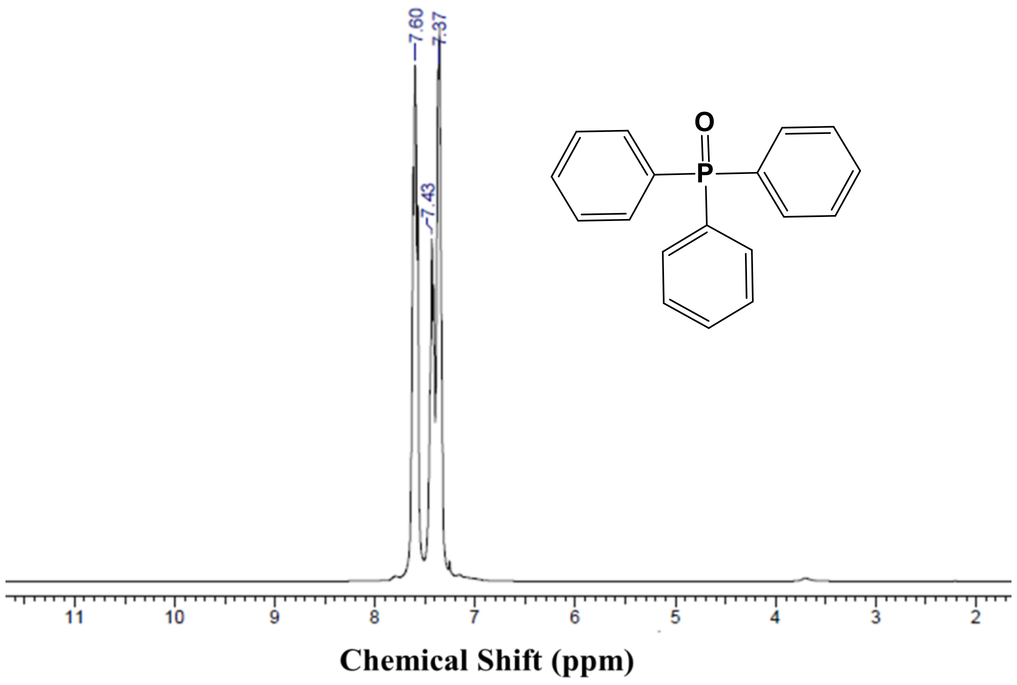

^{1}H NMR (400 MHz, $CDCl_3$): δ7.60 - 7.37 ppm (m, Ar.).

S28 Fig. ^{1}H NMR spectrum of TPPO free ligand.

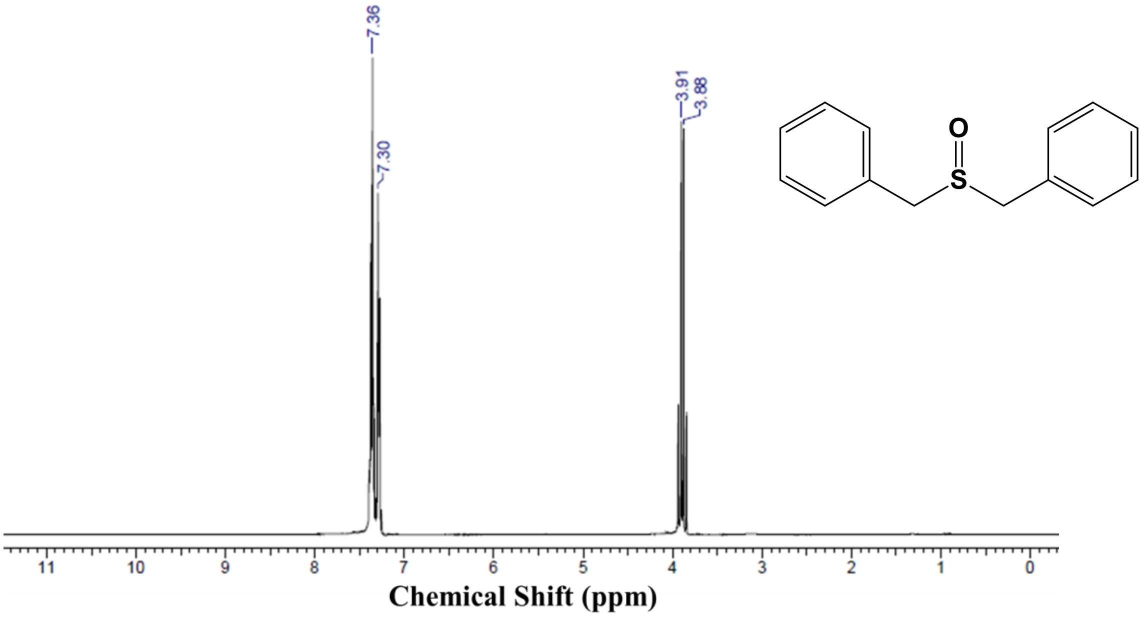

^{1}H NMR (400 MHz, $CDCl_3$): δ7.36 - 7.30 (m, Ar.), 3.91 -3.88 (m, CH_2).

S29 Fig. ^{1}H NMR spectrum of DBSO free ligand.

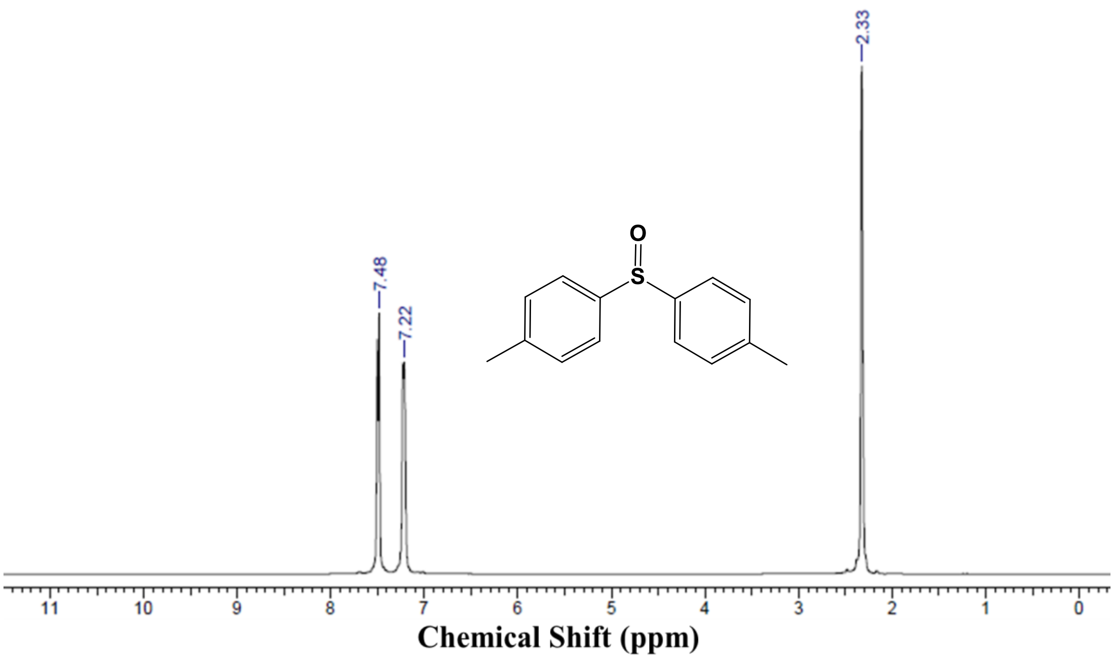

^{1}H NMR (400 MHz, $CDCl_3$): δ7.48 - 7.22 ppm (m, Ar.), 2.33 ppm (s,CH_3).

S30 Fig. ^{1}H NMR spectrum of PTSO free ligand.

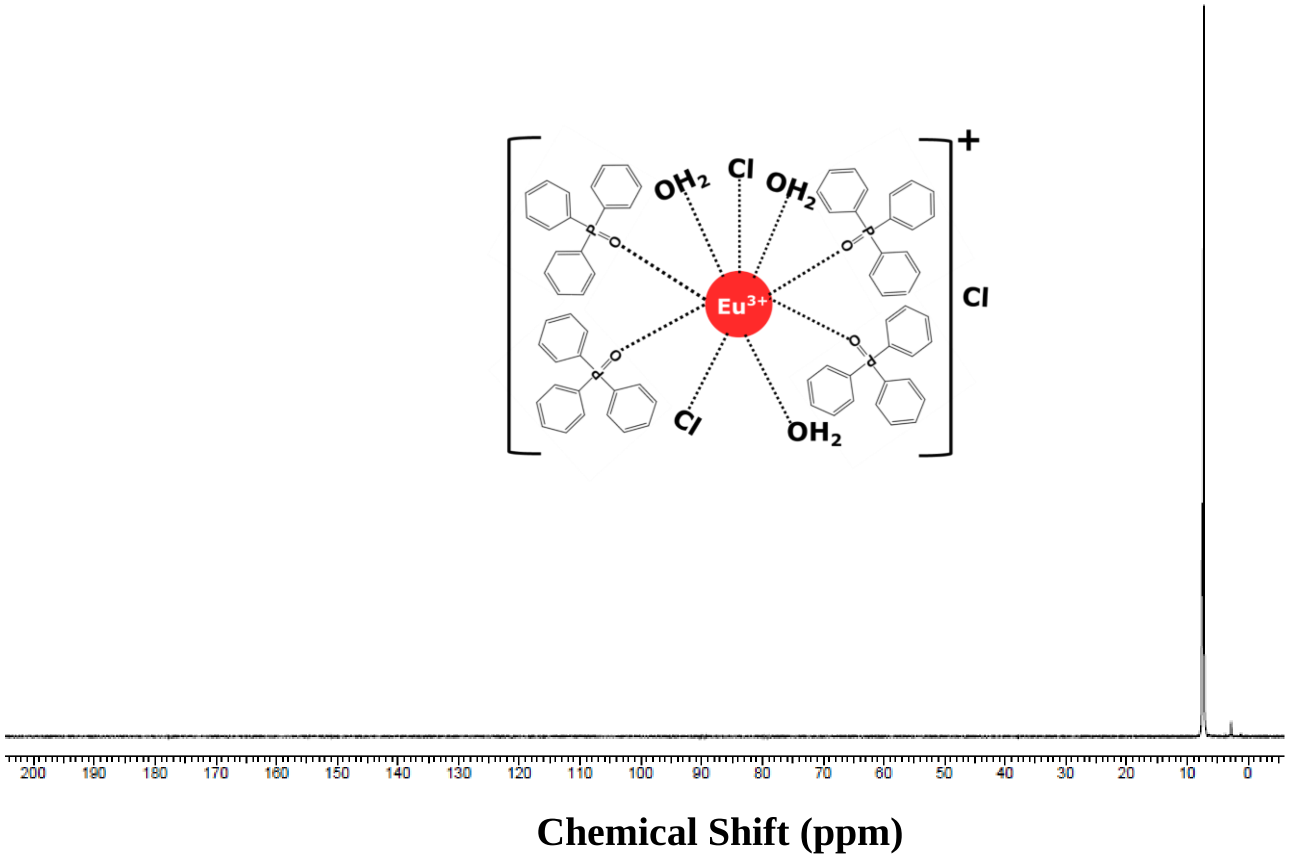

S31 Fig. ^{1}H NMR spectrum of $EuCl_3(TPPO)_4(H_2O)_3$, measured from 0 to 200 ppm to rule out the possibility that the charts could contain reflection signals of protons outside of the usual 0-20ppm range.

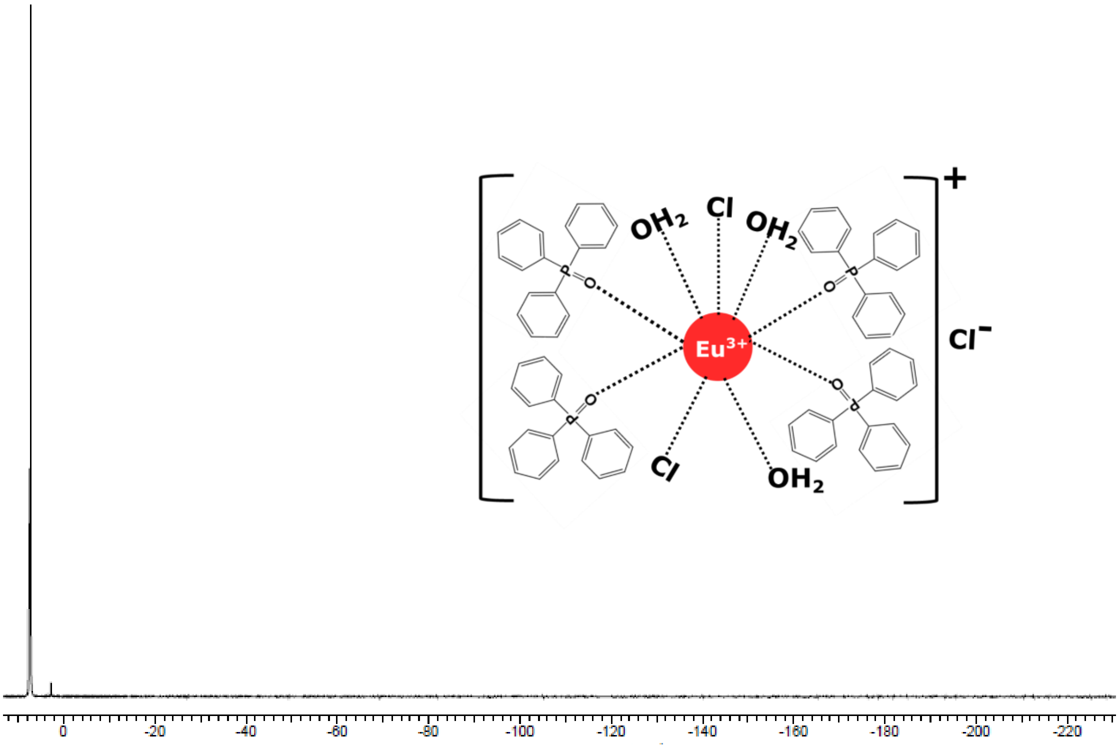

Chemical Shift (ppm)

S32 Fig. ^{1}H NMR spectrum of $EuCl_3(TPPO)_4(H_2O)_3$, , measured from 20 to -200 ppm to rule out the possibility that the charts could contain reflection signals of protons outside of the usual 0-20ppm range.

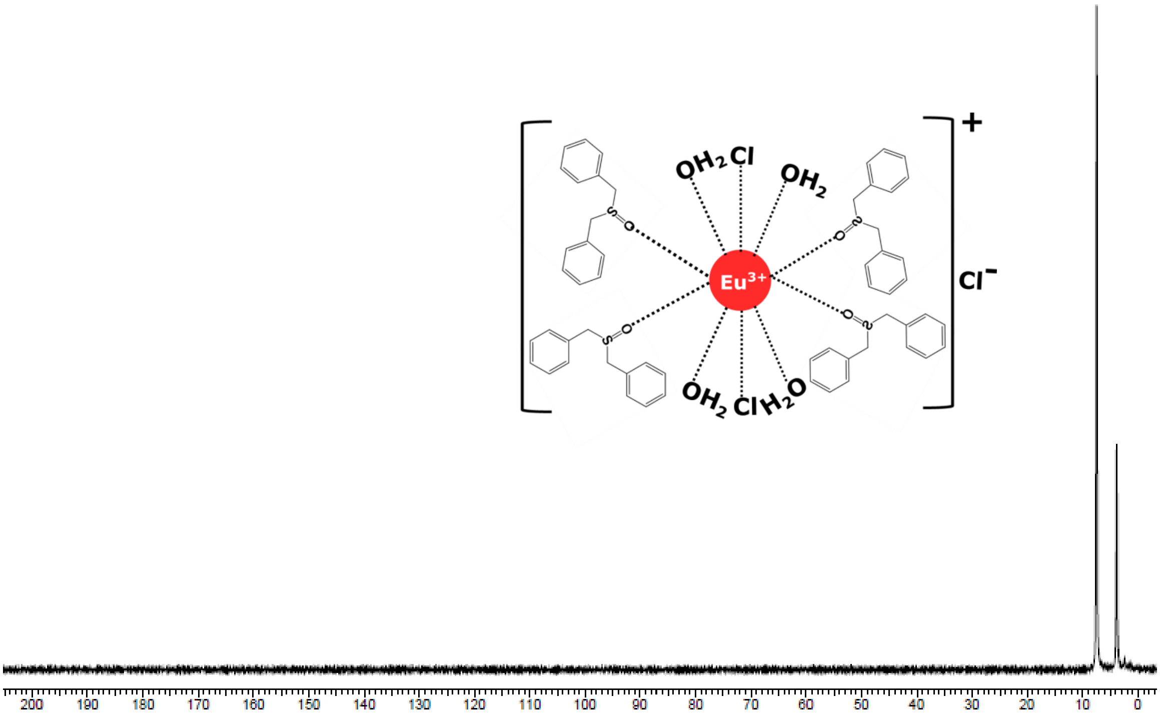

Chemical Shift (ppm)

S33 Fig. ^{1}H NMR spectrum of $EuCl_3(DBSO)_4(H_2O)_4$, measured from 0 to 200 ppm to rule out the possibility that the charts could contain reflection signals of protons outside of the usual 0-20ppm range.

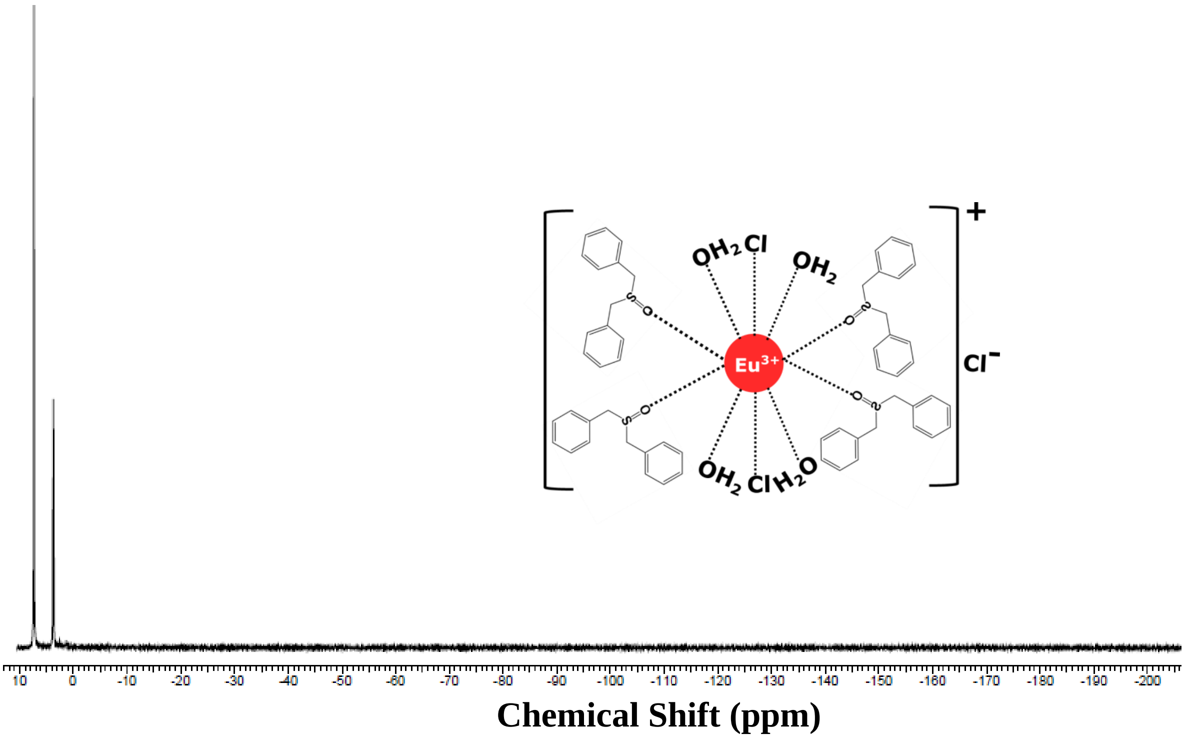

S34 Fig. ^{1}H NMR spectrum of $EuCl_3(DBSO)_4(H_2O)_4$, , measured from 20 to -200 ppm to rule out the possibility that the charts could contain reflection signals of protons outside of the usual 0-20ppm range.

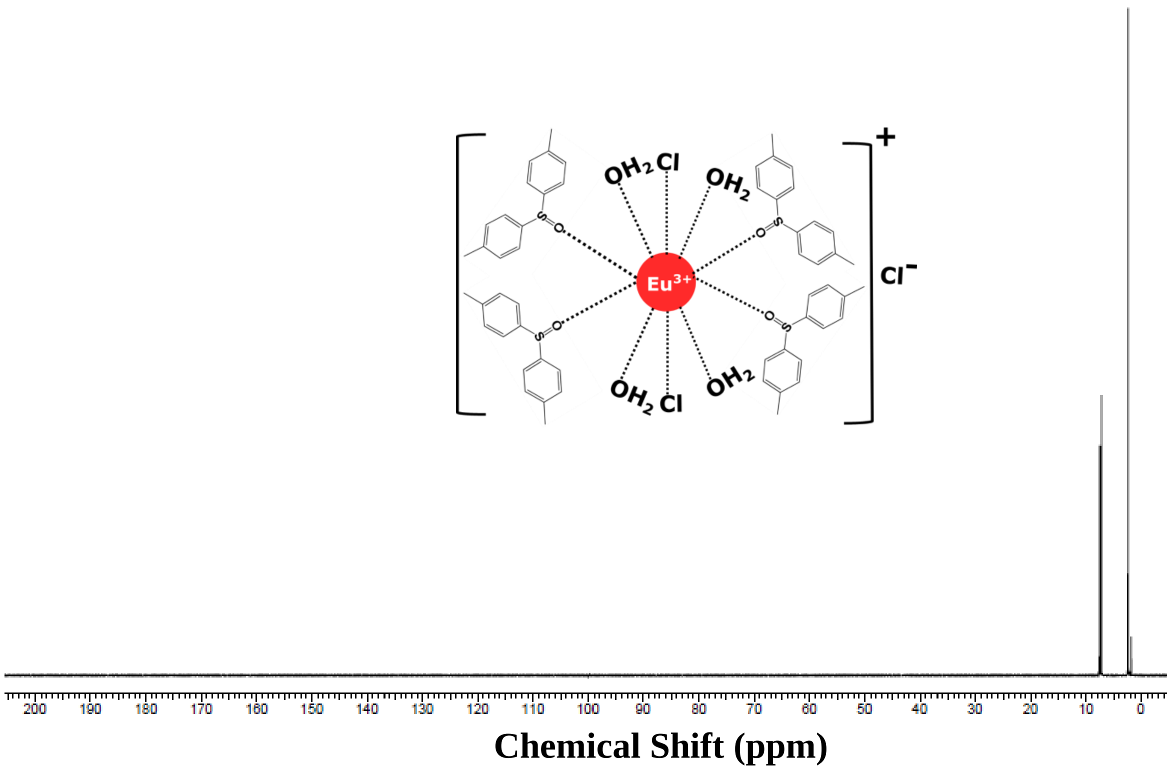

S35 Fig. ^{1}H NMR spectrum of $EuCl_3(PTSO)_4(H_2O)_4$, measured from 0 to 200 ppm to rule out the possibility that the charts could contain reflection signals of protons outside of the usual 0-20ppm range.

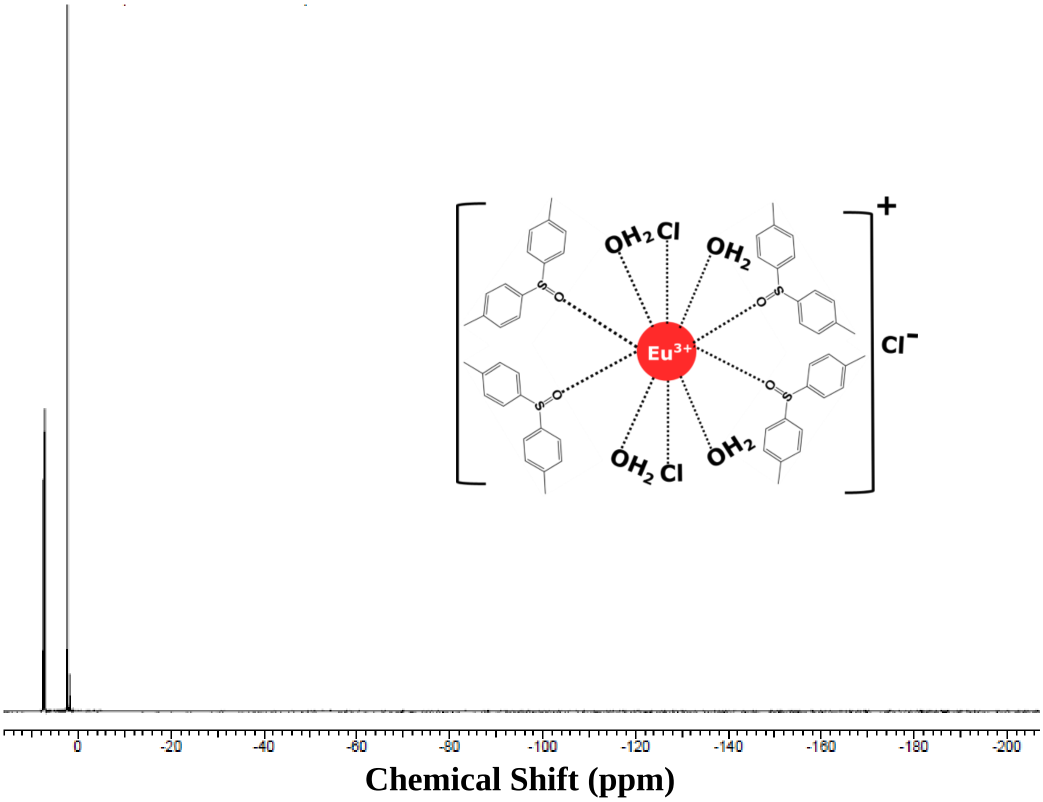

S36 Fig. ^{1}H NMR spectrum of $EuCl_3(PTSO)_4(H_2O)_4$, measured from 20 to -200 ppm to rule out the possibility that the charts could contain reflection signals of protons outside of the usual 0-20ppm range.

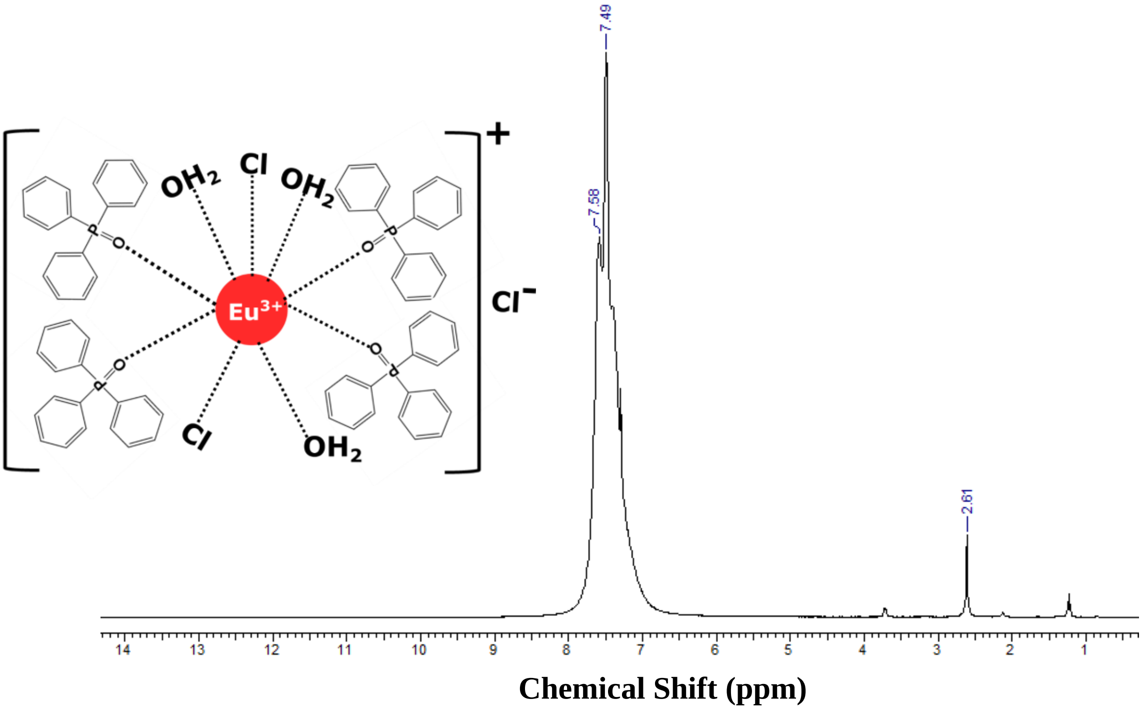

^{1}H NMR (400 MHz, $CDCl_3$): δ7.43 (m, Ar.), 2.62 (s,OH).

S37 Fig. ^{1}H NMR spectrum of $EuCl_3(TPPO)_4(H_2O)_3$.

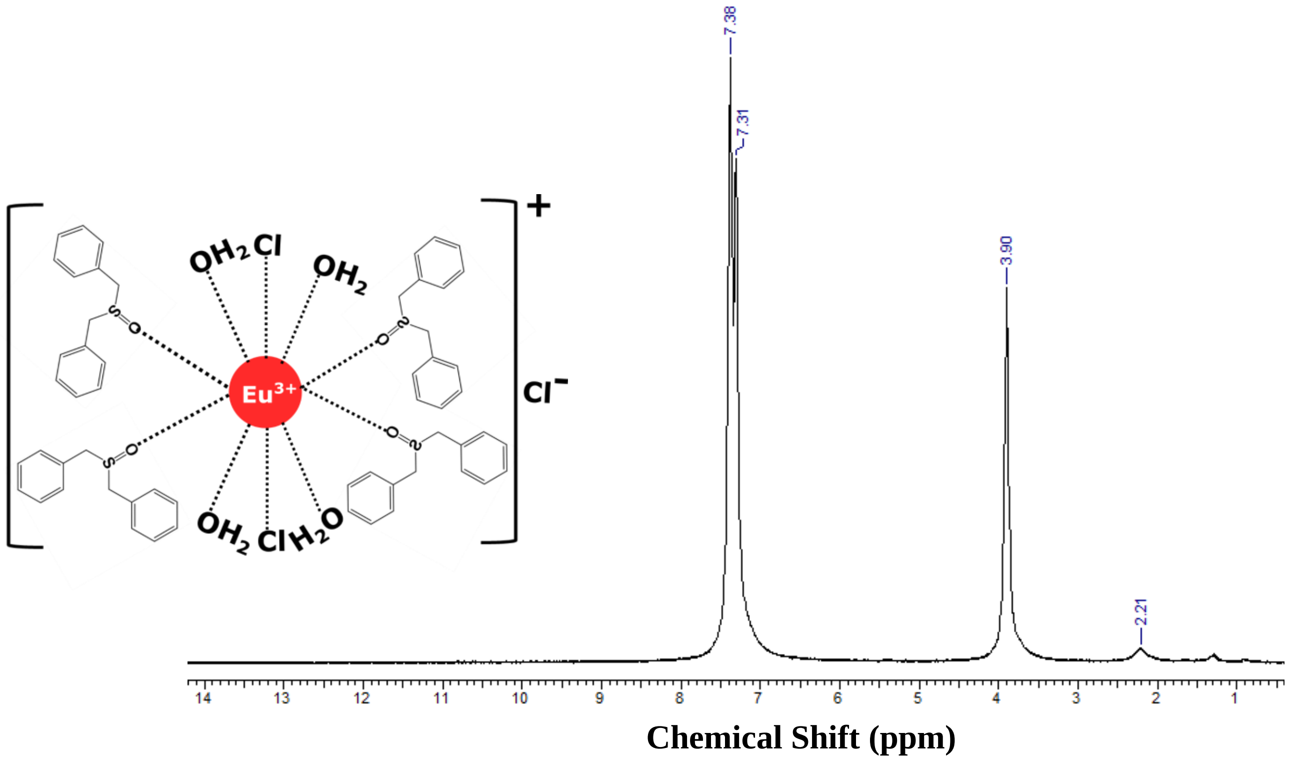

^{1}H NMR (400 MHz, CDCl$_3$): δ7.38-7.31 (m, Ar.) , 3.90 (m,CH_2) and 2.21 (s,OH).

S38 Fig. ^{1}H NMR spectrum of $EuCl_3(DBSO)_4(H_2O)_4$.

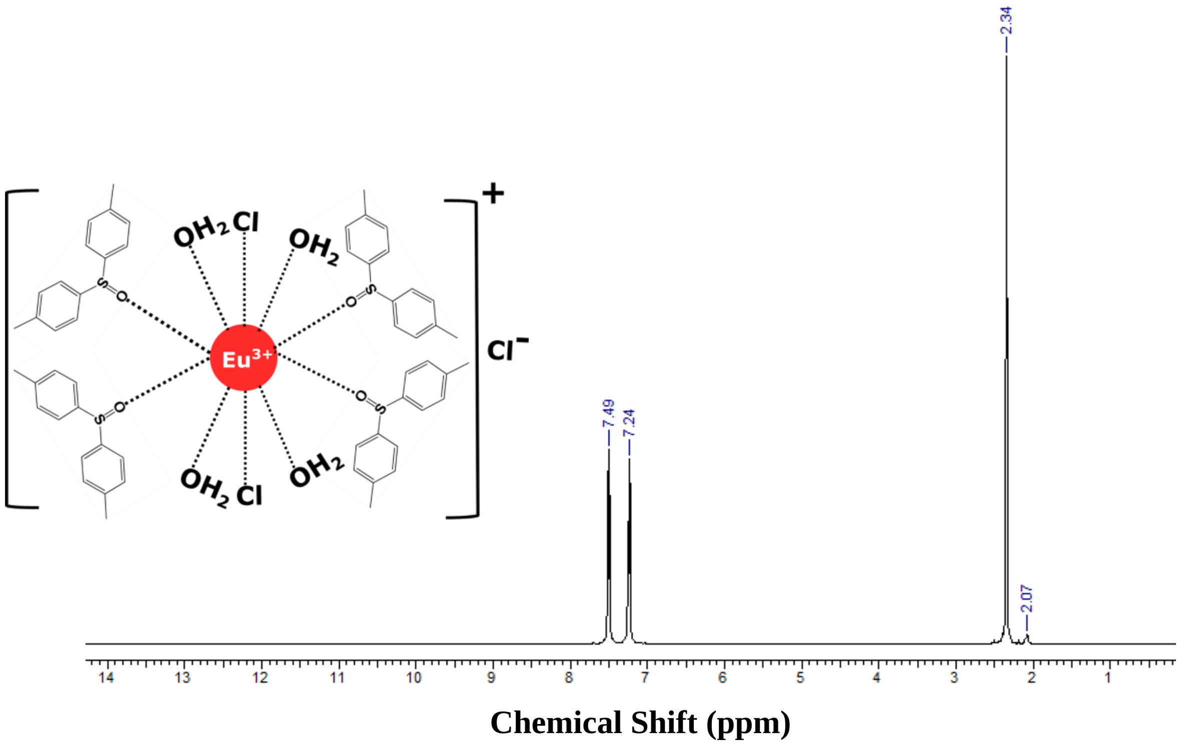

^{1}H NMR (400 MHz, CDCl$_3$): δ7.49-7.24 (m, Ar.), 2.34 (s,CH_3) and 2.07 (s,OH).

S39 Fig. ^{1}H NMR spectrum of $EuCl_3(PTSO)_4(H_2O)_4$.

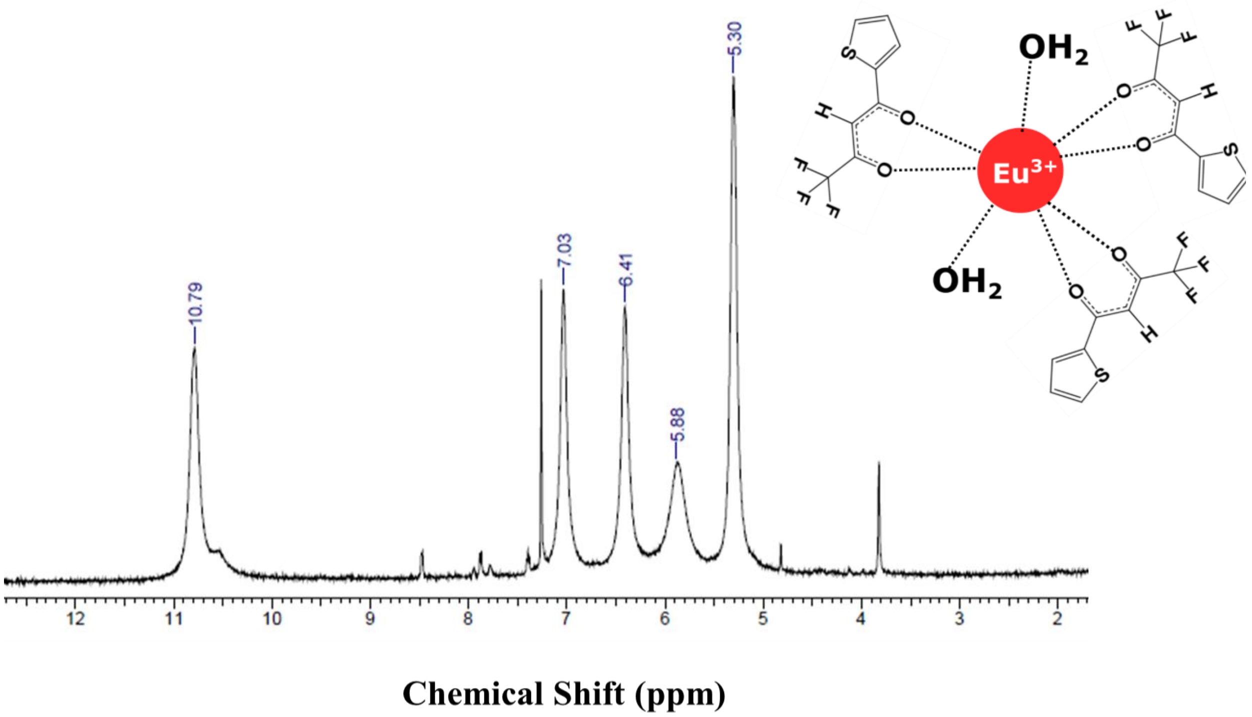

^{1}H NMR (400 MHz, $CDCl_3$): δ 10.79 (s,CH),7.03-5.30 (m, Ar.).

S40 Fig. ^{1}H NMR spectrum of $Eu(TTA)_3(H_2O)_2$.

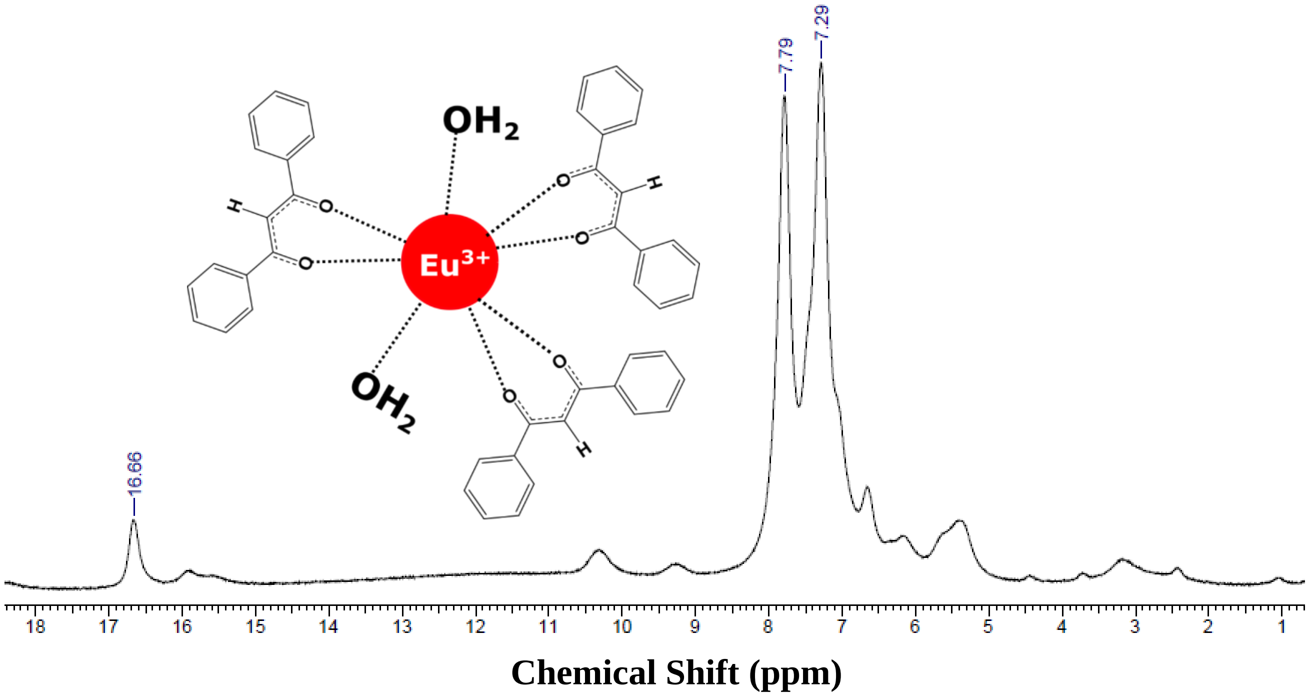

^{1}H NMR (400 MHz, $CDCl_3$): δ 16.66 (s,CH), 8.01-7.29 (m, Ar.).

S41 Fig. ^{1}H NMR spectrum of $Eu(DBM)_3(H_2O)_2$.

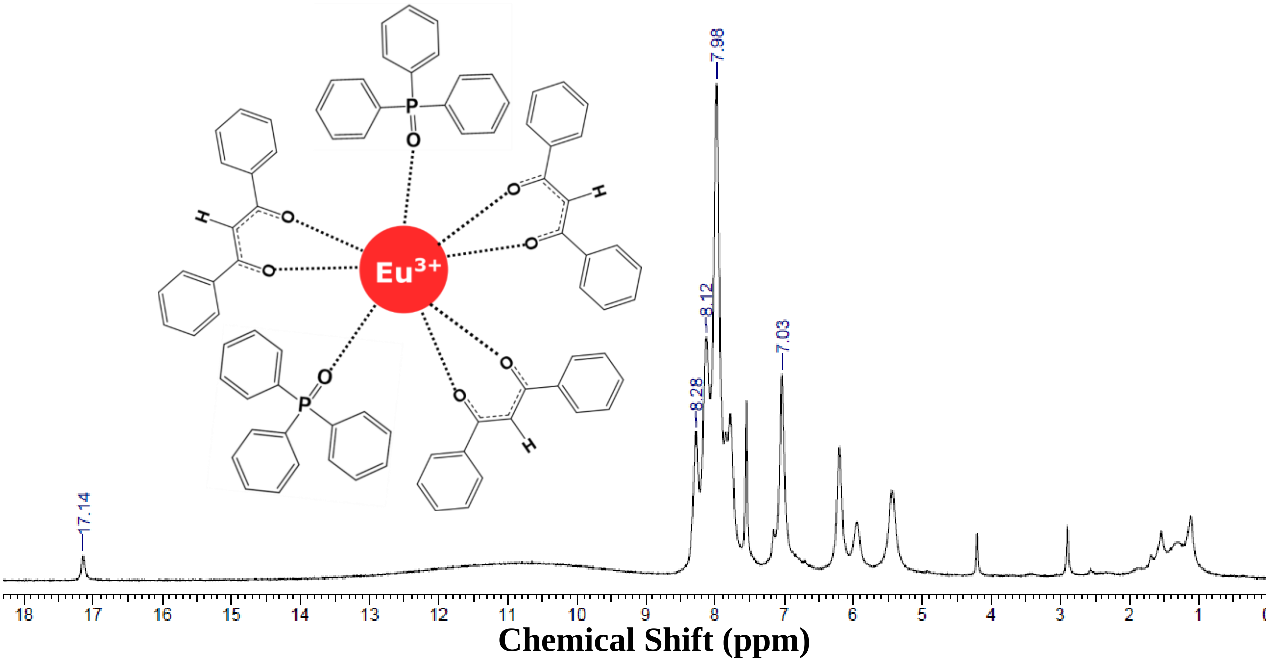

^{1}H NMR (400 MHz, CDCl$_3$): δ 17.14 (s,CH), 8.28-7.03 (m, Ar.).

S42 Fig. ^{1}H NMR spectrum of Eu(DBM)$_3$(TPPO)$_2$ of the usual synthesis.

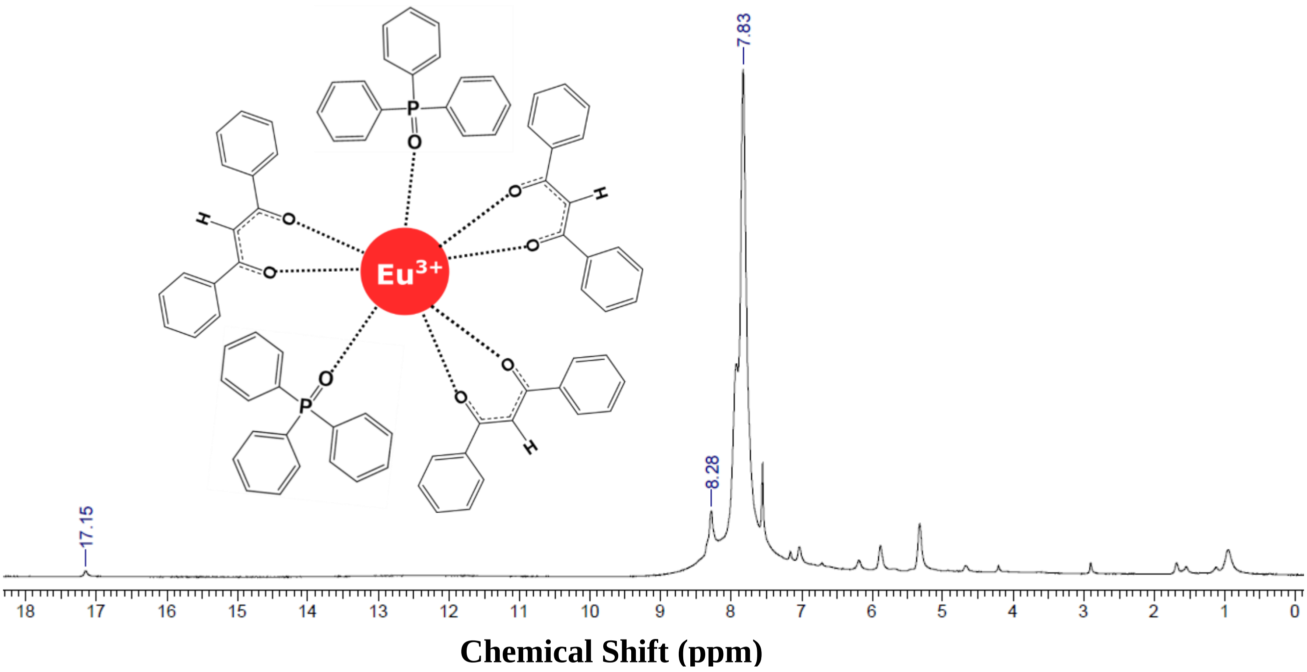

^{1}H NMR (400 MHz, CDCl$_3$): δ 17.15 (s,CH), 8.28-7.83 (m, Ar.).

S43 Fig. ^{1}H NMR spectrum of Eu(DBM)$_3$(TPPO)$_2$ of the faster synthesis.

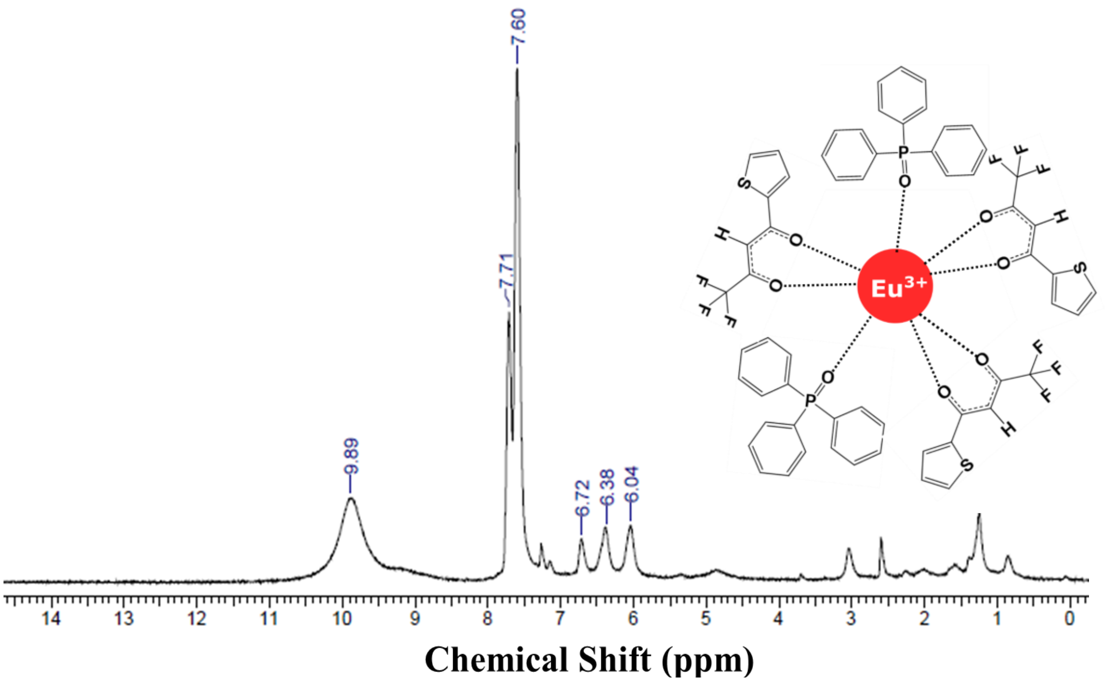

^{1}H NMR (400 MHz, $CDCl_3$): δ 9.89 (s,CH), 7.71-6.04 (m, Ar.).

S44 Fig. ^{1}H NMR spectrum of $Eu(TTA)_3(TPPO)_2$ of the usual synthesis.

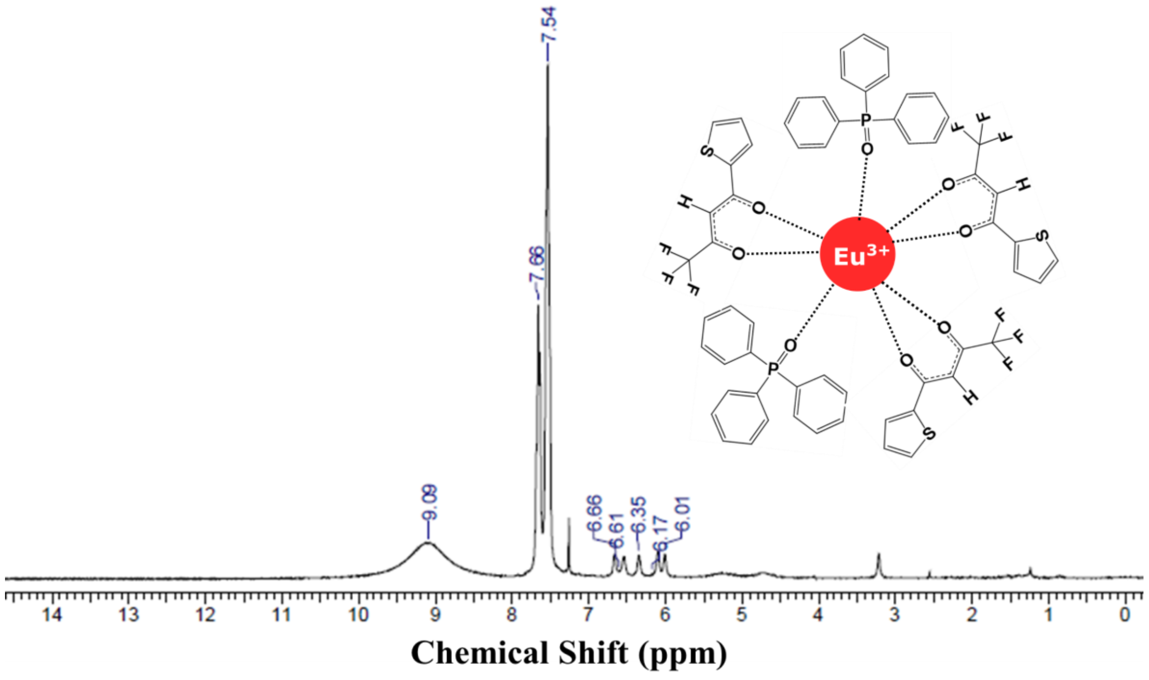

^{1}H NMR (400 MHz, $CDCl_3$): δ 9.09 (s,CH), 7.66-6.01 (m, Ar.).

S45 Fig. ^{1}H NMR spectrum of $Eu(TTA)_3(TPPO)_2$ of the faster synthesis.

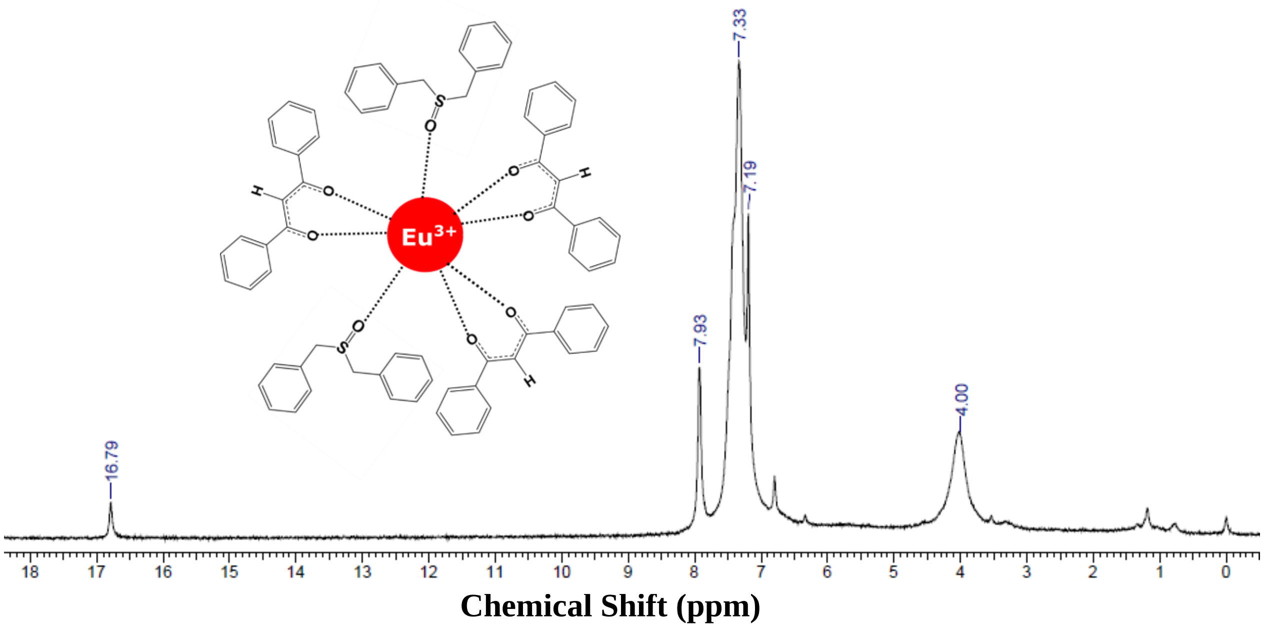

^{1}H NMR (400 MHz, $CDCl_3$): δ 16.79 (s,CH), 7.93-7.19 (m, Ar.) and 4.00 (m,CH_2).

S46 Fig. ^{1}H NMR spectrum of $Eu(DBM)_3(DBSO)_2$ of the usual synthesis.

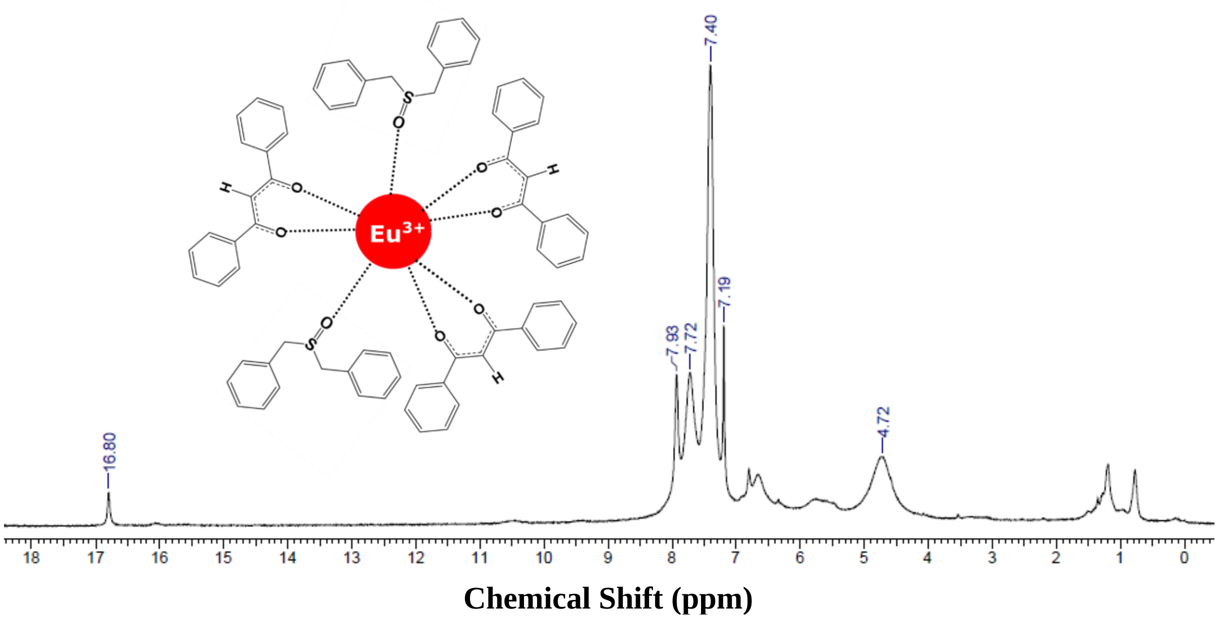

^{1}H NMR (400 MHz, $CDCl_3$): δ 16.80 (s, CH), 7.93-7.19 (m, Ar.) and 4.72 (m,CH_2).

S47 Fig. ^{1}H NMR spectrum of $Eu(DBM)_3(DBSO)_2$ of the faster synthesis.

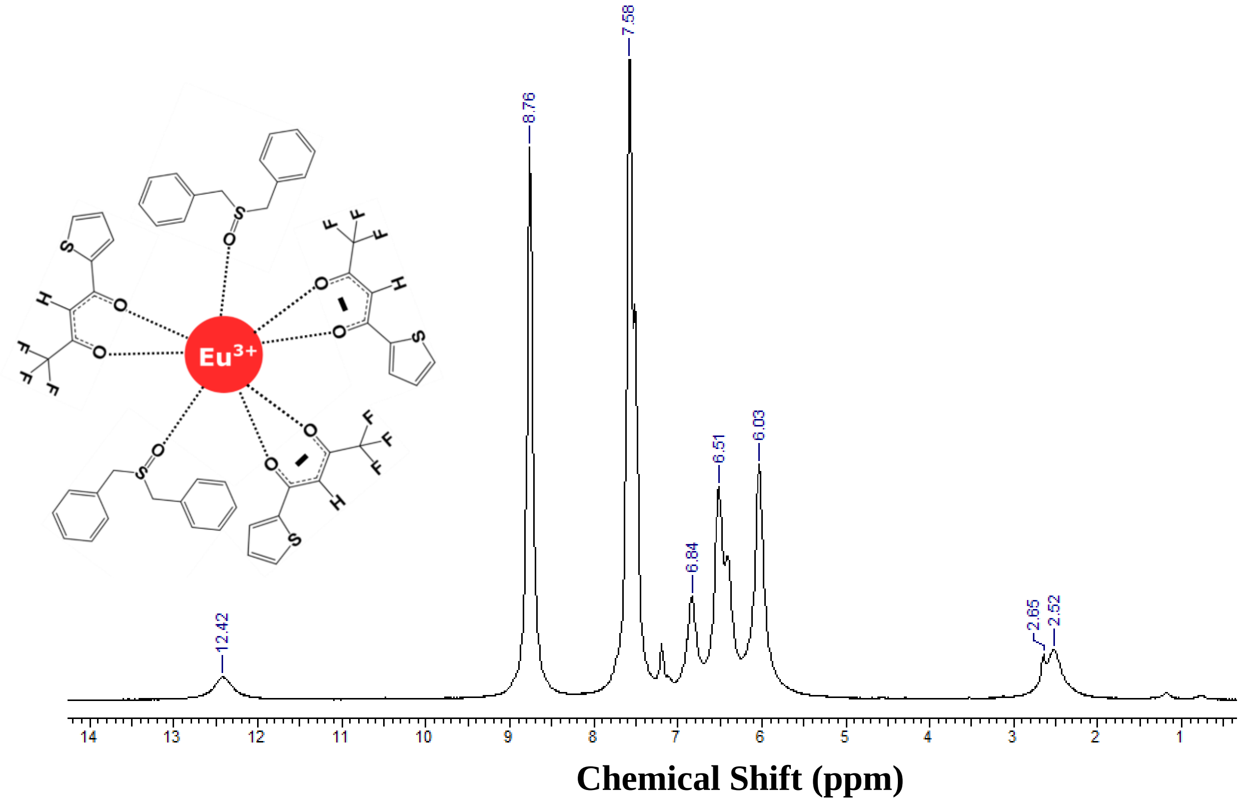

^{1}H NMR (400 MHz, $CDCl_3$): δ 12.42 (s, CH), 8.76 - 6.03 (m, Ar.) and 2.65-2.52 (m,CH_2).

S48 Fig. ^{1}H NMR spectrum of $Eu(TTA)_3(DBSO)_2$ of the usual synthesis.

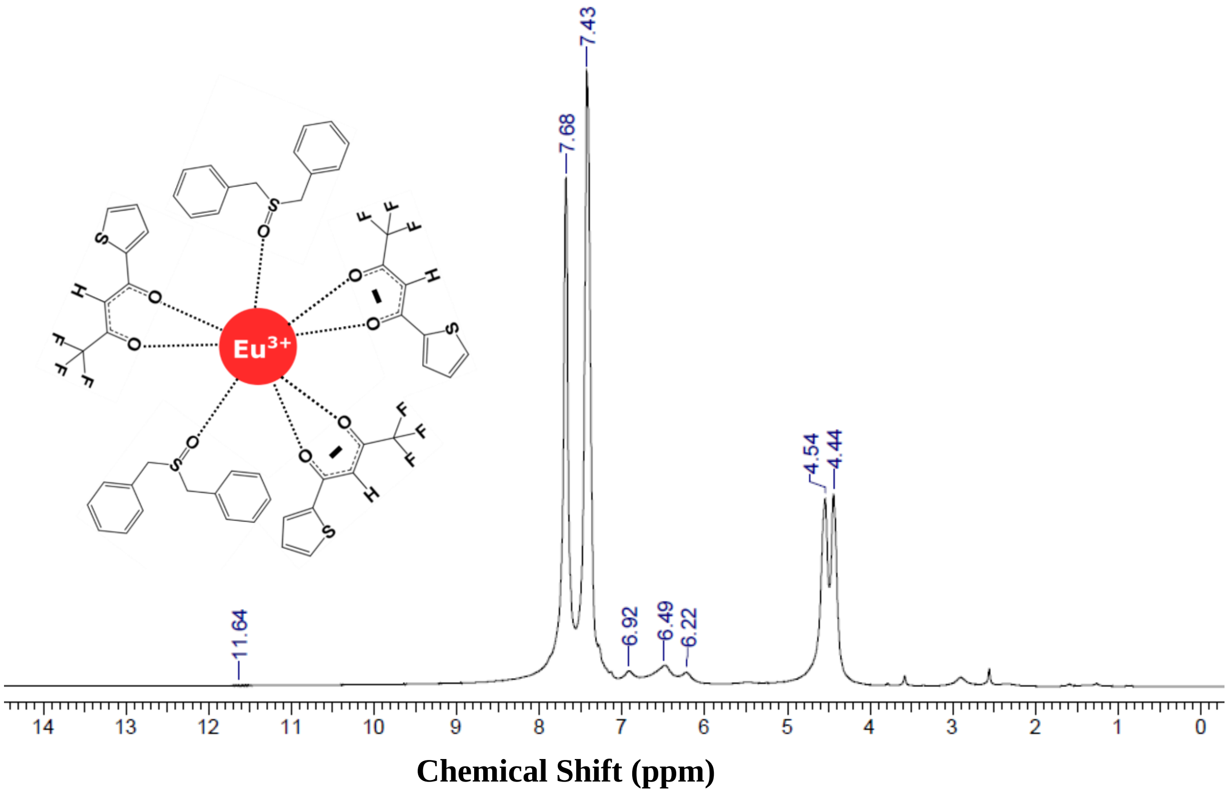

^{1}H NMR (400 MHz, $CDCl_3$): δ 11.64 (s,CH), 7.68 – 6.22 (m, Ar.) and 4.54-4.44 (m,CH_2).

S49 Fig. ^{1}H NMR spectrum of $Eu(TTA)_3(DBSO)_2$ of the faster synthesis.

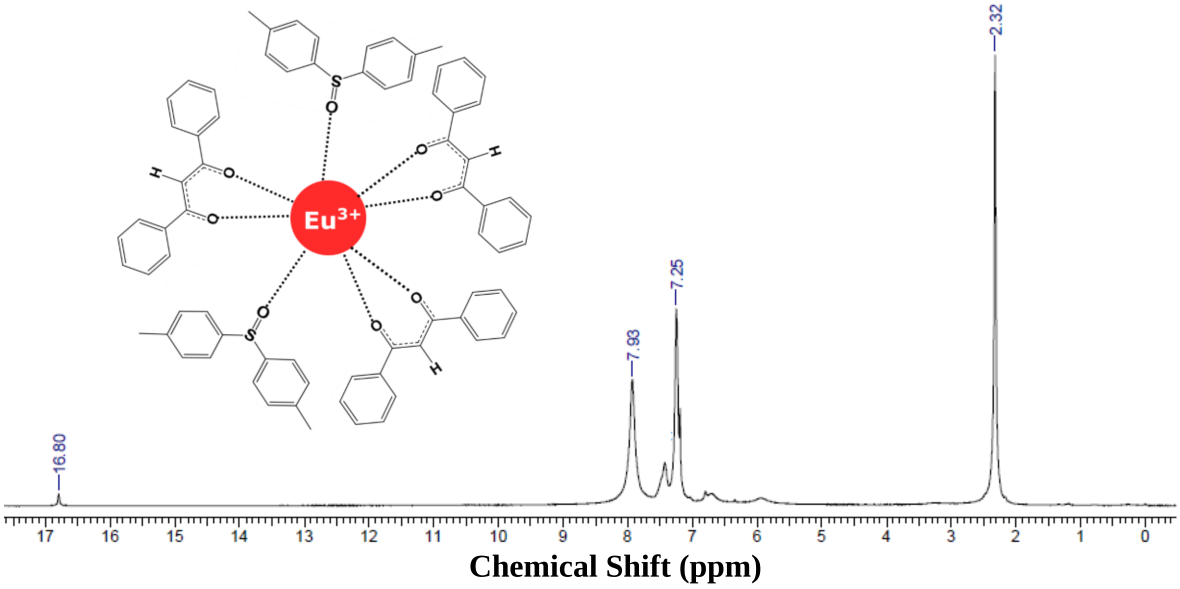

^{1}H NMR (400 MHz, $CDCl_3$): δ 16.80 (s,CH) 7.93- 7.25 (m, Ar.) and 2.32 (m,CH_3).

S50 Fig. ^{1}H NMR spectrum of $Eu(DBM)_3(PTSO)_2$ of the usual synthesis.

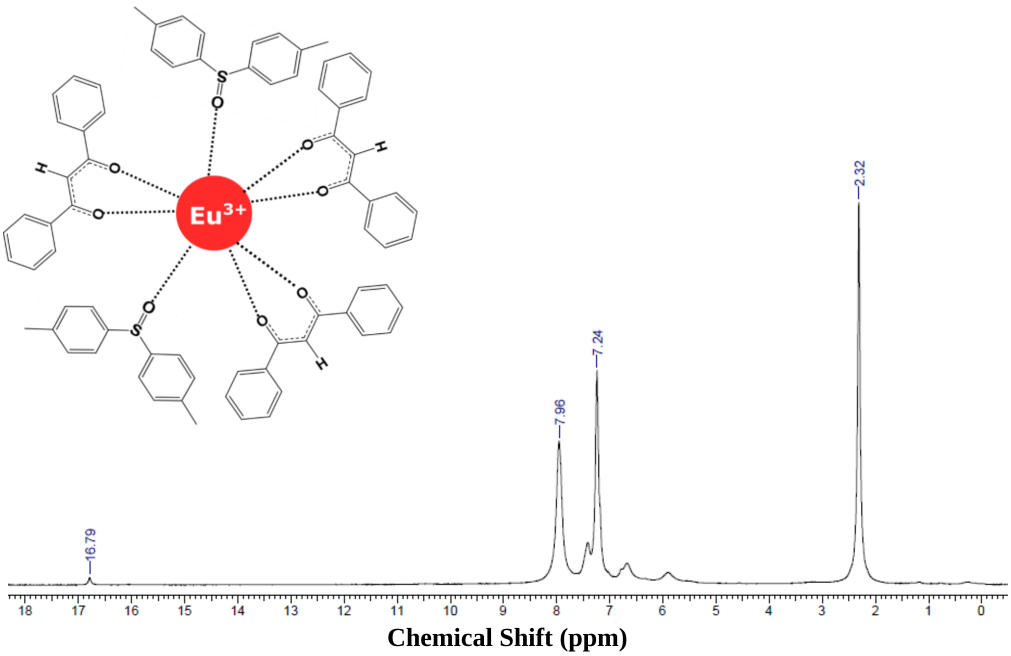

^{1}H NMR (400 MHz, $CDCl_3$): δ 16.79 (s,CH) 7.96- 7.24 (m, Ar.) and 2.32 (m,CH_3).

S51 Fig. ^{1}H NMR spectrum of $Eu(DBM)_3(PTSO)_2$ of the faster synthesis.

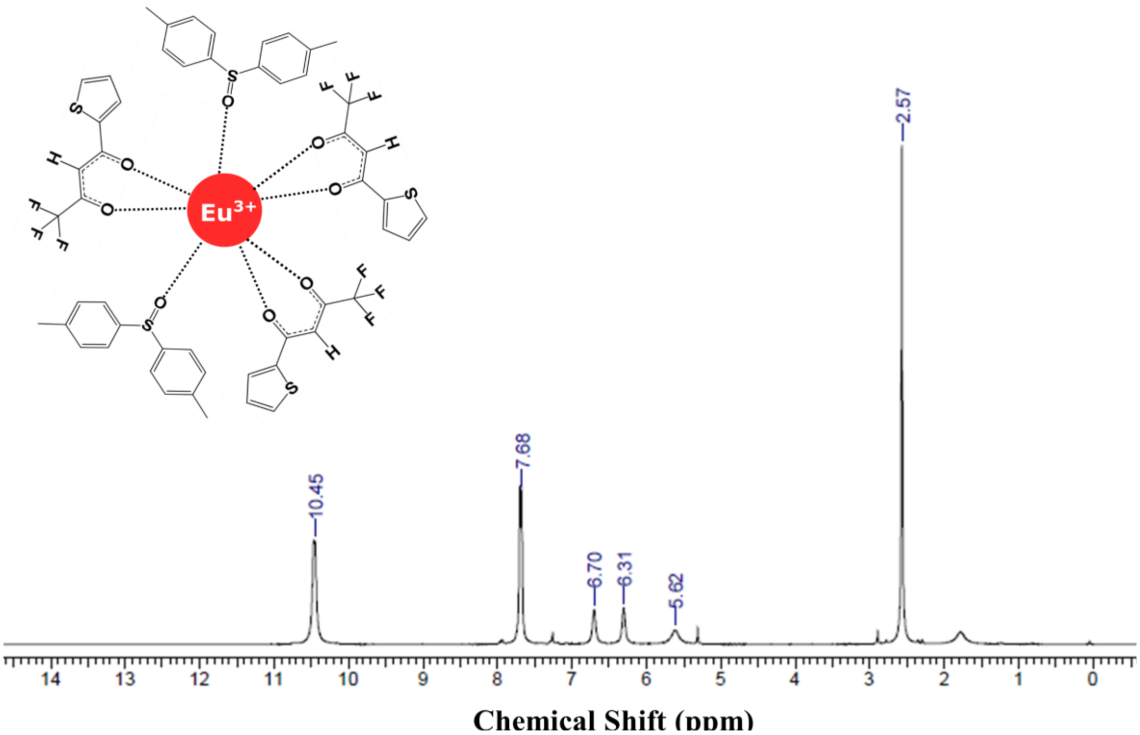

^{1}H NMR (400 MHz, $CDCl_3$): δ 10.45 (s,CH), 7.68- 5.62 (m, Ar.) and 2.57 (m,CH_3).

S52 Fig. ^{1}H NMR spectrum of $Eu(TTA)_3(PTSO)_2$ of the usual synthesis.

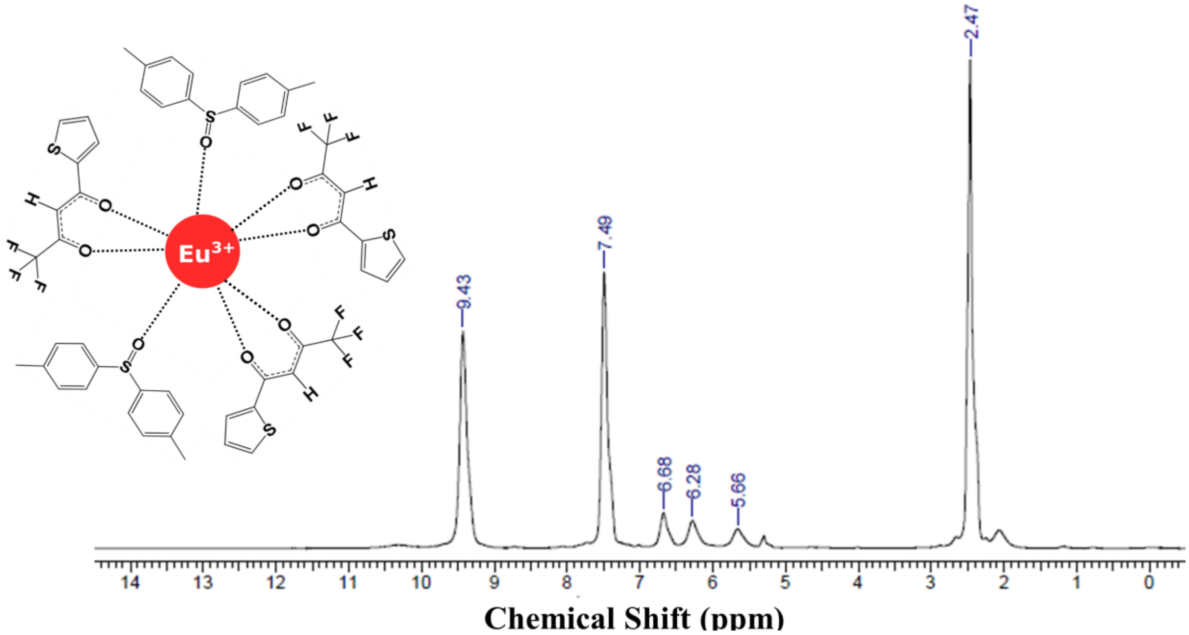

^{1}H NMR (400 MHz, $CDCl_3$): δ 9.43 (s,CH_3), 7.49- 5.66 (m, Ar.) and 2.47 (m,CH_3).

S53 Fig. ^{1}H NMR spectrum of $Eu(TTA)_3(PTSO)_2$ of the faster synthesis.

^{19}F NMR Spectra

S54-S61 Figs show ^{19}F NMR spectra for the TTAH free ligand and for the all synthesized complexes with TTA ligand.

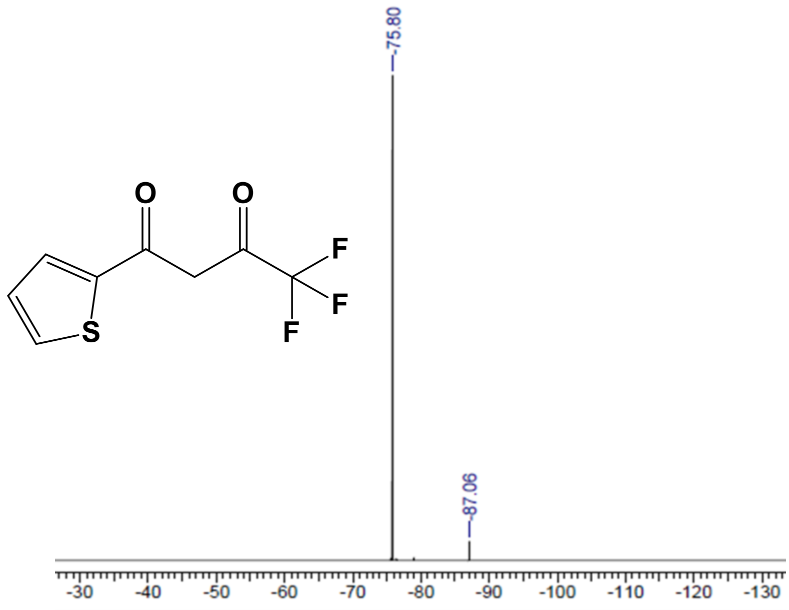

^{19}F NMR (400 MHz, $CDCl_3$): δ -75.80 and -87.06 ppm (CF_3).

S54 Fig. ^{19}F NMR spectrum of TTA free ligand.

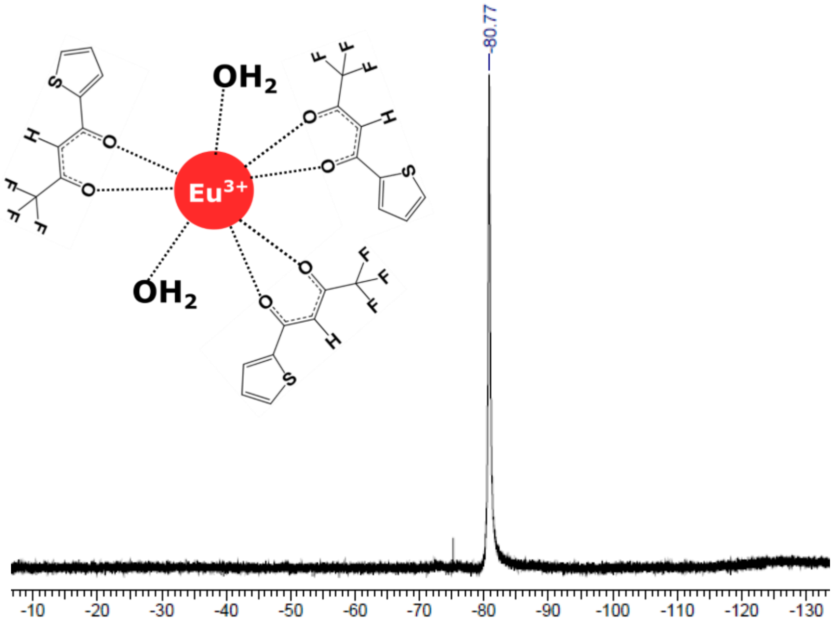

^{19}F NMR (400 MHz, $CDCl_3$): δ -80.77 ppm (CF_3).

S55 Fig. ^{19}F NMR spectrum of $Eu(TTA)_3(H_2O)_2$.

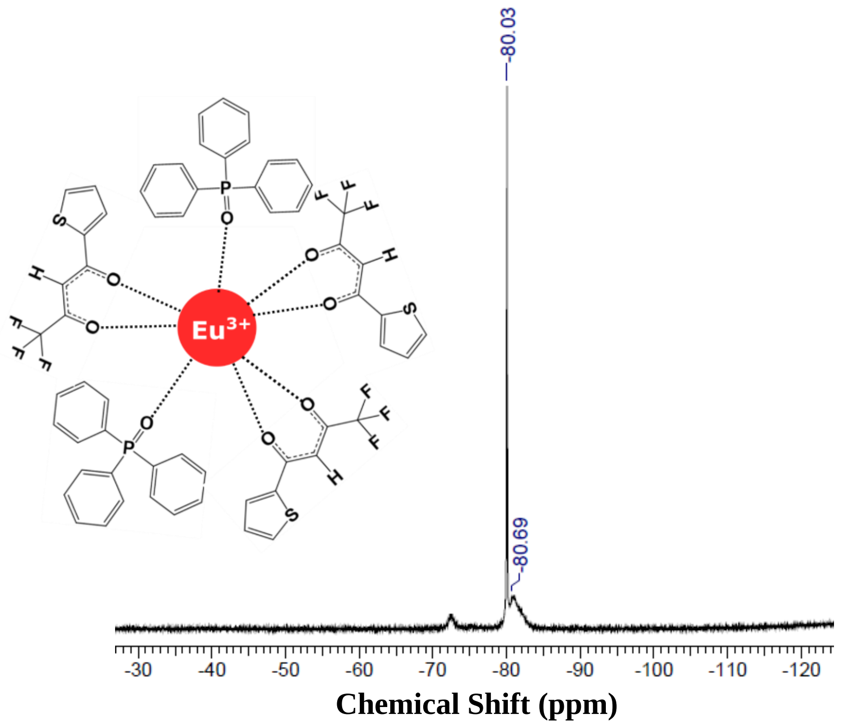

^{19}F NMR (400 MHz, $CDCl_3$): δ-80.03 to -80.69 ppm (CF_3).

S56 Fig. ^{19}F NMR spectrum of $Eu(TTA)_3(TPPO)_2$ of the usual synthesis.

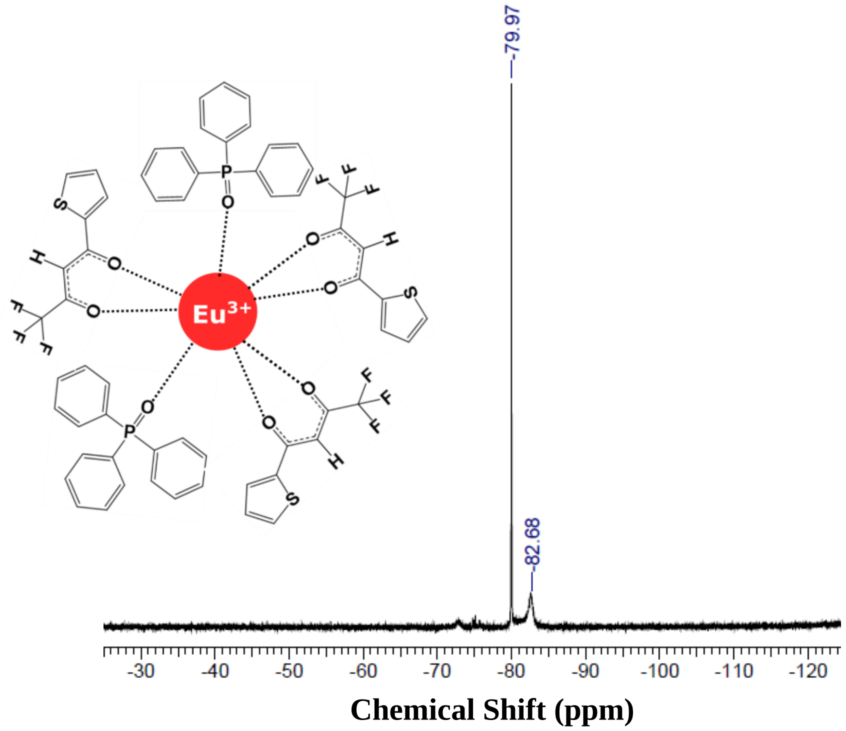

^{19}F NMR (400 MHz, $CDCl_3$): δ -79.97 to -82.68 ppm (CF_3).

S57 Fig. ^{19}F NMR spectrum of $Eu(TTA)_3(TPPO)_2$ of the faster synthesis.

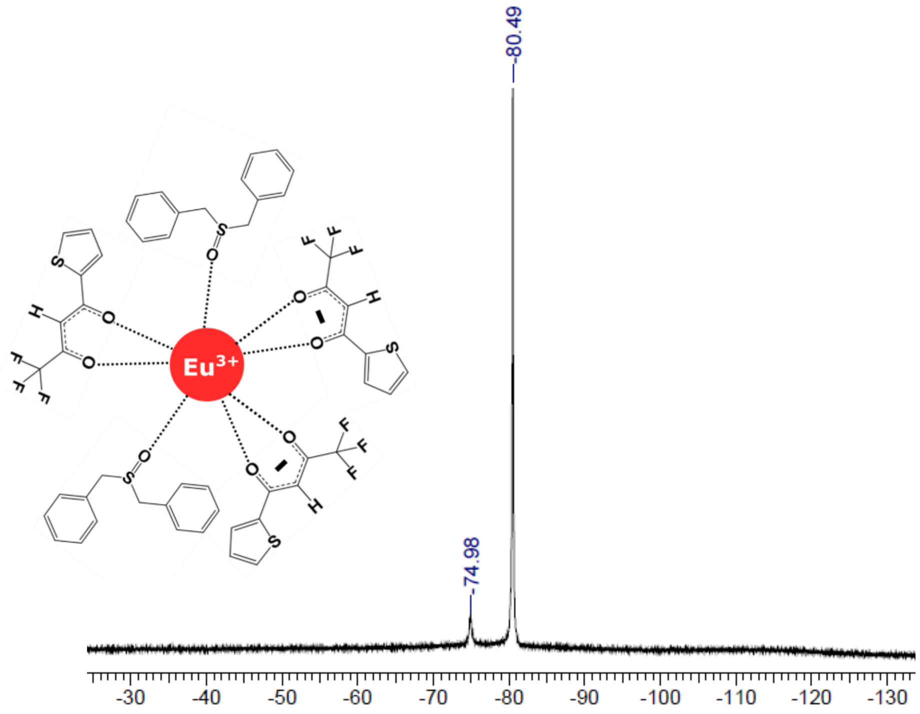

^{19}F NMR (400 MHz, $CDCl_3$): δ -74.98 to 80.49 ppm (CF_3).

S58 Fig. ^{19}F NMR spectrum of $Eu(TTA)_3(DBSO)_2$ of the usual synthesis.

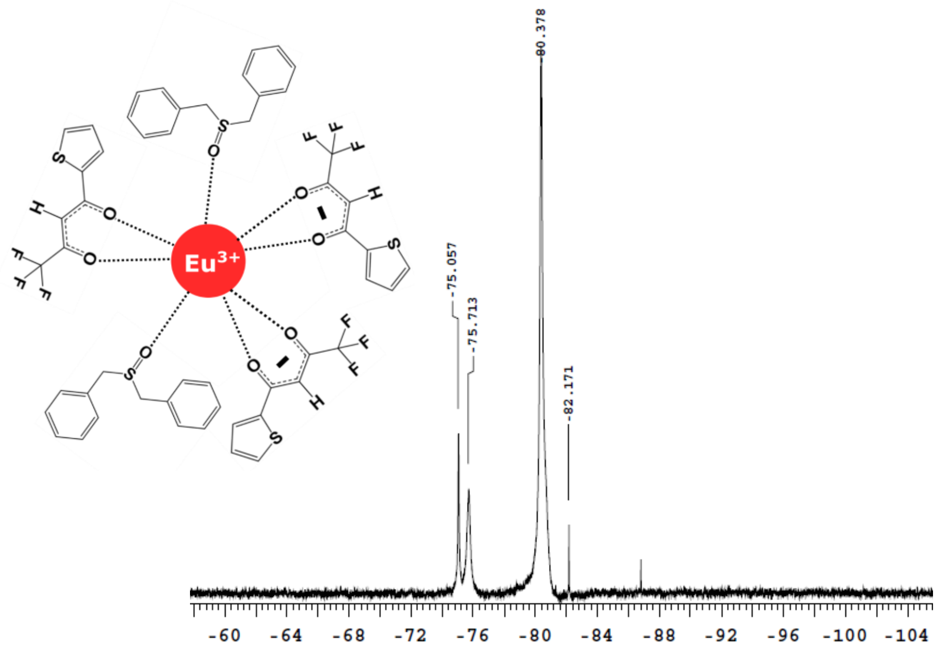

^{19}F NMR (400 MHz, $CDCl_3$): δ –75.06 to -82.18 ppm (CF_3).

S59 Fig. ^{19}F NMR spectrum of $Eu(TTA)_3(DBSO)_2$ of the faster synthesis.

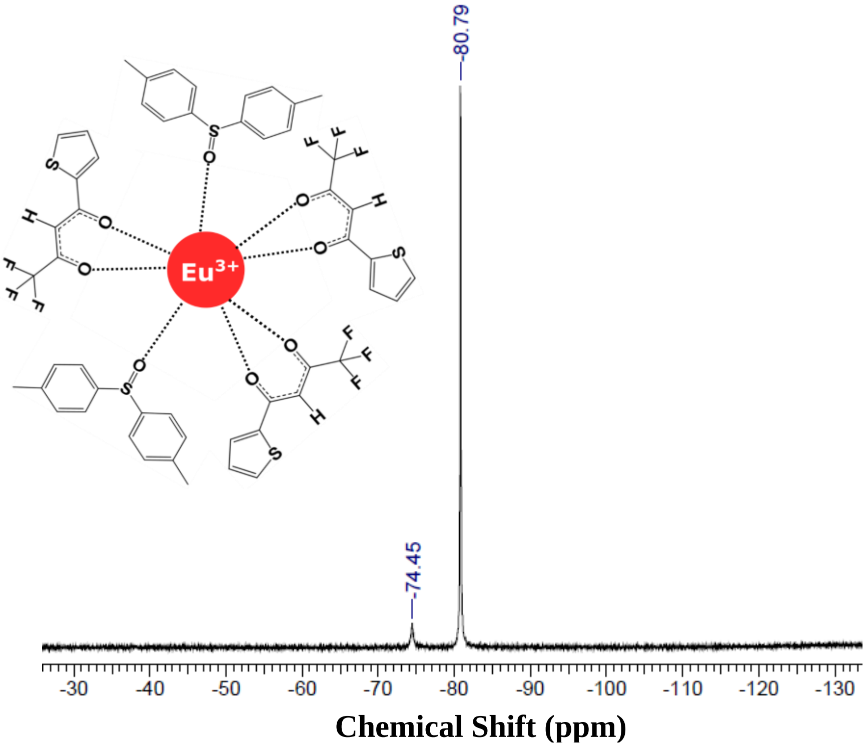

^{19}F NMR (400 MHz, $CDCl_3$): δ -74,45 to -80.79 ppm (CF_3).

S60 Fig. ^{19}F NMR spectrum of $Eu(TTA)_3(PTSO)_2$ of the usual synthesis.

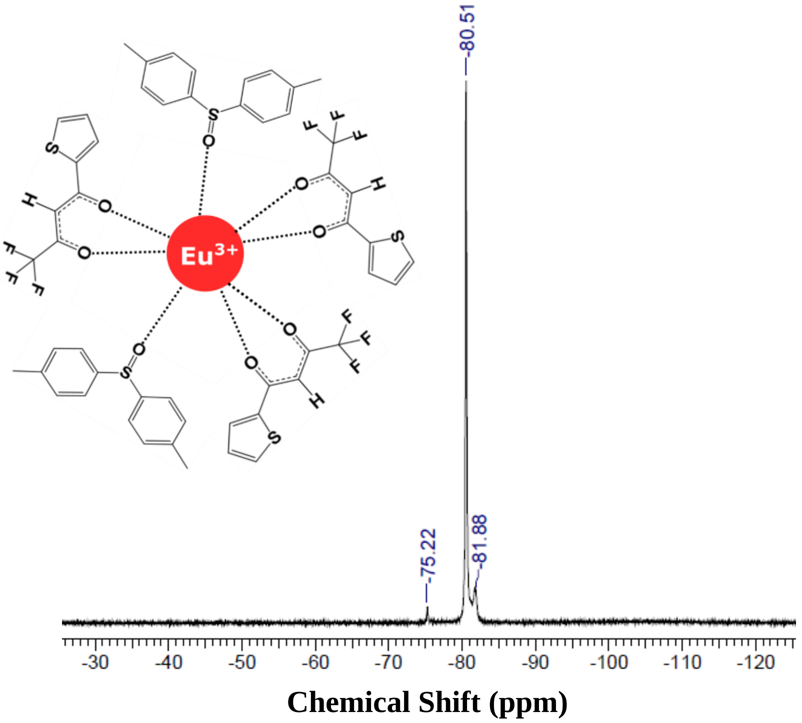

^{19}F NMR (400 MHz, $CDCl_3$): δ -75.22 to -80.51 ppm (CF_3).

S61 Fig. ^{19}F NMR spectrum of $Eu(TTA)_3(PTSO)_2$ of the faster synthesis.

^{31}P NMR Spectra

S62-S67 Figs show ^{31}P NMR spectra for the TPPO free ligand and for the all synthesized complexes with TPPO ligand.

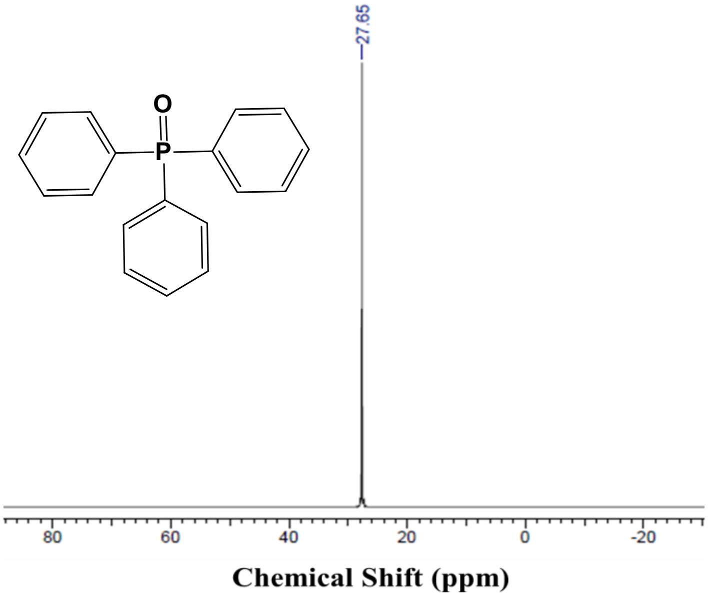

^{31}P NMR (400 MHz, $CDCl_3$): δ 28 ppm.

S62 Fig. ^{31}P NMR spectrum of TPPO free ligand.

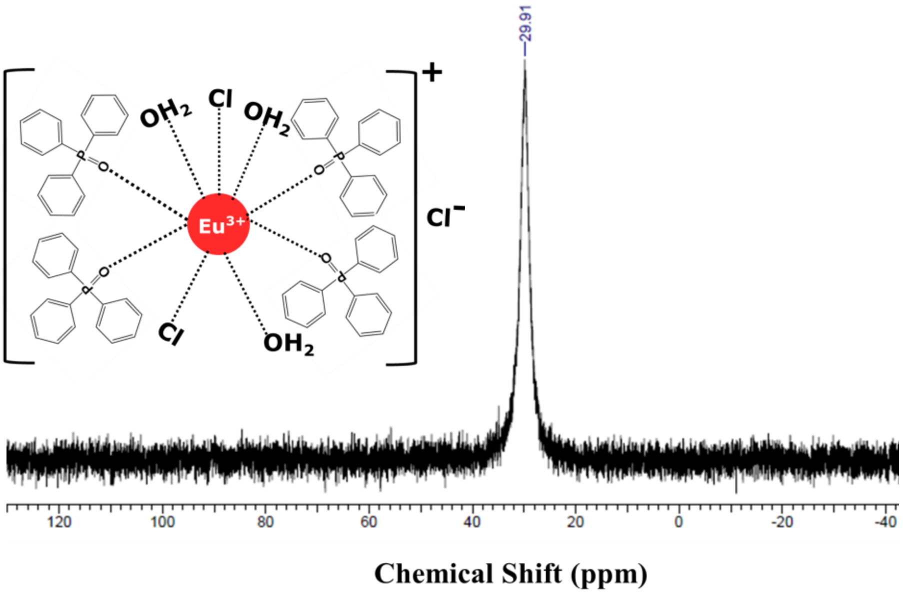

^{31}P NMR (400 MHz, $CDCl_3$): δ 30 ppm.

S63 Fig. ^{31}P NMR spectrum of $EuCl_3(TPPO)_4(H_2O)_3$.

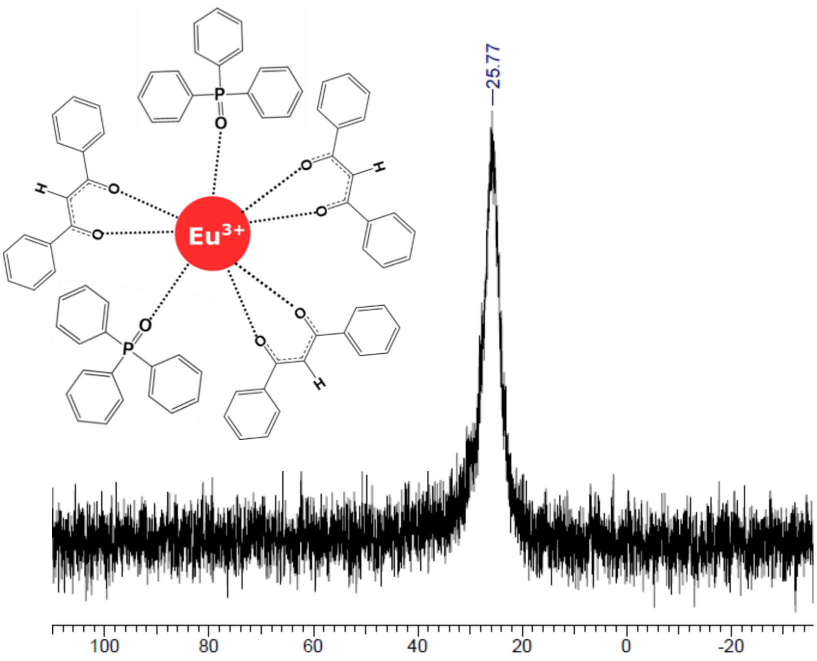

Chemical Shift (ppm)

^{31}P NMR (400 MHz, $CDCl_3$): δ 26 ppm.

S64 Fig. ^{31}P NMR spectrum of $Eu(DBM)_3(TPPO)_2$ of the usual synthesis.

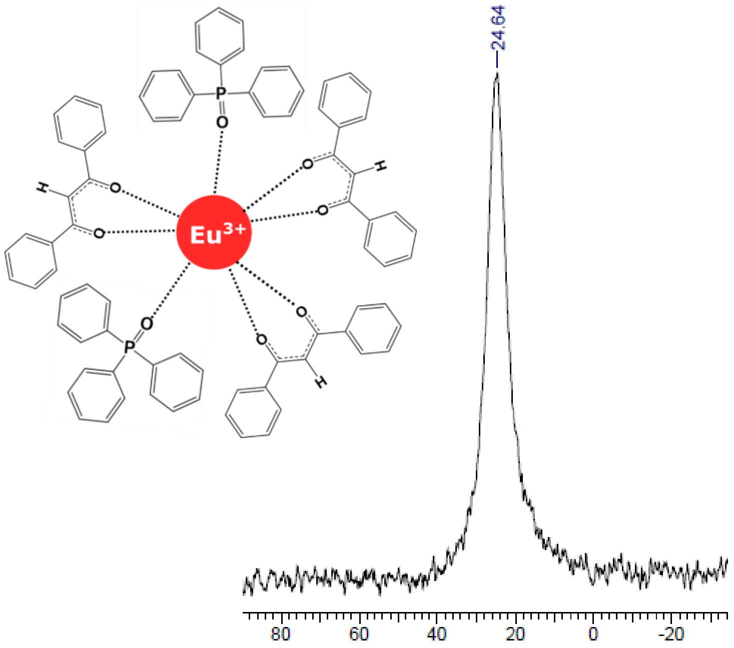

Chemical Shift (ppm)

^{31}P NMR (400 MHz, $CDCl_3$): δ 25 ppm

S65 Fig. ^{31}P NMR spectrum of $Eu(DBM)_3(TPPO)_2$ of the faster synthesis.

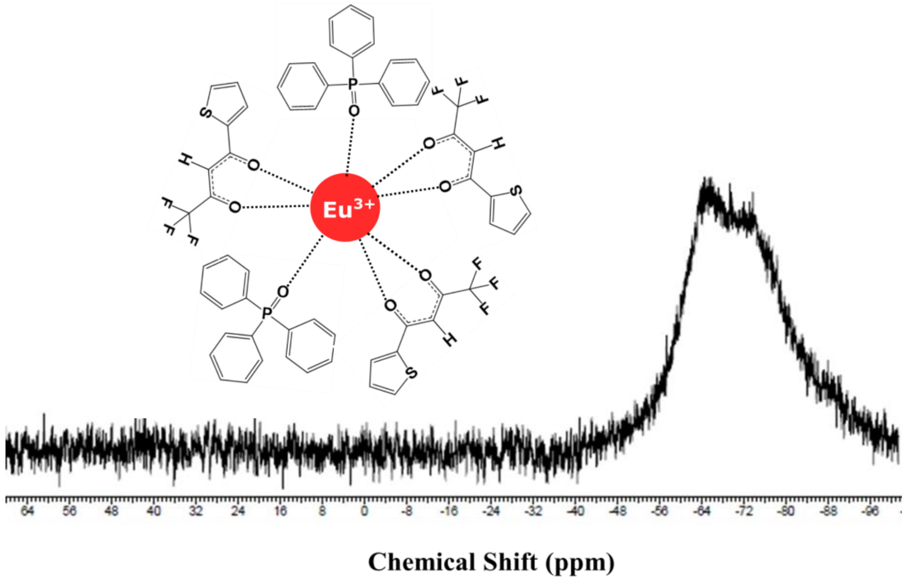

^{31}P NMR (400 MHz, $CDCl_3$): δ -66 ppm.

S66 Fig. ^{31}P NMR spectrum of $Eu(TTA)_3(TPPO)_2$ of the usual synthesis.

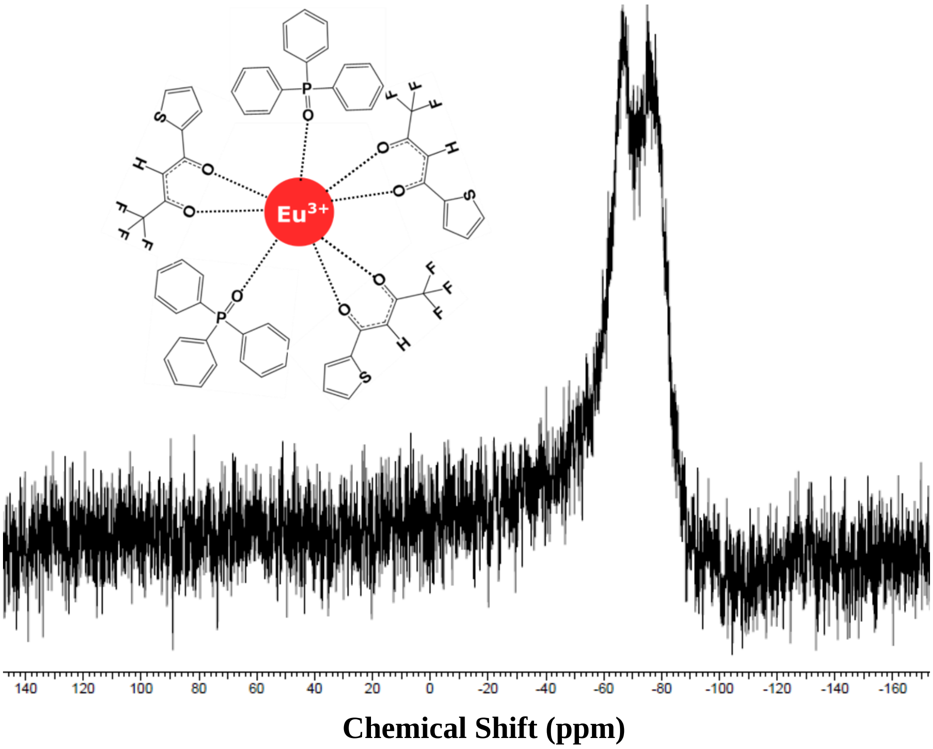

^{31}P NMR (400 MHz, $CDCl_3$): δ -66 ppm.

S67 Fig. ^{31}P NMR spectrum of $Eu(TTA)_3(TPPO)_2$ of the faster synthesis.

Summary of characterizations

S1 Table, below, contains a summary of all characterization data for all free ligands and complexes synthesized in this article.

S1 Table. Summary of characterization data for all complexes synthesized in this article

Free ligand/Complex	IR (cm^{-1})	NMR (ppm)		
		^{1}H	^{19}F	^{31}P
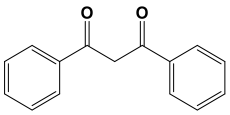<br>DBMH	ν=C-H 3060 cm^{-1}– 3038 cm^{-1}, νC=O 1599 cm^{-1}.	δ8.01 - 6.67 (m, Ar.), 4.69 (m, CH_2).	------	------
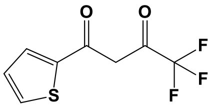<br>TTAH	ν=C-H 3107 cm^{-1}–3087cm^{-1}, νC=O 1655 cm^{-1}.	δ7.62 - 6.43 ppm (m, Ar.), 3.34 ppm (m, CH_2).	δ -75.80	------
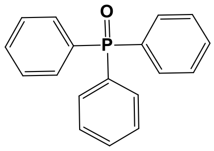<br>TPPO	ν=C-H 3091 cm^{-1} – 3000 cm^{-1}, νP=O 1118 cm^{-1}.	δ7.60 - 7.37 ppm (m, Ar.).	------	δ 28
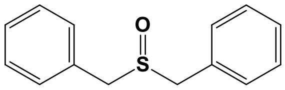<br>DBSO	ν=C-H 3102 cm^{-1}– 3036 cm^{-1}, ν CH_2 2960 cm^{-1}–2913cm^{-1}, νS=O 1032 cm^{-1}.	δ7.36 - 7.30 (m, Ar.), 3.91 -3.88 (m, CH_2).	------	------
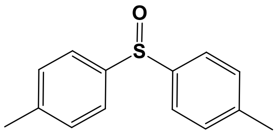<br>PTSO	ν=C-H 3051 cm^{-1}– 3017 cm^{-1}, ν CH_3 2975 cm^{-1}–2862cm^{-1}, νS=O 1037 cm^{-1}.	δ7.48 - 7.22 ppm (m, Ar.), 2.33 ppm (s,CH_3).	------	------
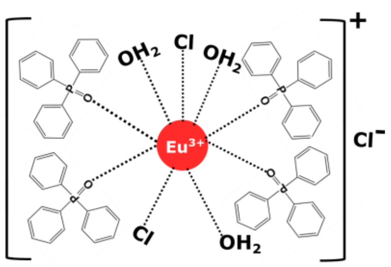<br>$EuCl_3(TPPO)_4(H_2O)_3$	νO-H 3661 cm^{-1},ν=C-H 3092 cm^{-1}– 3017 cm^{-1}, νP=O 1088 cm^{-1}.<br>$[M+H]^+$ (M/Z) = 1425.22	δ 7.43 (m, Ar.) and 2.61 (s,OH)..	------	δ 30

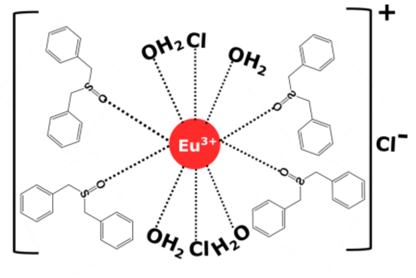<br>$EuCl_3(DBSO)_4(H_2O)_4$	νO-H 3407 cm^{-1}, νS=O 1031 cm^{-1}.<br>$[M+H]^+$ (M/Z) = 1251.15	δ7.38-7.31 (m, Ar.), 3.90 (m,CH_2) and 2.21 (s,OH)..	------	------
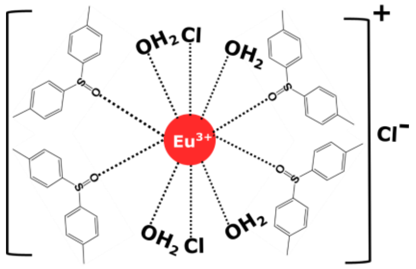<br>$EuCl_3(PTSO)_4(H_2O)_4$	νO-H 3704 cm^{-1}, νS=O 1036 cm^{-1}.<br>$[M+H]^+$ (M/Z) = 1251.16	δ7.51-7.24 (m, Ar.), 2.34 (s, CH_3) and 2.07 (s,OH).	------	------
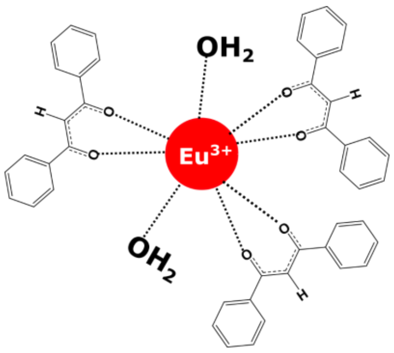<br>$Eu(DBM)_3(H_2O)_2$	ν O-H 3604,ν (=C-H) 3060 – 3015 cm^{-1}, ν C=O 1596 cm^{-1}.	δ 16.66 (s,CH), 8.01-7.29 (m, Ar.).	------	------
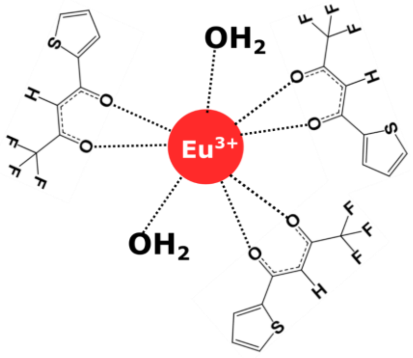<br>$Eu(TTA)_3(H_2O)_2$	ν=O-H 3394 cm^{-1}, νC=O 1617 cm^{-1}.	δ 10.79 (s,CH),7.03-5.30 (m, Ar.).	δ -80.77	------

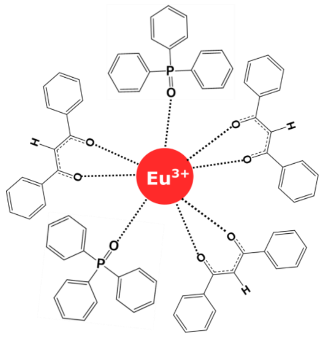 Eu(DBM)$_3$(TPPO)$_2$ by usual synthesis	ν(=C-H) 3082-3027 cm^{-1}, νC=O 1599 cm^{-1}, νP=O 1070 cm^{-1}.	δ 17.14 (s,CH), 8.28-7.03 (m, Ar.).	------	δ 26
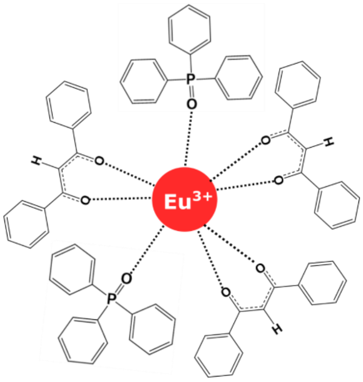 Eu(DBM)$_3$(TPPO)$_2$ by faster synthesis	ν=C-H 3067 cm^{-1} – 3020 cm^{-1} , ν C=O 1597 cm^{-1}, νP=O 1074 cm^{-1}.	δ 17.15 (s,CH), 8.28-7.83 (m, Ar.).	------	δ 25
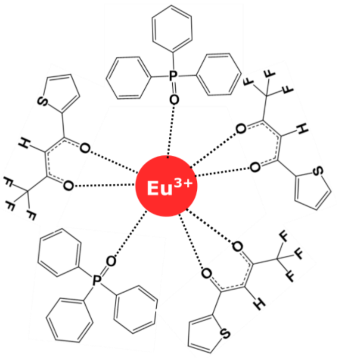 Eu(TTA)$_3$(TPPO)$_2$ by usual synthesis	ν=C-H 3102 cm^{-1} – 3032 cm^{-1} , ν C=O 1608 cm^{-1}, νP=O 1065 cm^{-1}.	δ 9.89 (s,CH), 7.71-6.04 (m, Ar.).	δ-80.03 to -80.69 ppm (CF_3).	δ -66
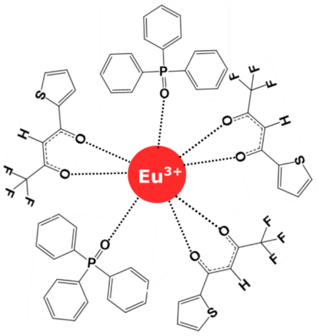 Eu(TTA)$_3$(TPPO)$_2$ by faster synthesis	ν=C-H 3104 cm^{-1} – 3030 cm^{-1} , ν C=O 1607 cm^{-1}, νP=O 1060 cm^{-1}.	δ 9.09 (s,CH), 7.66-6.01 (m, Ar.).	δ -79.97 to -82.68 ppm (CF_3).	δ -66

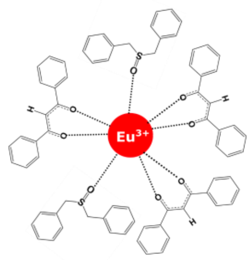 $Eu(DBM)_3(DBSO)_2$ by usual synthesis	ν(=C-H) 3055 -3018cm^{-1}, ν CH_2 2956 cm^{-1}, ν C=O 1594 cm^{-1}, νS=O 1022 cm^{-1}.	δ 16.79 (s,CH), 7.93-7.19 (m, Ar.) and 4.00 (m,CH_2).	------	------
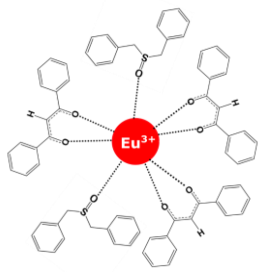 $Eu(DBM)_3(DBSO)_2$ by faster synthesis	ν=C-H 3083 cm^{-1} – 3032 cm^{-1} , ν CH_2 2960 cm^{-1} - 2913 cm^{-1}, ν C=O 1599 cm^{-1}, νS=O 1028 cm^{-1}.	δ 16.80 (s,CH), 7.83-7.19 (m, Ar.) and 4.72 (m,CH_2).	------	------
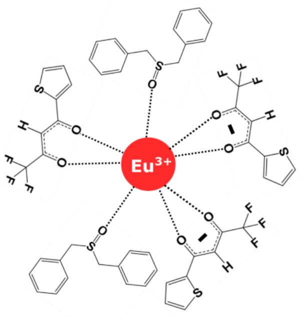 $Eu(TTA)_3(DBSO)_2$ by usual synthesis	ν=C-H 3107 cm^{-1} – 3031 cm^{-1} , ν CH_2 2975 cm^{-1} - 2919cm^{-1}, ν C=O 1606 cm^{-1}, νS=O 1012 cm^{-1}.	δ 12.42 (s,CH), 8.76 - 6.03 (m, Ar.) and 2.65-2.52 (m,CH_2).	δ -74.98 to 80.49 ppm (CF_3)	------
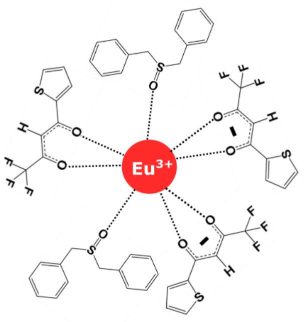 $Eu(TTA)_3(DBSO)_2$ by faster synthesis	ν=C-H 3088 cm^{-1} – 3032 cm^{-1} , ν CH_2 2975 cm^{-1} - 2918 cm^{-1}, ν C=O 1607 cm^{-1}, νS=O 1013 cm^{-1}.	δ 11.64 (s,CH), 7.68 – 6.22 (m, Ar.) and 4.54-4.44 (m,CH_2).	δ –75.06 to -82.18 ppm (CF_3).	------

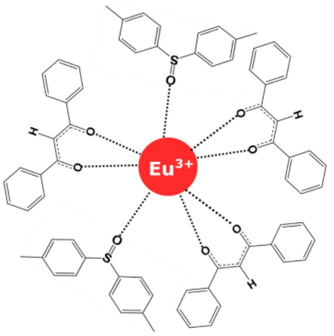 $Eu(DBM)_3(PTSO)_2$ by usual synthesis	ν (=C-H) 3060 – 3013 cm^{-1} ,ν CH_3 2980 - 2861 cm^{-1},ν C=O 1595 cm^{-1}, νS=O 1012 cm^{-1}.	δ 16.80 (s,CH) 7.93- 7.25 (m, Ar.) and 2.32 (m,CH_3).	------	------
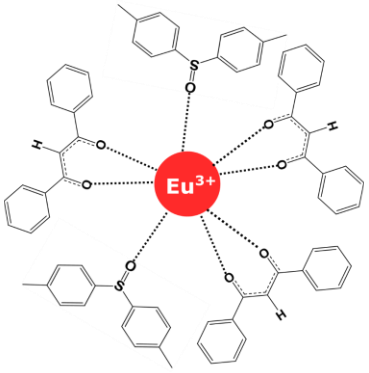 $Eu(DBM)_3(PTSO)_2$ by faster synthesis	ν=C-H 3059 cm^{-1} – 3027 cm^{-1} , ν CH_3 2947cm^{-1} - 2857cm^{-1}, ν C=O 1599 cm^{-1}, νS=O 1018 cm^{-1}.	δ 16.79 (s,CH) 7.96- 7.24 (m, Ar.) and 2.32 (m,CH_3).	------	------
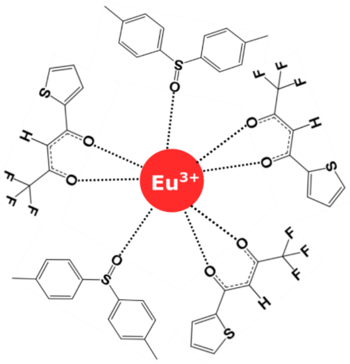 $Eu(TTA)_3(PTSO)_2$ by usual synthesis	ν=C-H 3097 cm^{-1} – 3021 cm^{-1} , ν CH_3 2975 cm^{-1} - 2866 cm^{-1}, ν C=O 1603 cm^{-1}, νS=O 1022 cm^{-1}.	δ 10.45 (m,CH_3), 7.68- 5.62 (m, Ar.) and 2.57 (m,CH_3).	δ -74,45 to -80.79 ppm (CF_3).	------
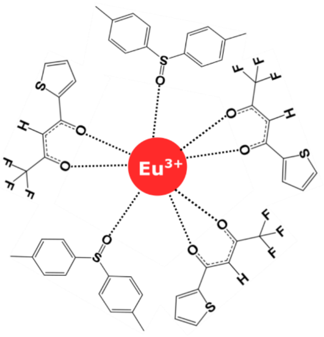 $Eu(TTA)_3(PTSO)_2$ by faster synthesis	ν=C-H 3102 cm^{-1} – 3032 cm^{-1} , ν CH_3 2975 cm^{-1} - 2871 cm^{-1}, ν C=O 1604 cm^{-1}, νS=O 1012 cm^{-1}.	δ 9.43 (m,CH_3), 7.49- 5.66 (m, Ar.) and 2.47 (m,CH_3).	δ -75.22 to -80.51 ppm (CF_3).	------
